# Enabling a Diversity‐Oriented Catalytic Atom Looping of a Biobased Polycarbonate

**DOI:** 10.1002/cssc.202501905

**Published:** 2025-10-06

**Authors:** Enrico Lanaro, Thirusangumurugan Senthamarai, Stephen K. Hashmi, Arjan W. Kleij

**Affiliations:** ^1^ Institute of Chemical Research of Catalonia (ICIQ) The Barcelona Institute of Science and Technology Av. Països Catalans 16 Tarragona 43007 Spain; ^2^ Departament de Química Física i Inorgánica Universitat Rovira i Virgili Marcel·lí Domingo s/n Tarragona 43007 Spain; ^3^ Institut für Organische Chemie Heidelberg University Im Neuenheimer Feld 270 69120 Heidelberg Germany; ^4^ Catalan Institute of Research and Advanced Studies (ICREA) Pg. Lluis Companys 23 Barcelona 08010 Spain

**Keywords:** carbon dioxide, diversity oriented synthesis, homogeneous catalysis, polycarbonates, upcycling

## Abstract

Herein, it is demonstrated that biobased poly(menthene carbonate) (**PMC**) can be conveniently used to enable catalysis‐promoted atom looping thereby creating functionalized synthons and new types of repolymerizable monomers. The biobased polycarbonate undergoes chemoselective depolymerization in the presence of a bicyclic guanidinium providing under distinct reaction conditions and concentrations high‐yield and selective access to either menthene oxide (**MO**), menthene carbonate (**MC**), or menthene diol (**MD**). These latter depolymerization products further enable the valorization of the original polymer atoms into several functionalized, partially biobased building blocks by integrating a monomer‐and‐molecular loop approach. As a proof‐of‐principle, four distinct scaffolds were converted into novel (bifunctional) monomers with potential to create a wider series of macromolecules. This work exemplifies a unique and holistic catalytic reuse of polymer atoms accommodating an on‐demand preparation of fine‐chemical precursors, repolymerizable monomers, and new monomer precursor designs.

## Graphical abstract

A biobased polycarbonate shows susceptibility to switchable and chemoselective organocatalytic degradation into three primary products (P1–P3) that in turn can be further upgraded into functionalized synthons and polymerizable monomers. Selected members of these compounds have been transformed into new types of bifunctional monomers paving the way to a novel approach on the road to a holistic atom looping.

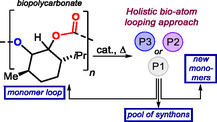

## 1 Introduction

**Scheme 1:**
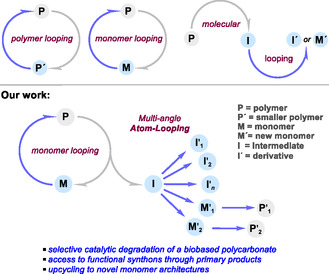
Schematic comparison between known polymer, monomer, and molecular looping, and the newly introduced holistic atom looping approach for PCs.

Plastic pollution and resultant microplastic accumulation in our ecosystems represent a major and global sustainability challenge affecting our societies in terms of clean water access, the extermination of many current species, and human health.^[^ ^1^, ^2^ ^]^ One proposed solution to mitigate these effects is to transition from a conventional linear to a desirable circular use of plastics through a proper postsynthetic and postconsumption recycling approach.^[^ ^3^, ^4^, ^5^, ^6^, ^7^, ^8^, ^9^, ^10^ ^]^ The end‐of‐life mechanical recycling of certain polymers (polymer loop; **Scheme** **1**) such as polyethylene terephthalate (PET) is currently a reasonable and mature technology that has already been implemented in industry.^[^ ^11^ ^]^ However, such polymer loops are restricted to rather pure, single‐component waste streams with high thermal resistance, which upon recycling do not always yield the pristine material properties. Monomer^[^ ^12^ ^]^ and molecular looping (Scheme 1)^[^ ^13^, ^14^ ^]^ offer alternative strategies for partial or full atom reuse, thereby creating opportunities for circular polymers and small molecule repurposing approaches (I → I′ or I → M′, Scheme 1).^[^ ^15^ ^]^ Important requirements to meet efficient atom recycling are the process chemoselectivity and the yield of the polymer degradation product(s).

Unquestionably, there has been a great deal of attention on the production and recycling of polycarbonates.^[^ ^16^, ^17^, ^18^, ^19^ ^]^ Such commodity polymers have advantageous mechanical, thermal, and optical properties making them suitable for many consumer products.^[^ ^20^ ^]^ Alongside, more recent work has shown the recycling potential of aliphatic polycarbonates prepared via either ring‐opening polymerization (ROP) or ring‐opening copolymerization (ROCOP), with prominent examples demonstrating polymer‐to‐monomer conversion and repolymerization to close the cycle.^[^ ^21^, ^22^, ^23^, ^24^ ^]^ In this realm, the use of biomass‐derived monomers has become a primary objective as it facilitates the creation of, upon polymerization, new types of functional and architectural diverse polycarbonates.^[^ ^25^, ^26^ ^]^ Of special mention are those polycarbonates forged from terpene oxide monomers as they are rather rigid in nature and offer postpolymerization functionalization options. These features enable further development of materials with interesting (thermal) properties reminiscent of those known for commercial polycarbonate obtained from bisphenol A (BPA).^[^ ^27^ ^]^

A key requisite for a biobased polycarbonate to be considered as a useful substitute is its recycling potential. The catalytic and controlled degradation of bioderived poly(limonene carbonate) has been the subject of recent scrutiny,^[^ ^28^, ^29^ ^]^ showing progress within the context of a “monomer loop”. In this case, full and quantitative degradation to limonene oxide was achieved,^[^ ^28^ ^]^ which can be repolymerized in the presence of CO_2_ and a suitable catalyst.^[^ ^30^, ^31^, ^32^, ^33^, ^34^ ^]^ However, further progress in the area has been rather limited, and as far as we are aware, a multipurpose recycling (i.e., catalytic atom‐looping offering varies entries to functional molecules; Scheme 1) of biobased polycarbonates remains undeveloped. This is particularly true for a single‐catalyst‐based switchable depolymerization process. Combining both monomer and molecular/atom loop approaches for biobased polycarbonates would significantly amplify the value of atom recycling and create new opportunities for waste plastics in areas beyond polymer science. However, for such a combined approach to be effective, the parent polycarbonate would need to be degraded selectively. Consequently, this would provide high‐yield access to products that can be easily upgraded to more complex scaffolds through the presence of functional groups expanding the reuse of polymers to a versatile atomic loop. Here we present a proof‐of‐principle approach that considers poly(menthene carbonate) (**PMC**) as an exemplary case, and we report its selective organocatalytic degradation into three major products [menthene oxide (**MO**), menthene carbonate (**MC**), and menthene diol (**MD**)] that can be isolated in high yields. These primary bioderived degradation products can be valorized into a range of functional synthons, and four selected scaffolds are shown to be precursors for bifunctional monomer designs creating access to novel biobased architectures.

## 2 Results and Discussion

### 2.1 PMC Synthesis

We selected the preparation of **PMC** as a polymer target for various reasons. First, its preparation so far has only been achieved once using 2‐menthene oxide and CO_2_ in a formal ring‐opening copolymerization (ROCOP) catalyzed by a Zn(bis‐diiminate) complex.^[^ ^35^ ^]^ Second, **PMC** is based on a bioderived terpene oxide and thus represents an interesting feedstock for the creation of new synthetic intermediates with a high bio‐atomic content. Third, based on our recent experience with poly(limonene carbonate) and its partially (60%) selective catalytic degradation to a (ring‐open polymerizable) *trans*‐configured cyclic carbonate,^[^ ^29^ ^]^ we wondered if a structurally related terpene‐derived polycarbonate (**PMC**) could be upgraded with a higher degree of switchable chemoselectivity. More specifically, if a single catalyst could give access to various degradation products under slightly different process conditions retaining high chemoselectivity, there would be a way to create a unique atom‐looping opportunity empowered by the introduction of suitable functional groups in these primary depolymerization outputs.

We started with the ROCOP of 2‐menthene oxide (**MO**) and CO_2_ (**Table** **1**; entries 1–11) using **Al** ^**Me**^/PPNCl as a binary catalyst system in toluene at various temperatures and pressures. Using catalytic conditions previously optimized for the ROCOP of limonene oxide (LO) and CO_2_ (entry 1),^[^ ^29^ ^]^ we found that **PMC** of relatively low molecular weight (*M*_n_ = 2.8 kg mol^−1^) was formed at low **MO** conversion (21%) after 24 h. Next, we examined longer reaction times (entries 2 and 3), showing that after 72 h, the **MO** conversion reached 51% with an isolated yield of 37% for **PMC** and a slightly better *M*_n_ value of 3.8 kg mol^−1^. We scrutinized the other conditions (catalyst/initiator loadings, neat conditions, and varying reaction times and temperatures) in order to improve on this ROCOP process (entries 4–11). We finally found that the best quality **PMC** can be attained at 60 °C and 15 bar pressure if the ROCOP of **MO** and CO_2_ is carried out for 24 h in toluene (entry 9), providing **PMC** with an *M*_n_ of 7.0 kg mol^−1^ and a low polydispersity (*Ð* = 1.22).

**Table 1:** ROCOP of 2‐menthene oxide (MO) and CO_2_ catalyzed by **Al^Me^**/PPNCl, and ROP of *trans* 2‐menthene carbonate (MC) using TBD/BnOH.

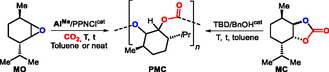					
Entrya)	Al^Me^/PPNCl [mol%]	t [h]	T/P [°C, bar]	Conv. [%]b)	*M*_n_/*Ð* c)
1	1.0, 0.50	24	45, 15	21 (13)	2.8, 1.16
2	1.0, 0.50	48	45, 15	40 (31)	2.9, 1.26
3	1.0, 0.50	72	45, 15	51 (37)	3.8, 1.25
4	1.0, 0.25	72	45, 15	26 (18)	3.0, 1.22
5	0.50, 0.50	72	45, 15	33	2.9, 1.27
6	1.0, 0.50	72	45, 30	64	4.2, 1.28
7d)	1.0, 0.50	24	45, 15	26	3.5, 1.23
8d)	1.0, 0.50	72	45, 15	56	5.1, 1.32
**9**	**1.0, 0.50**	**24**	**60, 15**	**60**	**7.0, 1.22**
10	1.0, 0.50	24	30, 15	8	2.6, 1.10
11d)	1.0, 0.50	24	60, 15	70	3.7, 1.23

Entrya)	TBD/BnOH [mol%]	t [h]	T [°C]	Conv. [%]b)	*M*_n_/*Ð* c)
12	2.0, 2.0	48	80	91	2.8, 1.64
13	1.0, 1.0	48	80	76	3.0, 1.61
14	2.0, 1.0	48	80	92	3.2, 1.67
15	2.0, 1.0	24	80	89	3.2, 1.66
16	2.0, 1.0	48	50	79	3.3, 1.47
17	2.0, 1.0	24	50	52	2.4, 1.21
18	2.0, 1.0	24	23	32	2.1, 1.11
**19** e)	**2.0, 0**	**24**	**80**	**91 (84)**	**5.9, 1.76**
20e)	2.0, 0	5	80	86	4.3, 1.80
21f)	2.0, 0	24	80	92 (85)	4.7, 1.61
22d)	2.0, 0	24	80	77	3.3, 1.94

a) ROCOP experiments were carried out using 1.5 g of **MO**, using toluene (0.38 mL) at the indicated temperature (T, in °C), pressure (P, in bar), and reaction time (t, in hours); ROP experiments were done with *trans*‐**MC** (80 mg) in toluene (1 m) using the indicated amounts for TBD and BnOH, t, and T. *M*_n_ is the number average molecular weight (kg mol^−1^) and the *Ð* the polydispersity;

b) Conversions measured by ^1^H NMR (CDCl_3_), in brackets the yield of the isolated polymer by precipitation in acidified MeOH (1 m);

c) Data obtained by GPC analysis in THF using polystyrene standards;

d) Neat conditions;

e) Reaction carried out in toluene at 4 m;

f) Reaction carried out in toluene at 8 m concentration.

Inspired by our former experience with the ring‐opening polymerization (ROP) of *trans* limonene carbonate by using 1,5,7‐triazabicyclo[4.4.0]dec‐5‐ene (TBD) as an organocatalyst and BnOH as an initiator,^[^ ^29^ ^]^ we also performed the ROP of *trans* menthene carbonate **MC** (prepared directly from the *trans*‐diol of 2‐menthene; see the Supporting Information for details). The screening of various conditions (Table 1; entries 12–22) was carried out including different (relative) loadings of TBD/BnOH, reaction temperatures,^[^ ^36^ ^]^ and process times. Entries 12–18 show that there is no significant influence of these parameters on the reaction outcome, except for the reaction temperature (entries 17 and 18), with 80 °C as the most optimal for the ROP of **MC**. Unexpectedly, we found that in the absence of BnOH initiator (entries 19–22), the ROP of **MC** is not only feasible but also produces significantly higher molecular weight **PMC** (entry 19: *M*_n_ = 5.6 kg mol^−1^, *Ð* = 1.76) at high **MC** conversion (91%) after 24 h.^[^ ^37^ ^]^ Compared to the best ROCOP conditions reported in entry 9, the best ROP conditions for **MC** deliver **PMC** with a slightly lower molecular weight and a significantly higher *Ð* value. Therefore, we used the **PMC** prepared by ROCOP as a starting point for the controlled catalytic depolymerization studies and focusing on a high‐yield synthesis of the degradation products.

### 2.2 Catalytic Depolymerization of PMC

**Scheme 2:**
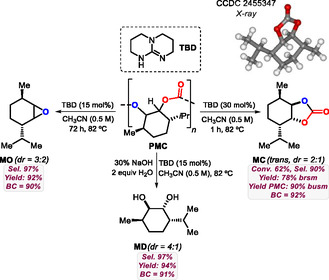
Optimized reaction conditions for the chemoselective depolymerization of **PMC** into **MO**, **MC**, and **MD** using TBD as catalyst. Reported yields are of the isolated products. The inset in the upper right corner shows the X‐ray molecular structure of *trans*‐**MC**.^[^ ^20^ ^]^ For simplicity, only one diastereoisomeric product (*major*) structure is shown.

The **PMC** from entry 9 of Table 1 (*M*_n_ = 7.0 kg mol^−1^, *Ð* = 1.22) was subjected to various reaction conditions using CH_3_CN as a solvent and TBD as a catalyst (**Scheme** **2**, for a complete overview of the results, see the Supporting Information).

We found that at a loading of 15 mol% TBD and a concentration of 0.5 m of the polymer in the medium, **PMC** could be fully depolymerized under gentle reflux after 72 h into **MO** (selectivity: 97%, bio‐content, BC, of the **MO** produced was 90%).^[^ ^38^ ^]^ The **MO** was isolated in 92%, and in 90% when the reaction was scaled up 6 times (500 mg of **PMC** used).

At the same molarity (using 30 mol% TBD) and under reflux, **PMC** was depolymerized (62% conversion) into *trans* MC (90% selectivity, 56% NMR yield, 78% isolated *brsm*; the BC of **MC** is 92%).^[^ ^39^ ^]^ The remaining 32% of **PMC** could be mostly recovered (90% *busm*)^[^ ^39^ ^]^ thus showing overall good atom‐efficiency. The scale up of this optimized process (at 500 mg **PMC**) delivered MC in 72% yield *brsm* and a 95% recovery yield of **PMC** *busm*.

Finally, we were interested in whether we could selectively depolymerize **PMC** into its *trans* *syn‐*diol (**MD**) using TBD as catalyst, as the alcohol groups would offer another type of functionality compared to those present in **MO** and **MC**. We found that combining **PMC** with 2 molar equiv of H_2_O in the presence of NaOH as an additive (30 mol%) and TBD as catalyst (15 mol%) produced after 48 h under reflux **MD** as the primary product (94% isolated yield, BC: 91%). Scale up, as mentioned for the other two products, delivered **MD** in 94% yield using the approach reported by Greiner.^[^ ^35^ ^]^

The combined results demonstrate that by slightly modifying the process conditions, **PMC** can be selectively degraded into three functionalized products containing either a versatile oxirane, a *trans* cyclic carbonate, or *trans* *syn*‐diol unit with an overall high BC and synthetic potential to be further upgraded into a wide variety of synthons thereby creating a multipurpose atom loop based on a single biobased polycarbonate.

### 2.3 Diversification Studies with MO, MC, and MD

**Scheme 3:**
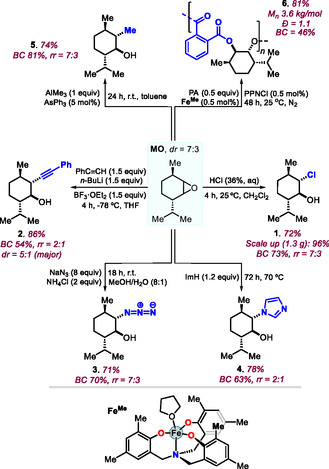
Product diversification studies with **MO** toward functional products **1**–**6**. For simplicity, only one diastereo‐ and regio‐isomeric product structure is shown where applicable. ImH stands for imidazole, PA stands for phthalic anhydride, PPNCl is bis(triphenylphosphine)iminium chloride, and the structure for **Fe** ^**Me**^ is given below the scheme.

With three different functional groups present in the primary degradation products, we set out to diversify the access to synthons with a partial biocontent. Starting from **MO** (*dr* = 7:3, **Scheme** **3**), nucleophilic ring opening of the oxirane unit with aqueous HCl provided access to chlorohydrin **1** in 72% yield (*rr* = 7:3, similar to the starting *dr* value of **MO**). The same compound could also be accessed at a larger scale (1.3 g **MO** used) giving **1** in an almost quantitative yield of 96%. By further elaborating on epoxide ring‐opening chemistry, alkynylated compound **2** (86%, *rr* = 2:1) was prepared by treatment of **MO** by an in situ generated phenyl acetylide, while 1,2‐azido‐alcohol **3** (71%, *rr* = 7:3) was generated in the presence of sodium azide in a basic regime. Other *N*‐based nucleophiles such as imidazole are also feasible reagents and provide access to, for instance, 1,2‐amino‐alcohol **4** (78%, *rr* = 2:1). Carbon‐based nucleophilic reagents such as AlMe_3_ allow to formally alkylate **MO** thereby forming alcohol **5** in 74% yield (*rr* = 7:3). Lastly, as **MO** represents a potentially useful epoxy monomer, we decided to demonstrate that the original **PMC** atoms can also be recycled to form the oligoester **6** (81%, *M*_n_ = 3.6 kg mol^−1^, *Ð* = 1.1) by ROCOP with phthalic anhydride (PA) under Fe‐catalysis.^[^ ^40^ ^]^ All products **1**–**6** have an appreciable to high bio‐content (BC) of 46−81% originating from **PMC** (BC = 92%).

**Scheme 4:**
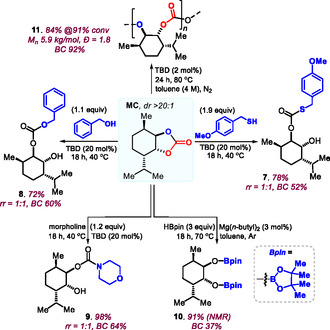
Product diversification studies with **MC** toward functional products **7**–**11**. For simplicity, only one diastereo‐ and regio‐isomeric product structure is shown where applicable.

Next, we examined the valorization of **MC** (*dr* > 20:1, **Scheme** **4**) using various ring‐opening transformations of the *trans*‐configured bicyclic carbonate. Various *S*‐, *O*‐ and *N*‐based nucleophiles can be used to create either a thiocarbonate (**7**: 78%, *rr* = 1:1, BC = 52%), a linear carbonate (**8**: 72%, *rr* = 1:1, BC = 60%), or a carbamate (**9**: 98%, *rr* = 1:1, BC = 64%). In the case of the thioester, a larger excess of thiol reagent was needed to ensure high conversion of the carbonate precursor. Nonsymmetrical versions of bio‐esters such as **7**–**9** are typically not easily accessed. In order to further vary the synthetic utility of **MC**, a hydroboration was probed using HBpin under Mg‐catalysis.^[^ ^41^ ^]^ A bis‐borate ester (**10**: 91%, BC = 37%) was hence accessible, which can offer a useful entry to Lewis acids for epoxide activation and conversion.^[^ ^42^ ^]^ As a final synthetic outlet for **MC**, its direct recycling into **PMC** (**11**: 91% MC conversion, 84%, BC = 92%: see entry 19 in Table 1) via ROP under TBD catalysis provides a simple, though effective monomer looping process.

**Scheme 5:**
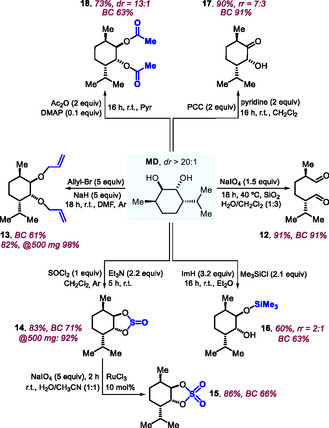
Product diversification studies with **MD** toward functional products **12**–**18**. For simplicity, only one diastereo‐ and regio‐isomeric product structure is shown where applicable. ImH stands for imidazole.

With both **MO** and **MC** being used in various atom‐valorization processes, we then turned our focus on **MD** (*dr* >20:1, **Scheme** **5**).^[^ ^43^ ^]^ The first trial was the oxidative cleavage of the *syn*‐diol into bis‐aldehyde **12** in 91% yield (BC = 91%). Allylation of the same diol using allyl bromide under basic conditions at r.t. afforded the bis‐allyl ether **13** (BC = 61%) in 82% yield, which could be improved to 98% when the reaction was scaled up to 500 mg of **MD**. The cyclic sulphite **14** was produced in 83% yield by treatment of **MD** with thionyl chloride, and again with an improved yield at a larger scale (92% at 500 mg **MD**; BC = 71%). The Ru‐catalyzed oxidation of cyclic sulphite **14** utilizing NaIO_4_ as oxidant^[^ ^44^ ^]^ produced cyclic sulphate **15** in 86% yield. Compounds such as **15** can be easily ring‐opened and converted into aziridines through a formal desulphonative amination.^[^ ^44^, ^45^, ^46^ ^]^ Mono‐silylation of **MD** afforded the silylether **16** in 60% yield (*rr* = 2:1, BC = 63%), while the mono‐ketone **17** (90%, *rr* = 7:3, BC = 91%) was furnished when **MD** was exposed to a mixture of pyridinium chlorochromate/pyridine under ambient conditions. Finally, the bis‐*O*‐acetyl derivative **18** (73%, BC 63%) was prepared in the presence of acetic anhydride in a basic medium. The combined pool of functionalized synthons from **MO**, **MC**, and **MD** allows for follow‐up transformations, and specifically the design of new kinds of partially biobased monomers for a range of macromolecules (*vide infra*).

### 2.4 Design of New Monomers

**Scheme 6:**
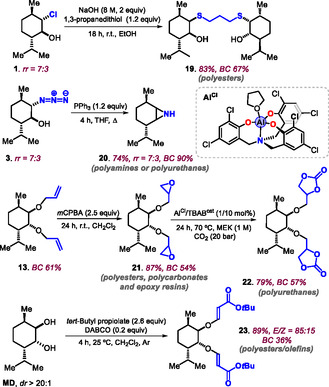
New monomer designs using selected synthons from the pool of synthons **1**–**18**.

The presence of particular functional groups allows to create new types of monomers (**Scheme** **6**). For instance, chlorohydrin **1** is simply transformed into bis‐thioether **19** (83%, BC = 67%) by a double nucleophilic substitution using 1,3‐propanedithiol under basic conditions. The latter product can be regarded as a partially biobased diol useful to create polyesters by step‐growth polymerization when combined with dicarboxylic acids, or via acid‐catalyzed transesterification of known polyesters. The azido‐alcohol **3** could be easily converted into aziridine **20** in 74% yield (BC = 90%) by adding PPh_3_ to a THF solution of **3**. Aziridines are potential monomers toward the creation of polyamines via ROP, and polycarbamates via ROCOP in the presence of CO_2_.^[^ ^47^ ^]^ The bis‐allyl ether **13** could be epoxidized affording this bis‐epoxide **21** in 87% yield (BC = 54%), offering a bifunctional monomer for either branched polyesters or polycarbonates via suitable ROCOP partners,^[^ ^48^ ^]^ or epoxy‐based resins using polyamines.

Alternatively, the bis‐epoxide **21** can be turned into its bis‐cyclic carbonate **22** (79%, BC = 57%) through binary [Al]/bromide catalysis at 70 °C in MEK as a medium.^[^ ^49^, ^50^ ^]^ The bis‐carbonate offers a tangible starting point for the creation of (partially) biobased polyurethanes via formal aminolysis using suitable bis‐amine reagents.^[^ ^51^, ^52^ ^]^ Finally, when **MD** is treated directly with *tert*‐butyl propiolate in the presence of a catalytic amount of DABCO (triethylenediamine), a double *oxa*‐Michael addition is provoked leading to bis‐acrylic ether derivative **23** in 89% yield with potential value in radical‐initiated polymerization processes.^[^ ^53^ ^]^ Though the functional monomers of Scheme 6 are mixtures of regio/stereo‐isomers, this aspect is deemed not crucial when targeting the creation of amorphous polymers.

## 3 Conclusion

In summary, we here describe a catalysis‐driven, generic reuse of a biobased polycarbonate (**PMC**) that allows access to three primary degradation products (**MO**, **MC**, and **MD**) in a chemoselective manner and in high yield using a switchable organocatalyst. This switchable catalytic nature while maintaining high product yields is different from the typical singular molecular focus reported for the catalytic depolymerization of PCs based on cyclohexene/cyclopentene oxide,^[^ ^9^, ^10^, ^54^, ^55^ ^]^ propylene oxide,^[^ ^56^ ^]^ and other monomers.^[^ ^9^, ^11^, ^57^ ^]^ The primary synthons derived from **PMC** can subsequently be valorized into functionalized scaffolds and new types of monomers serving a wider range of polymer synthesis. Our work exemplifies how different, complementary looping approaches can be unified into an attractive type of holistic atom‐circularity, thereby creating new opportunities for fine chemical synthesis and polymer development.

## Conflict of Interest

The authors declare no conflict of interest.

## Supporting information

Supplementary Material

## Data Availability

The data that support the findings of this study are available from the corresponding author upon reasonable request.
