# Supplementary material for: Enabling a Diversity‐Oriented Catalytic Atom Looping of a Biobased Polycarbonate
Source: ChemSusChem. 2025 Oct 6;18(22):e202501905. doi: 10.1002/cssc.202501905 (PMC12642971; doi:10.1002/cssc.202501905)
Supplement: Supplementary file 1 — Supplementary Material [file CSSC-18-e202501905-s001.pdf]

Supporting Information for:

Enabling a Diversity-Oriented Catalytic Atom Looping of a Biobased Polycarbonate

Enrico Lanaro,[a][b][c] Thirusangumurugan Senthamarai,[a] Stephen K. Hashmi[c] and Arjan W. Kleij*[a][d]

[a] Institute of Chemical Research of Catalonia (ICIQ), the Barcelona Institute of Science and Technology, Av. Països Catalans 16, 43007 Tarragona, Spain

[b] Departament de Química Física i Inorgánica, Universitat Rovira i Virgili, Marcel·lí Domingo s/n, 43007 Tarragona, Spain

[c] Institut für Organische Chemie, Heidelberg University, Im Neuenheimer Feld 270, 69120 Heidelberg, Germany

[d] Catalan Institute of Research and Advanced Studies (ICREA), Pg. Lluis Companys 23, 08010 Barcelona, Spain

*E-mail: akleij@iciq.es

Table of Contents

1. General experimental details 3

 1.1 General remarks 3

 1.2 Reagents and complexes 4

2. Polymerization procedures 5

 2.1 Synthesis of poly(menthene carbonate), PMC, via ROCOP 5

 2.2 Synthesis of poly(menthene carbonate), PMC, via ROP 5

 2.3 Additional information and data for the polymerization studies 6

3. Depolymerization procedures 8

 3.1 General procedure for the TBD-initiated depolymerization of PLC into MO..................... 8

 3.2 General procedure for the TBD-initiated depolymerization of PLC into MC 8

 3.3 General procedure for the TBD-initiated depolymerization of PLC into MC 9

 3.4 Additional information on the depolymerization studies 10

4. Diversification studies using MO as a precursor 20

5. Diversification studies using MC as synthon 23

6. Diversification studies using MD as synthon..................... 25

7. Synthesis of new types of bifunctional monomers 28

8 Characterization data for all compounds 30

9. References 82

1. General experimental details

1.1 General remarks

All oxygen- and water-sensitive operations were carried out under a nitrogen atmosphere using an MBraun glovebox and Schlenk techniques. All reagents were purchased from Aldrich or TCI suppliers and used as received if not stated differently. Solvents were dried using an Innovative Technology PURE SOLV solvent purification system. Benzyl alcohol (BnOH) was dried over calcium hydride (CaH_2) and distilled under reduced pressure.

Nuclear magnetic resonance (NMR) spectroscopy: NMR spectra were obtained on a Bruker Avance II 400 MHz or a Bruker Avance II 500 MHz spectrometer at room temperature in the respective deuterated solvents. ^{1}H and ^{13}C chemical shifts are reported in parts per million (ppm), relative to tetramethylsilane (TMS) for ^{1}H and ^{13}C with the residual solvent peak used as an internal reference. Multiplicities are reported as follows: singlet (s), broad band (br), d (doublet), dd (doublet of doublets), triplet (t) and multiplet (m).

Thermal analyses: Differential scanning calorimetry (DSC) to determine glass transition temperatures (T_g) were measured under an N_2 atmosphere using a Mettler Toledo model DSC822e. Samples were weighed into 40 µL aluminum crucibles and subjected to three heating cycles typically at a heating rate of 10 °C/min. Thermogravimetric analyses (TGA) were recorded under an N_2 atmosphere using Mettler Toledo model TGA/SDTA851. Samples were weighed into 40 µL aluminum crucibles and heated to 600 °C typically at a heating rate of 10 °C/min. All thermal data were collected by the Research Support Unit at ICIQ. For the DSC analyses, typically the data referring to the second heating/cooling were selected to determine the T_g values.

Gel permeation chromatography (GPC) measurements were performed using an Agilent 1200 series HPLC system, equipped with a PSS SDV Analytical linear M GPC column (8 x 300 mm; 5 µm particle size) in tetrahydrofuran (THF) at 30 °C at a flow rate of 1 mL·min^{-1}. Samples were analyzed at a concentration of 1 mg·mL^{-1} after filtration through a 0.45 µm pore-size membrane. M_n, M_w, and $Đ$ data were derived from the RI signal by a calibration curve based on polystyrene standards (PS from Polymer Standards Service) for the analysis of the polymers. The GPC samples were prepared by dissolving the polymer (2−5 mg) in THF (2 mL) and filtering the solution through a 0.45 µm pore-size membrane.

1.2 Reagents and complexes

Menthene oxide (MO) was prepared according to a previously reported procedure,[1] and was used after being stirred over CaH_2 for 24 hours followed by filtration. Bis-(triphenylphosphine)-iminium chloride (PPNCl) was purified by crystallization from chloroform. The Al-complex (**Al^{Me}**)[2] and menthene diol were prepared according to previously reported procedures.[3]

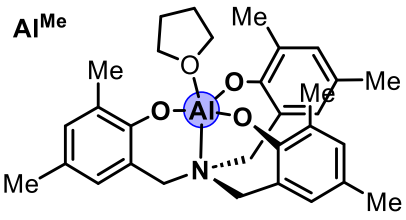

1.3 Menthene cyclic-carbonate (MC) monomer synthesis

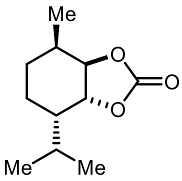

An oven-dried 50 mL flask under argon was charged with menthene diol (**MD**) (11.0 g, 64 mmol, *dr* > 20:1) dissolved in 30 mL of dry DCM. Then, to the reaction mixture (0 °C) were sequentially added pyridine (20 g, 21 mL, 0.26 mol, 4 equiv) and dropwise a solution of triphosgene (9.5 g, 32 mmol, 0.5 equiv) in 80 mL of DCM. The reaction mixture was stirred at 0 °C for 4 hours, and the reaction mixture quenched with a saturated solution of $NaHCO_3$ (150 mL) and diluted with DCM. The organic phase was washed with 1 N HCl (3 × 100 mL), a saturated solution of $NaHCO_3$ (3 × 100 mL) and brine (3 × 100 mL). The organic phase was then dried on $MgSO_4$, filtered and concentrated under reduced pressure. The residue was purified via column chromatography (cyclohexane:ethyl acetate, 99:1 to 95:5), obtaining the product as colorless crystals (9.47 g, 75% yield, *dr* > 20:1). **^{1}H NMR** (400 MHz, $CDCl_3$) δ 4.62 – 4.46 (m, major), 3.92 – 3.85 (m, minor), 3.74 – 3.66 (m, minor), 2.59 – 2.47 (m, 1H), 2.02 – 1.42 (m, 6H), 1.09 – 0.99 (m, 6H), 0.94 – 0.90 (m, 3H). **^{13}C NMR** (101 MHz, $CDCl_3$) δ 154.8, 80.5, 80.0, 43.6, 30.2, 25.3, 24.9, 22.2, 22.0, 21.7, 10.7. **HRMS** (ESI/TOF) *m/z*: [M + Na]$^+$ Calcd for. $C_{11}H_{18}NaO_3$: 221.1150; found 221.1148.

2. Polymerization procedures

2.1 Synthesis of poly(menthene carbonate), PMC, via ROCOP

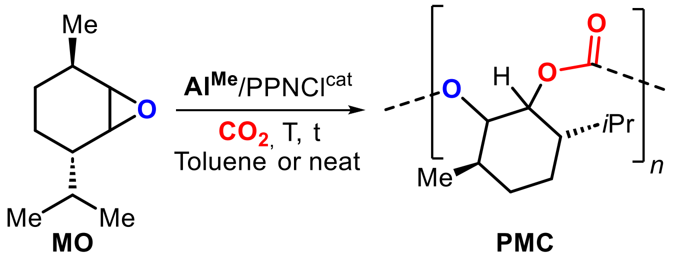

Menth-2-ene oxide synthetized according to a previously reported procedure[1] was dried prior to its use on CaH_2 and filtered. In a nitrogen-filled glovebox, the menth-2-ene oxide, **Al^{Me}**, PPNCl and dry toluene were combined in a Teflon vessel equipped with a cross-shaped magnetic stirring bar, and placed inside a stainless-steel reactor. The reactor was purged three times with 5 bar of CO_2. Finally, the pressure was stabilized at the desired pressure of CO_2 at r.t. After placing the reactor in a metal heating block, the reaction mixture was stirred at the stated temperature and time. The reaction was stopped by cooling the reactor in an ice bath and subsequent applying gentle depressurization. When opened, an aliquot of the product was dissolved in a minimum amount of DCM, and the solvent removed in vacuo following analysis by ^{1}H-NMR analysis to determine the substrate conversion. The remaining reaction mixture was added dropwise to a stirred solution of MeOH/HCl (1 M) (12:1 v/v) causing the **PMC** product to precipitate as a white solid. The precipitate was filtered, washed with cold methanol and dried in vacuo.

2.2 Synthesis of poly(menthene carbonate), PMC, via ROP

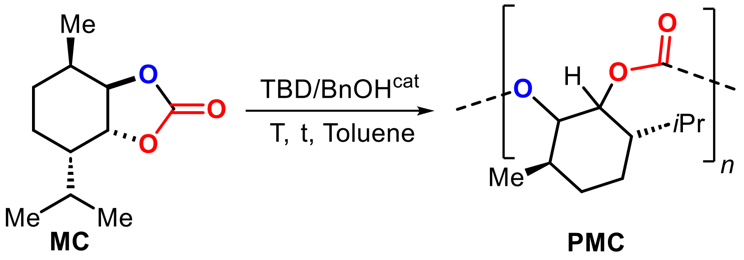

In a nitrogen-filled glovebox, cyclic *trans*-menthene carbonate (80 mg, 0,04 mmol), benzyl alcohol, TBD and toluene were combined in a vial with a Teflon screw cap. The reaction mixture was stirred heated with an oil bath to the desired reaction temperature and kept for the indicated time. The reactions were quenched by cooling to ambient temperature and then taking an aliquot dissolving it a minimum amount of DCM. The latter was removed by evaporation, and the residue analyzed by ^{1}H NMR to determine the conversion. The remaining reaction mixture was added dropwise to a stirred solution of MeOH/HCl (1 M, 7 mL, 12:1 v/v) causing the **PMC** to precipitate as a white solid. This solid was separated via decantation and vacuum dried.

2.3 Additional information and data for the polymerization studies

Ring-opening copolymerization (ROCOP) of menthene oxide (**MO**) and CO_2.

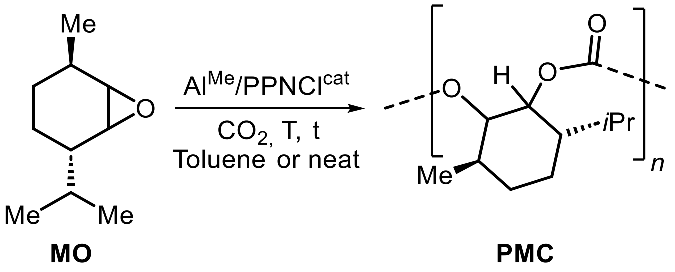

Table S1. Optimization of the reaction conditions for the ROCOP of menthene oxide and CO_2.

Entry[a]	Time (h)	Al-Me (equiv)	PPNCl (equiv)	T (°C)	Conversion (%)[b]	Yield (%)[c]	M_n (Kg/mol)[d]	$Đ$[d]	T_g[e] (°C)	T_d^{5}[f] (°C)
1	72	0,01	0,005	45	20[a]	49	5.5	1,28	136	218
2	72	0,01	0,005	45	51	37	3.8	1.25	126	210
3	24	0,01	0,005	45	21	13	2.8	1.16	99	243
4	48	0,01	0,005	45	40	31	2.9	1.26	109	215
5	72	0.01	0.0025	45	26	18	3.0	1.22	-	-
6	72	0.005	0.005	45	33	-	2.9	1.27	-	-
7[g]	72	0,01	0,005	45	64	-	4.2	1.28	-	-
8[h]	24	0.01	0.005	45	26	-	3.5	1.23	-	-
9[h]	72	0.01	0.005	45	56	-	5.1	1.32	-	-
**10**	**24**	**0.01**	**0.005**	**60**	**60**	**58**	**7.0**	**1.22**	**133**	**225**
11	24	0.01	0.005	30	8	-	2.6	1.10	-	-
12[h]	24	0.01	0.005	60	70	-	3.7	1.23	-	-

[a]Reaction conditions: **MO** = menthene oxide (1.5 g, 9.7 mmol), toluene (0.38 mL), CO_2 (15 bar). [b]Determined by ^{1}H NMR ($CDCl_3$). [c]Isolated yield. [d]Determined by GPC. [e]Determined by DSC analysis. [f]Determined by TGA analysis. [g]CO_2 (30 bar). [h]Neat conditions.

Ring opening polymerization (ROP) study of menthene cyclic-carbonate (**MC**).

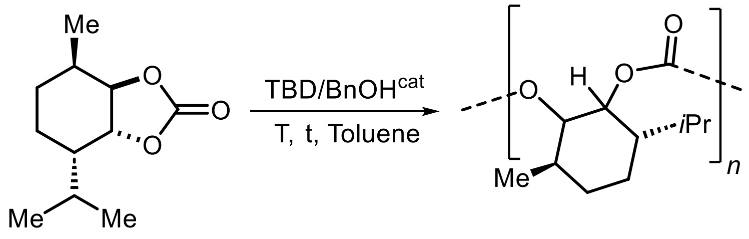

MC **PMC**

Table S2. Optimization of the reaction conditions for the ROP of menthene cyclic-carbonate.

**Entry**[a]	**Time** (h)	**TBD** (equiv)	**BnOH** (equiv)	**T** (°C)	**Conversion** (%)[b]	***M*$_n$** (Kg/mol)[c]	***Đ***[c]
1	48	0.02	0,02	80	91	2.8	1.64
2	48	0.01	0,01	80	76	3.0	1.61
3	48	0.02	0,01	80	92	3.2	1.67
4	48	0.02	0.01	50	79	3.3	1.47
5	24	0.02	0.01	50	52	2.4	1.21
6	48	0.02	0.01	23	32	2.1	1.11
7	48	0.02	0	80	93	3.4	1.87
8	24	0.02	0,01	80	89	3.2	1.66
9	5	0.02	0,01	80	41	2.4	1.24
10	24	0.02	0	80	88	3.4	1.75
**11**[d]	**24**	**0.02**	**0**	**80**	**91**[e]	**5.9**	**1.76**
12[d]	5	0.02	0	80	86	4.3	1.80
13[f]	24	0.02	0	80	77	3.3	1.94
14[g]	24	0.02	0	80	92	4.7	1.61

[a]Reaction conditions: **MC** = menthene cyclic carbonate (80 mg, 0.4 mmol), toluene (1.0 M). [b]Determined by ^{1}H NMR ($CDCl_3$). [c]Determined by GPC. [d]Toluene (4.0 M). [e]Isolated yield = 84%, T_g = 121 °C (determined by DSC analysis), T_d^5 = 275 °C (determined by TGA analysis) and DSC. [f]Neat conditions. [g]Toluene (8.0 M).

3. Depolymerization procedures

3.1 General procedure for the TBD-initiated depolymerization of PLC into MO

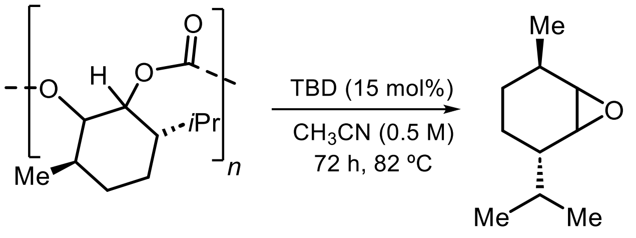

PMC **MO** *(dr = 3:2)*

In a nitrogen-filled glovebox, a 10 mL sealed tube was introduced containing **PMC** (80 mg, 0.40 mmol) and 0.80 mL of a solution of TBD in ACN (52.7 mg/5 mL). The reaction mixture was stirred under reflux (using an oil bath) for 72 hours. The mixture was then concentrated in vacuo and purified via column chromatography (cyclohexane:ethyl acetate, 95:5 v/v) obtaining the purified product **MO** (58 mg, 92% yield, *dr* = 7:3). **NB**. A scale up using 500 mg of **PMC** was performed following the same procedure, with a scaling factor of 10 (90% isolated yield, *dr* = 7:3).

3.2 General procedure for the TBD-initiated depolymerization of PLC into MC

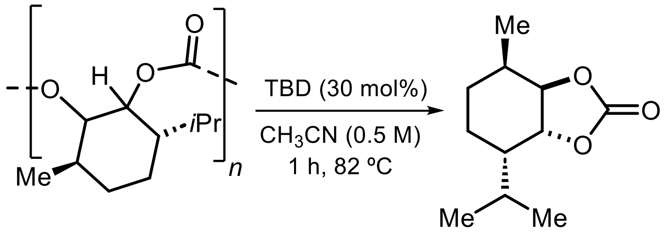

PMC **MC** *(trans, dr = 2:1)*

In a nitrogen-filled glovebox, a 10 mL sealed tube was introduced containing **PMC** (50 mg, 0.25 mmol) and 0.5 mL of a solution of TBD in ACN (42.1 mg/2 mL). The reaction mixture was stirred under reflux (oil bath) for 1 hour. The reaction mixture was then concentrated under vacuum, dissolved in a minimum amount of DCM and then slowly added to a solution of MeOH/HCl (12:1 v/v). The precipitated PMC (7.7 mg) was recovered by decantation. The decanted solution was concentrated under vacuum and the residue purified by column chromatography (cyclohexane:ethyl acetate (8:2 to 6:4 v/v) obtaining **MC** (23.8 mg, 78% isolated yield *brsm*, *dr* = 2:1) and the remaining **PMC** (9.2 mg, total recovered **PMC** yield *busm* = 90%). **NB**. A scale up at 500 mg of PMC was performed following the same procedure (scaling factor of 10) giving **MC** with a 72% isolated yield *brsm*, *dr* = 2:1, and the recovered **PMC** *busm* was 95%.

3.3 General procedure for the TBD-initiated depolymerization of PLC into MC

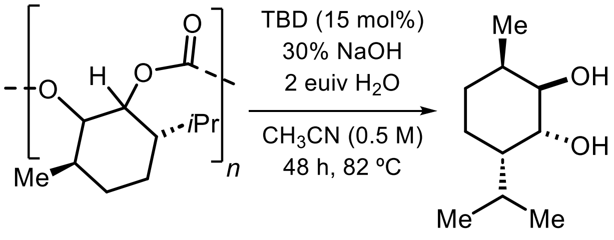

PMC **MD** *(dr = 4:1)*

In a 10 mL sealed tube were mixed **PMC** (50 mg, 0.25 mmol), H_2O (9.1 mg, 2 equiv), NaOH (3.0 mg, 0.3 equiv) and 0.50 mL of a solution of TBD in ACN (52.7 mg/5 mL). The reaction mixture was stirred under reflux (oil bath) for 48 hours. The reaction mixture was concentrated under vacuum, neutralized with HCl (1 M) and extracted with ethyl acetate. The crude product was then purified via column chromatography (cyclohexane:ethyl acetate, 9:1 to 7:3) obtaining the purified product **MD** (40.7 mg, 94% yield). **NB**. scale up at 500 mg of **PMC** was done following a previously reported procedure (94% isolated yield, *dr* = 4:1).[4]

3.4 Additional information on the depolymerization studies

Table S3. Depolymerization and a preliminary kinetic study.

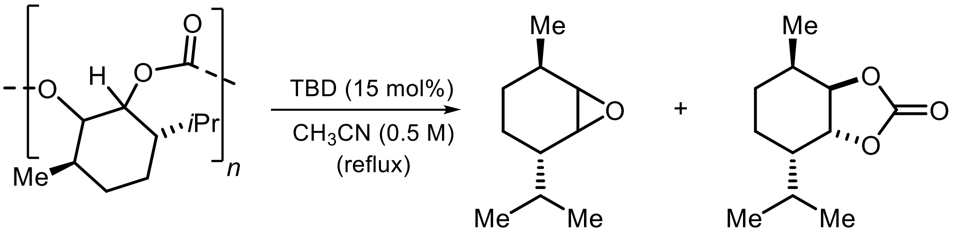

PMC **MO** **MC**

Entry[a]	Time (h)	PMC (%)[b]	MC (%)[b]	MO (%)[b]
1	6	40	47	13
3	24	23	36	41
4	48	6	29	65
5	72	0	21	79

[a]Reaction conditions: **PMC** (80 mg, 0,4 mmol). [b]Determined by ^{1}H NMR ($CDCl_3$).

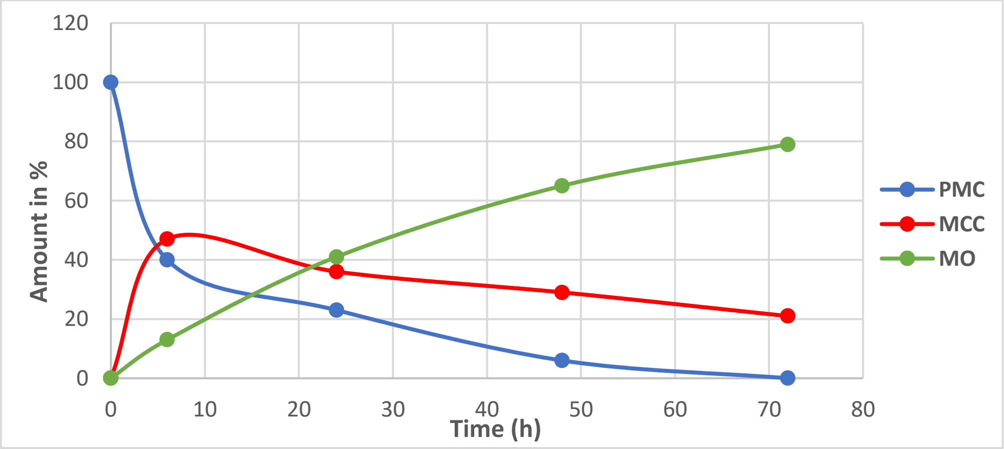

Table S4. Depolymerization study towards **MO** at prolonged reaction times.

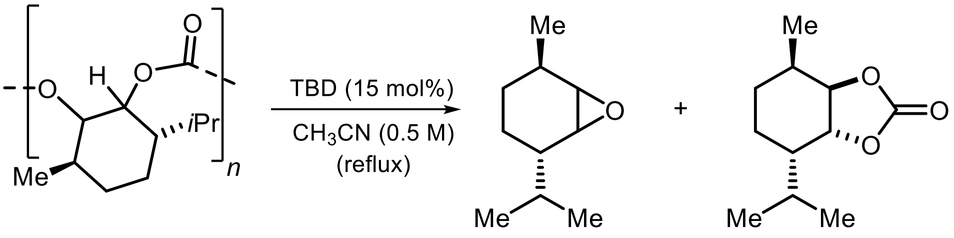

PMC **MO** **MC**

**Entry**[a]	**Time** (h)	**PMC** (%)[b]	**MC** (%)[b]	**MO** (%)[b]
1	24	0	16	84
2	48	0	13	87
3	72	0	3	97[c]

[a]Reaction conditions: **PMC** (80 mg, 0.40 mmol). [b]Determined by ^{1}H NMR ($CDCl_3$). [c]92% isolated yield (*dr* = 3:2), scale-up (500 mg): 90% isolated yield.

Table S5. Screening of lower temperatures towards selective formation of **MC**.

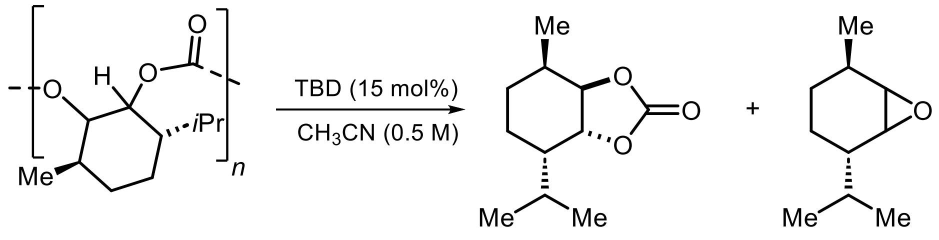

**Entry**[a]	**Time** (h)	**T** (ºC)	**PMC** (%)[b]	**MC** (%)[b]	**MO** (%)[b]
1	6	80	65	31	5
2	24	80	58	35	7
3	72	40	83	17	<1
4	144	40	74	19	7

[a]Reaction conditions: **PMC** (50 mg, 0.25 mmol). [b]Determined by ^{1}H NMR ($CDCl_3$).

Table S6. Screening of different (shorter) reaction times towards selective formation of MC.

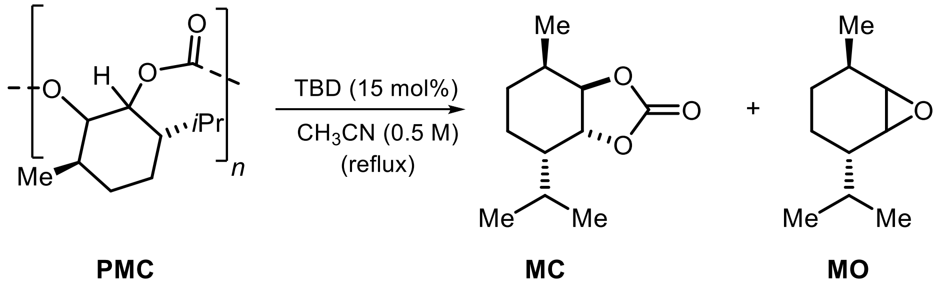

**Entry**[a]	**Time** (h)	**PMC** (%)[b]	**MC** (%)[b]	**MO** (%)[b]
1	0.5	63	34[c]	3
2	1	53	43[c]	4
3	2	47	47	6
4	3	43	48	9

[a]Reaction conditions: **PMC** (50 mg, 0.25 mmol). [b]Determined by ^{1}H NMR ($CDCl_3$). [c]Selectivity > 90%.

Table S7. Screening of lower TBD loadings towards selective formation of **MC**.

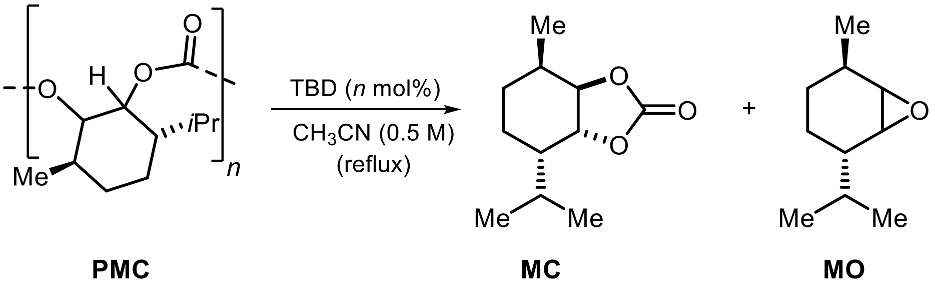

**Entry**[a]	**Time** (h)	**PMC** (%)[b]	**MC** (%)[b]	**MO** (%)[b]
1	1	62	36	2
2	2	63	34	2
3	3	60	35	5
4	24	61	16	23

[a]Reaction conditions: **PMC** (50 mg, 0.25 mmol). [b]Determined by ^{1}H NMR ($CDCl_3$).

Table S8. Screening of higher TBD loading and process conditions towards selective formation of **MC**.

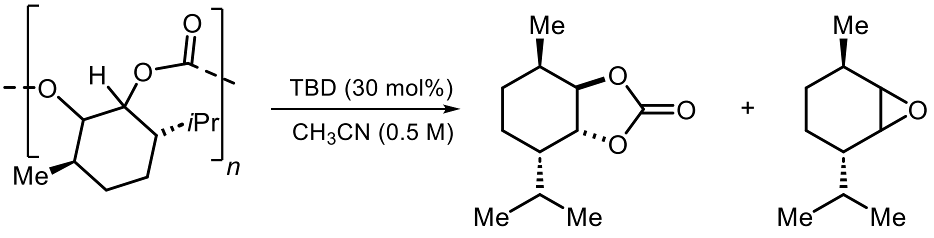

PMC **MC** **MO**

**Entry**[a]	**Time** (h)	**T** (ºC)	**PMC** (%)[b]	**MC** (%)[b]	**MO** (%)[b]
1	1	80	79	20	1
2	2	80	69	29	2
3	4	80	67	30	3
**4**	**1**	**110**	**38**	**56** [c]	**6**
5	2	110	33	57	10
6	4	110	31	52	17

[a]Reaction conditions: **PMC** (50 mg, 0.25 mmol). [b]Determined by ^{1}H NMR ($CDCl_3$). [c]Isolated yield *brsm* = 78%, *dr* = 2:1.

Table S9. Screening of other (organo)catalysts for the depolymerization of **PMC**.

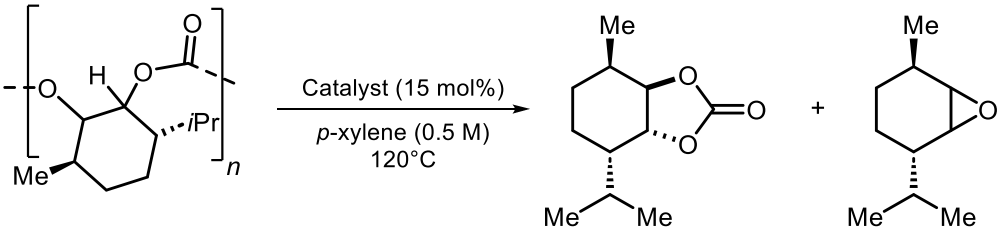

**Entry**[a]	**Catalyst**	**Time** (h)	**PMC** (%)[b]	**MC** (%)[b]	**MO** (%)[b]
1	TBD	24	19	29	52
		72	0	13	86
2	DBU	24	94	3	3
		72	55	3	42
3	KHMDS	24	62	7	31
		72	40	13	47
4	*n*-Bu_4NN_3	24	99	0	0
		72	97	1	2

[a]Reaction conditions: **PMC** (50 mg, 0.25 mmol). [b]Determined by ^1H NMR ($CDCl_3$).

Table S10. Study of the influence of different reaction conditions towards a selective formation of **MD** catalyzed by TBD in the presence of H_2O.

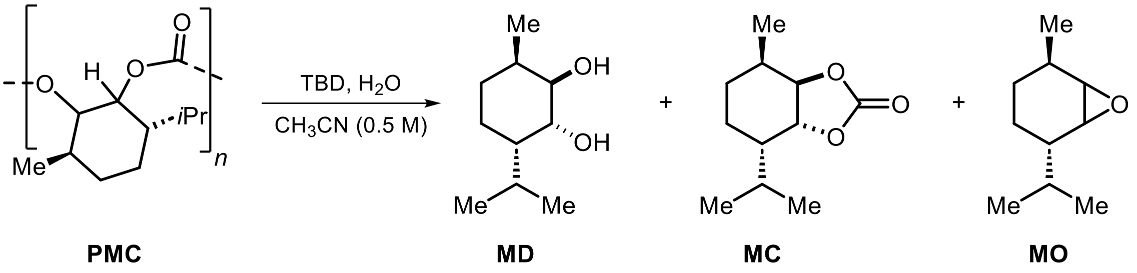

Entry[a]	Time (h)	T (ºC)	TBD (mol%)	H₂0 (equiv)	PMC (%)[b]	MD (%)[b]	MC (%)[b]	MO (%)[b]
1	24	80	15	1	67	25	5	3
2	72	80	15	1	48	43	4	5
3	24	80	10	2	79	19	2	0
4	72	80	10	2	62	37	1	0
5	72	110	10	2	14	79	0	7
6	24	110	15	2	5	86	0	9
7	48	110	15	2	0	91[c]	0	9
8[d]	48	110	15	2	0	>95[e]	0	<5
9	72	110	15	2	0	90	0	10
10	24	110	15	10	37	55[f]	0	8

[a]Reaction conditions: **PMC** (50 mg, 0.25 mmol). [b]Determined by ^1H NMR ($CDCl_3$). [c]Isolated yield = 86%, *dr* = 4:1. [d]Reaction carried out using NaOH (30 mol%). [e]Isolated yield was 94%, *dr* = 4:1.

Table S10. Study of the influence of the reaction conditions on the selective formation of **MD** catalyzed by TBD in MeOH.

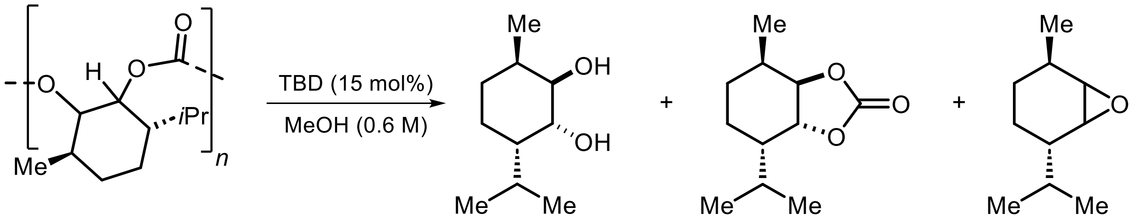

PMC **MD** **MC** **MO**

**Entry**[a]	**T** (ºC)	**Time** (h)	**PMC** (%)[b]	**MD** (%)[b]	**MC** (%)[b]	**MO** (%)[b]
1	110	6	6	72	0	22
2	110	24	0	74	0	26
3	80	6	40	11	0	49
4	80	24	16	68	0	16
5[c]	80	24	7	74	0	17

[a]Reaction conditions: **PMC** (50 mg, 0.25 mmol). [b]Determined by ^{1}H NMR ($CDCl_3$). [c]Reaction carried out with NaOH (30 mol%).

Control experiments:

PMC (80 mg, 0.4 mmol) in dry ACN (0.50 M) at 110 °C, without TBD:

No observable depolymerization was noticed after 24 h. The polymer did not dissolve in ACN even at high temperatures, and a pellet was formed.

MC (80 mg, 0.4 mmol) in dry ACN (0.50 M) at 110 °C, without TBD:

After 72 h, no reaction was observed by ^{1}H NMR, while the MC does not decompose and no signs of epoxide or diol were noted.

4. Diversification studies using MO as a precursor

** For each product, only the major isomer is shown **

Synthesis of chlorohydrin 1

1

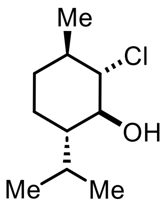

2-Menthene-oxide (1.08 g, 7.00 mmol), DCM (70 mL) and HCl (36% aqueous, 21 mL) were stirred together for 4 h at room temperature. Then, the mixture was washed with saturated $NaHCO_3$, brine and dried over Na_2SO_4. The organic solvent was evaporated under reduced pressure and the residue was purified via column chromatography (hexane:ethyl acetate, 9:1 v/v), obtaining the purified product as a yellowish oil (1.28 g, 96% yield, *rr* = 7:3). **^{1}H NMR** (400 MHz, $CDCl_3$) δ 4.33 – 4.28 (m, major), 4.16 – 4.07 (m, minor), 3.85 (s, major), 2.20 – 2.05 (m, 1H), 1.70 – 1.59 (m, 2H), 1.55 – 1.34 (m, 3H), 1.30 – 1.24 (m, 2H), 1.03 – 0.91 (m, 9H). **^{13}C NMR** (101 MHz, $CDCl_3$) δ 74.8, 64.0, 42.1, 29.5, 29.2, 27.7, 23.8, 20.8, 20.1, 17.7. **HRMS** (ESI/TOF) *m/z*: $[M - H]^-$ Calcd. for $C_{10}H_{18}ClO$: 189.1041; found 189.1038. **FT-IR** (neat) ν (cm^{-1}) 3416.

Synthesis of alkynylated compound 2

2

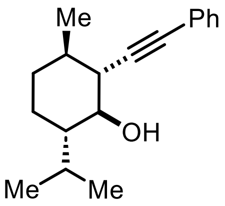

A solution of phenyl acetylene (248 mg, 2.43 mmol, 1.5 equiv) in anhydrous THF (50 mL) was cooled to −78 °C under a nitrogen atmosphere. To this solution, *n*-butyllithium (2.5 M in hexanes, 0.972 mL, 7.5 mmol, 1.5 equiv) was added dropwise for 30 minutes at −78 °C. Menthene oxide (250 mg, 1.62 mmol) was then added to the reaction mixture, followed by boron trifluoride diethyl etherate (35 mg, 2.43 mmol, 1.5 equiv), maintaining the temperature at −78 °C. The reaction mixture was stirred at this temperature for an additional 4 h. Upon completion, the reaction was quenched by the careful addition of saturated aqueous ammonium chloride solution. The resulting mixture was extracted with ethyl acetate (3 × 20 mL), and the combined organic layers were washed with saturated brine, dried over anhydrous sodium sulfate, filtered, and concentrated under reduced pressure. The crude product was purified by flash column chromatography on silica gel (hexane /ethyl acetate (4:1 v/v) to yield the desired product as a colorless oil (356 mg, 86% yield, *rr* = 2:1, *dr* = 5:1). **^{1}H NMR** (400 MHz, $CDCl_3$) δ 7.46 – 7.36 (m, 2H), 7.34 – 7.23 (m, 3H), 4.34 – 4.29 (m, minor), 4.24 – 4.20 (m, minor), 3.96 – 3.91 (m, major), 3.87 – 3.82 (m, minor), 3.20 – 3.13 (m, major), 2.95 – 2.90 (m, minor), 2.15 – 1.97 (m, 1H), 1.80 – 1.27 (m, 6H), 1.10 – 0.93 (m, 9H). **^{13}C NMR** (101 MHz, $CDCl_3$) δ 131.7, 128.3, 128.3, 127.8, 124.1, 124.0, 89.1, 88.8, 84.9, 84.5, 77.5, 77.4, 77.2, 76.8, 74.8, 74.3, 71.1, 64.2, 43.3, 42.4, 42.2, 40.5, 38.7, 38.3, 32.1, 31.8, 31.6, 31.4, 30.7, 30.3, 30.2, 29.8, 29.8, 29.8, 29.6, 29.5, 29.3, 29.2, 28.5, 28.1, 27.8, 26.2, 24.2, 23.9, 22.8, 21.2, 21.1, 21.0, 20.9, 20.8, 20.2, 20.0, 18.3, 17.8, 14.3. **HRMS** (ESI/TOF) *m/z*: $[M + H]^+$ Calcd. for $C_{18}H_{25}O$: 257.1900; found 257.1891. **FT-IR** (neat) ν (cm^{-1}) 3426, 754, 690.

Synthesis of 1,2-azido-alcohol 3

3

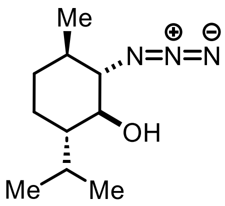

2-Menthene-oxide (155 mg, 1.00 mmol), ammonium chloride (108 mg, 2.01 mmol) and NaN_3 (523 mg, 8.04 mmol) were mixed together in a MeOH and water solution (0.30 M, 8:1 v/v). The reaction mixture was stirred for 18 h at room temperature. The solvent was evaporated, water was added and the mixture was extracted with ethyl acetate (4 × 25 mL). The organic layers were combined, washed with brine and dried over Na_2SO_4. The solvent was removed under reduced pressure and the residue was purified via column chromatography (hexane: ethyl acetate, 95:5 v/v), obtaining the purified product as a colorless oil (141 mg, 71% yield, *rr* = 7:3). **^{1}H NMR** (400 MHz, $CDCl_3$) δ 4.10 (s, minor), 3.93 – 3.79 (m, major),

3.62 – 3.54 (m, minor), 2.04 – 1.77 (m, 1H), 1.67 – 1.48 (m, 3H), 1.45 – 1.12 (m, 4H), 1.01 – 0.90 (m, 9H). **^{13}C NMR** (101 MHz, $CDCl_3$) δ 72.5, 64.5, 41.1, 30.6, 29.1, 27.5, 24.3, 20.9, 20.7, 17.6. **HRMS** (ESI/TOF) *m/z*: $[M + H – N_2]^+$ Calcd. for $C_{10}H_{20}NO$: 170.1539; found 170.1545. **FT-IR** (neat) ν (cm^{-1}) 3427, 2093.

Synthesis of 1,2-amino-alcohol 4

4

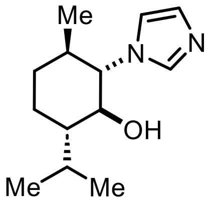

2-Menthene-oxide (100 mg, 648 µmol) and imidazole (110 mg, 1.62 mol) were stirred in a vial at 70 °C for 72 h. The mixture was then purified via preparative TLC (DCM:MeOH, 95:5 v/v) obtaining the product as a white solid in 78% yield (*rr* = 2:1). **^{1}H NMR** (400 MHz, $CDCl_3$) δ 7.65 – 7.53 (m, 1H), 7.13 – 6.97 (m, 2H), 4.49 (t, *J* = 3.8 Hz, 1H, major), 4.24 – 4.22 (m, 1H, minor), 4.04 – 4.02 (m, 1H, minor), 3.73 – 3.69 (m, 1H, major), 2.30 (dd, *J* = 12.6, 6.9 Hz, 1H), 1.93 – 1.84 (m, 1H), 1.79 – 1.70 (m, 1H), 1.64 – 1.43 (m, 3H), 1.32 – 1.23 (m, 2H), 0.98 – 0.91 (m, 3H), 0.85 – 0.72 (m, 6H). **^{13}C NMR** (101 MHz, $CDCl_3$) δ 138.2, 128.7, 120.3, 74.5, 60.8, 40.3, 30.6, 28.8, 26.8, 25.6, 21.1, 20.7, 17.8. **HRMS** (ESI/TOF) *m/z*: $[M + H]^+$ Calcd. for $C_{13}H_{23}N_2O$: 223.1805; found 223.1810. **FT-IR** (neat) *ν* (cm^{-1}) 3175, 2956, 2928, 2871.

Synthesis of alcohol 5

5

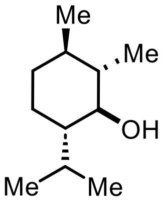

To a stirred solution of menthene oxide (250 mg, 1.62 mmol) in dry toluene (5 mL) under a nitrogen atmosphere, triphenylantimony (24 mg, 0.081 mmol, 0.05 equiv) was added. Subsequently, trimethylaluminum (116 mg, 1.62 mmol, 2.0 M solution in toluene) was added dropwise via a syringe. The reaction mixture was stirred at room temperature for 32 h. After completion, the reaction was carefully quenched with 5 mL of 1 N aqueous HCl. The organic and aqueous layers were separated, and the aqueous phase was extracted twice with diethyl ether (10 mL). The combined organic extracts were dried over anhydrous sodium sulfate, filtered, and concentrated under reduced pressure. The crude residue was purified by flash column chromatography on silica gel using hexanes/ethyl acetate (99:1, v/v) as the eluent to afford the desired product as a colorless oil (205 mg, 74% yield, *rr* = 7:3). **^{1}H NMR** (400 MHz, $CDCl_3$) δ 3.84 (s, minor), 3.54 (s, major), 2.17 – 1.90 (m, 1H), 1.88 – 1.61 (m, 2H), 1.59 – 1.24 (m, 5H), 1.16 – 1.04 (m, 1H), 1.00 – 0.78 (m, 12H). **^{13}C NMR** (101 MHz, $CDCl_3$) δ 77.11, 40.40, 37.04, 30.19, 29.27, 28.44, 24.04, 21.45, 20.56, 18.30, 11.12. **HRMS** (ESI/TOF) *m/z*: $[M + Na]^+$ Calcd. for $C_{11}H_{22}NaO$: 193.1569; found 193.1563. **FT-IR** (neat) *ν* (cm^{-1}) 3403.

Synthesis of oligoester 6

6

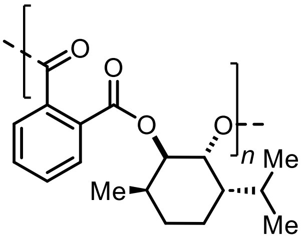

In a nitrogen-filled glovebox, 2-menthene oxide **MO** (200 mg, 1.30 mmol), phthalic anhydride (96 mg, 0,65 mmol) and a binary catalyst (1.0 mol% of **Fe^{Me}** complex, analogue of **Al^{Me}**, and PPNCl as initiator, 0.50 mol%)[5] were placed in an oven-dried 4 mL vial and the vial was sealed with a Teflon-lined cap. The reaction mixture was then removed from the glovebox and placed in oil bath at 25 °C for 48 h. Then, the volatiles were removed under vacuum. The crude product was dissolved in a minimal amount of DCM and precipitated with a solution of HCl (1 M) in methanol. Finally, the polymer was washed with methanol and dried under vacuum obtaining a white powder (159 mg, 81% yield). **^{1}H NMR** (400 MHz, $CDCl_3$) δ 8.11 – 7.29 (m, 4H), 5.74 – 4.81 (m, 2H), 2.16 – 0.75 (m, 16H). **^{13}C NMR** (101 MHz, $CDCl_3$) δ 166.5, 166.2, 165.6, 132.6, 132.0, 131.2, 131.0, 130.5, 129.0, 85.4, 80.8, 77.5, 77.4, 77.16, 76.8, 76.5, 73.7, 73.6, 73.4, 73.2, 71.6, 71.3, 71.0, 47.8, 47.8, 47.1, 42.2, 41.8, 37.1, 36.1, 36.0, 32.5, 32.2, 32.0, 30.7, 30.6, 30.4, 29.0, 28.6, 28.5, 26.4,

25.7, 25.6, 25.5, 24.9, 24.5, 23.8, 22.7, 22.3, 21.1, 21.1, 21.0, 21.0, 20.9, 20.9, 18.4, 18.2, 18.1, 17.7, 17.6, 17.6, 16.2, 16.1, 16.0, 16.0, 15.9.

5. Diversification studies using MC as synthon

Synthesis of thiocarbonate 7

7 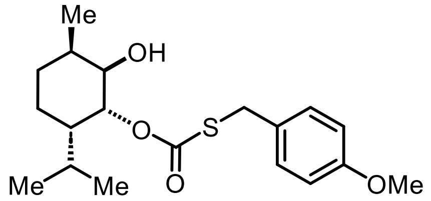

Menthene cyclic-carbonate (104 mg, 514 µmol), 4-methoxyphenyl methanethiol (150 mg, 976 µmol) and TBD (14 mg, 101 µmol) were mixed in a vial under Argon. The reaction mixture was stirred for 18 h at 40 °C. The crude product was purified via column chromatography (hexane:ethyl acetate, 95:5 to 8:2 v/v) obtaining the product as a colorless oil (142 mg, 78% yield, *rr* = 1:1). **^{1}H NMR** (400 MHz, $CDCl_3$) δ 7.28 – 7.22 (m, 2H), 6.87 – 6.81 (m, 2H), 5.21 – 5.17 (m, 0.5H), 5.00 – 4.95 (m, 0.5H), 4.07 (s, 2H), 4.04 – 4.00 (m, 0.5H), 3.81 – 3.74 (m, 3H + 0.5H), 2.05 – 1.95 (m, 0.5H), 1.71 – 1.42 (m, 5H + 0.5H), 1.33 – 1.23 (m, 2H), 0.97 – 0.87 (m, 9H). **^{13}C NMR** (101 MHz, $CDCl_3$) δ 170.7, 170.5, 159.0, 130.0, 129.9, 129.0, 128.9, 114.1, 79.5, 70.8, 68.1, 55.3, 42.4, 40.6, 35.0, 35.0, 30.8, 29.4, 28.7, 28.6, 28.4, 27.3, 24.3, 23.4, 21.1, 20.9, 20.8, 20.6, 17.4. **HRMS** (ESI/TOF) *m/z*: $[M + Na]^+$ Calcd. for $C_{19}H_{28}NaO_4S$: 375.1601; found 375.1615. **FT-IR** (neat) ν (cm^{-1}) 3506, 1704.

Synthesis linear of carbonate 8

8 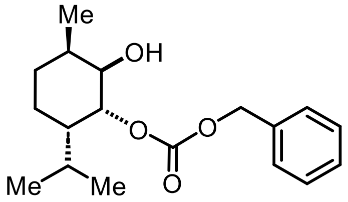

In a nitrogen-filled glove-box, menthene cyclic-carbonate (100 mg, 504 µmol), BnOH (60 mg, 555 µmol) and TBD (14 mg, 101 µmol) were mixed in a vial. The reaction mixture was stirred for 18 h at 40 °C. The crude product was purified via column chromatography (hexane:ethyl acetate, 9:1 v/v) obtaining the purified product as a colorless oil (111 mg, 72% yield, *rr* = 1:1). **^{1}H NMR** (400 MHz, $CDCl_3$) δ 7.43 – 7.29 (m, 5H), 5.20 – 5.13 (m, 2H), 4.95 – 4.92 (m, 0.5H), 4.73 – 4.69 (m, 0.5H), 4.04 – 4.00 (m, 0.5H), 3.81 – 3.72 (m, 0.5H), 2.07 – 1.96 (m, 0.5H), 1.78 – 1.75 (m, 0.5H), 1.69 – 1.61 (m, 1H), 1.60 – 1.40 (m, 3H), 1.37 – 1.24 (m, 3H), 0.95 – 0.89 (m, 9H). **^{13}C NMR** (101 MHz, $CDCl_3$) δ 155.2, 155.0, 135.4, 135.4, 128.6, 128.6, 128.5, 128.5, 128.2, 80.1, 70.8, 69.6, 69.6, 68.0, 42.3, 40.6, 30.7, 29.2, 28.6, 28.6, 28.4, 27.3, 24.2, 23.4, 21.1, 20.9, 20.8, 20.6, 17.4, 17.2. **HRMS** (ESI/TOF) *m/z*: $[M + Na]^+$ Calcd for $C_{18}H_{26}NaO_4$: 329.1723; found 329.1733. **FT-IR** (neat) ν (cm^{-1}) 3490, 1740.

Synthesis of carbamate 9

9 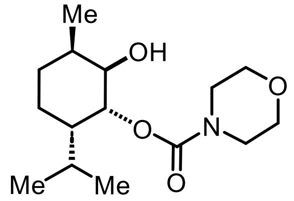

Into a glove-box, menthene cyclic-carbonate (102 mg, 514 µmol), morpholine (56 mg, 640 µmol) and TBD (14 mg, 101 µmol) were mixed in a vial. The reaction mixture was stirred for 18 h at 40 °C. The crude product was purified via column chromatography (hexane:ethyl acetate, 9:1 to 8:2 v/v) obtaining the product as a colorless oil (144 mg, 98% yield, *rr* = 1:1). **^{1}H NMR** (500 MHz, $CDCl_3$) δ 5.00 – 4.95 (m, 0.5H), 4.76 (t, *J* = 3 Hz, 0.5H), 4.01 – 3.97 (m, 0.5H), 3.77 – 3.71 (m, 0.5H), 3.71 – 3.57 (m, 4H), 3.53 – 3.38 (m, 4H), 2.11 – 2.00 (m, 1H), 1.77 (s, 1H), 1.71 – 1.57 (m, 2H), 1.50 – 1.17 (m, 4H), 0.96 – 0.87 (m, 9H). **^{13}C NMR** (126 MHz, $CDCl_3$) δ 155.26, 154.93, 74.55, 70.89, 68.11, 66.64, 42.88, 40.55, 31.00, 29.23, 29.11, 29.06, 28.62, 27.53, 24.93, 23.56, 21.18, 20.97, 20.78, 17.57. **HRMS** (ESI/TOF) *m/z*: $[M + Na]^+$ Calcd. for $C_{15}H_{27}NNaO_4$: 308.1832; found 308.1846. **FT-IR** (neat) ν (cm^{-1})3454, 1676.

Synthesis of bis-borate ester 10

10

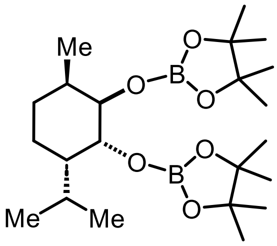

Menthene cyclic-carbonate (198 mg, 1 mmol) and HBpin (397 mg, 3.1 mmol) were dissolved in dry toluene (1.2 mL) in an oven-dried flask under argon. Then, di-*n*-butyl-magnesium (1 M in heptane, 4.2 mg, 30 µmol) was added and the reaction mixture was stirred at 70 °C for 18 h. The solvent was removed under reduced pressure obtaining the product as a colorless viscous oil (91% ^{1}H-NMR yield using mesitylene as internal standard). **^{1}H NMR** (400 MHz, $CDCl_3$) δ 4.39 – 4.29 (m, 1H), 4.09 – 3.99 (m, 1H), 2.02 – 1.84 (m, 1H), 1.56 – 1.51 (m, 1H), 1.35 – 1.26 (m, 29H), 1.01 – 0.82 (m, 9H). **^{13}C NMR** (101 MHz, $CDCl_3$) δ 83.3, 82.8, 82.7, 76.3, 74.1, 73.9, 73.4, 70.7, 41.7, 41.6, 41.5, 30.2, 30.1, 30.0, 28.8, 28.5, 28.5, 28.0, 27.9, 27.8, 25.0, 24.9, 24.8, 24.7, 24.7, 24.6, 24.6, 24.6, 24.6, 23.8, 23.7, 23.6, 21.3, 21.2, 21.1, 20.9, 20.7, 17.7, 17.6, 17.6. **FT-IR** (neat) ν (cm^{-1}) 2976, 2931, 2872, 1499, 1424. *Note that the product cannot be columned due to its instable character, see reference [6] for further details*.

6. Diversification studies using MD as a precursor

Synthesis of bis-aldehyde 12

12 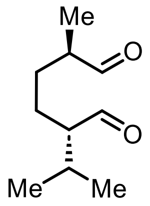

$NaIO_4$ (186 mg, 871 µmol), silica (0.50 g), water (2.3 mL) and DCM (4 mL) were mixed in a vial. Under vigorous stirring, a solution of *trans*-menthene-diol (100 mg, 580 µmol) in DCM (3 mL) was added dropwise. The reaction mixture was stirred for 18 h at 40 ºC, then filtered (*the silica was rinsed with DCM*) and the filtrate was dried over Na_2SO_4. The solvent was removed under reduced pressure obtaining the product as a colorless oil (90 mg, 91% yield). **^{1}H NMR** (400 MHz, $CDCl_3$) δ 9.67 – 9.56 (m, 2H), 2.41 – 2.28 (m, 1H), 2.11 – 1.97 (m, 2H), 1.75 – 1.62 (m, 2H), 1.51 – 1.40 (m, 1H), 1.32 – 1.19 (m, 1H), 1.11 (d, *J* = 7.1 Hz, 3H), 0.99 – 0.93 (m, 6H). **^{13}C NMR** (101 MHz, $CDCl_3$) δ 205.3, 204.6, 58.3, 46.4, 28.5, 28.3, 23.1, 20.2, 19.7, 13.4. **HRMS** (ESI/TOF) *m/z*: $[M + H]^+$ Calcd. for $C_{10}H_{19}O_2$: 171.1380; found 171.1375. **FT-IR** (neat) ν (cm^{-1}) 1719.

Synthesis of bis-allyl ether 13

13 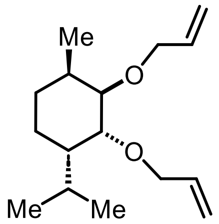

Trans-menthene-diol (550 mg, 3.19 mmol) and allyl bromide (1.93 g, 16 mmol) were stirred in dry DMF (11 ml) at 0 °C under Ar. Then sodium hydride (639 mg (60% wt dispersion in mineral oil, 16 mmol) was added in small portions. The reaction mixture was stirred for 18 h at room temperature. Water was carefully added and the product isolated by extraction using diethyl ether. The solvent was removed under reduced pressure and the residue was purified via column chromatography (hexane:ethyl acetate, 99:1 v/v), obtaining the product as a colorless oil (788 mg, 98% yield). **^{1}H NMR** (400 MHz, $CDCl_3$) δ 5.99 – 5.82 (m, 2H), 5.30 – 5.22 (m, 2H), 5.19 – 5.09 (m, 2H), 4.10 – 3.89 (m, 4H), 3.64 – 3.59 (m, 1H), 3.38 (t, *J* = 3.2 Hz, 1H), 1.88 – 1.76 (m, 1H), 1.70 – 1.59 (m, 1H), 1.56 – 1.53 (m, 1H), 1.37 – 1.23 (m, 4H), 0.95 – 0.88 (m, 9H). **^{13}C NMR** (101 MHz, $CDCl_3$) δ 135.8, 135.7, 116.3, 116.1, 78.1, 75.9, 71.8, 70.8, 42.3, 30.6, 28.6, 26.9, 24.1, 21.1, 21.0, 18.0. **HRMS** (ESI/TOF) *m/z*: $[M + Na]^+$ Calcd. for $C_{16}H_{28}NaO_2$: 275.1982; found 275.1988. **FT-IR** (neat) ν (cm^{-1}) 3080, 1647.

Synthesis of cyclic sulfite 14

14 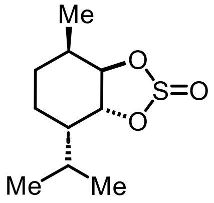

To a stirred solution of the menthene diol (410 mg, 2.38 mmol) in DCM (7 mL) under argon was added triethylamine (530 mg, 5.24 mmol), followed by dropwise addition of thionyl chloride (340 mg, 2.86 mmol) in DCM (3 mL) at 0 °C. The reaction mixture was stirred at room temperature for 5 h. Then water was added, the mixture was filtered and washed with water and extracted with DCM. The combined organic phases were washed with HCl (1 M), NH_4Cl (saturated solution) and dried over Na_2SO_4 The solvent was removed under reduced pressure and the residue was purified via column chromatography (hexane:ethyl acetate, 95:5 v/v), obtaining the product as a colorless oil (476 mg, 92% yield). **^{1}H NMR** (400 MHz, $CDCl_3$) δ 4.74 – 4.66 (m, 1H), 4.25 – 4.15 (m, 1H), 2.72 – 2.51 (m, 1H), 2.15 – 1.41 (m, 5H), 1.37 – 1.20 (m, 1H), 1.16 – 0.98 (m, 6H), 0.97 – 0.90 (m, 3H). **^{13}C NMR** (101 MHz, $CDCl_3$) δ 84.01, 83.52, 77.69, 44.58, 44.03, 31.39, 30.93, 30.82, 26.03, 25.55, 25.17, 24.98, 23.17, 22.99, 22.47, 22.09, 21.85, 21.70, 11.09, 11.06. **HRMS** (ESI/TOF) *m/z*: $[M + Na]^+$ Calcd. for $C_{10}H_{18}NaO_3S$: 241.0869; found 241.0876. **FT-IR** (neat) ν (cm^{-1}) 1461, 1208, 1006, 756.

Synthesis of cyclic sulfate 15

15 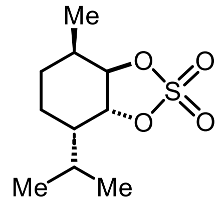

To a stirred solution of menthene-cyclic sulfite **14** (144 mg, 660 µmol) in ACN (1 mL) at 0 °C was added $NaIO_4$ (705 mg, 3.30 mmol), $RuCl_3$ (13.7 mg, 66 µmol) and cold water (1 mL). The reaction mixture was stirred at room temperature for 2 h. The product was extracted with ethyl acetate and dried over Na_2SO_4. The solvent was removed under reduced pressure and the residue was purified via column chromatography (hexane:ethyl acetate, 95:5 v/v), obtaining the product as a colorless oil (133 mg, 86% yield). **^{1}H NMR** (400 MHz, $CDCl_3$) δ 5.05 – 4.94 (m, 2H), 2.64 – 2.54 (m, 1H), 2.08 – 1.62 (m, 5H), 1.54 – 1.49 (m, 1H), 1.13 – 1.07 (m, 6H), 0.99 – 0.94 (m, 3H). **^{13}C NMR** (101 MHz, $CDCl_3$) δ 85.6, 85.1, 43.7, 30.6, 25.2, 25.1, 22.5, 21.7, 21.7, 10.8. **HRMS** (ESI/TOF) *m/z*: $[M + Na]^+$ Calcd. for $C_{10}H_{18}NaO_4S$: 257.0818; found 257.0811. **FT-IR** (neat) ν (cm^{-1}) 1726, 1461, 1380, 1206, 965, 782.

Synthesis of silyl ether 16

16 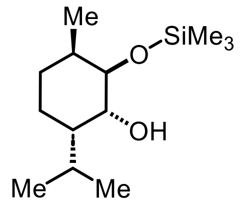

To a stirred solution of *trans*-menthene-1,2-diol (150 mg, 0.872 mmol) and imidazole (192 mg, 2.83 mmol, 3.25 equiv) in anhydrous diethyl ether (5 mL), cooled to 0 °C, trimethylsilyl chloride (TMSCl) (0.23 mL, 1.87 mmol, 2.15 equiv) was added dropwise over 30 minutes under an inert atmosphere (N_2). The reaction mixture was then allowed to warm to room temperature and stirred for 16 h. The reaction progress was monitored by thin-layer chromatography (TLC). Upon completion, the reaction was quenched by the addition of water (5 mL), and the organic layer was separated. The aqueous phase was extracted with ethyl acetate (5 mL), and the combined organic layers were dried over anhydrous sodium sulfate (Na_2SO_4), filtered, and concentrated in vacuo. The crude product was purified by silica gel column chromatography using hexane/ethyl acetate (99:1, v/v) as the eluent to afford the desired product as a colorless oil (128 mg, 60% yield, *rr* = 2:1). **^{1}H NMR** (400 MHz, $CDCl_3$) δ 4.02 – 3.77 (m, 1H), 3.73 – 3.46 (m, 1H), 2.00 – 1.70 (m, 1H), 1.64 – 1.48 (m, 2H), 1.43 – 1.25 (m, 4H), 1.23 – 1.14 (m, 1H), 0.98 – 0.85 (m, 9H), 0.13 – 0.11 (m, 9H). **13C NMR** (101 MHz, $CDCl_3$) δ 74.7, 71.6, 41.4, 30.4, 28.7, 27.7, 23.8, 21.2, 20.6, 18.0, 0.5. **HRMS** (ESI/TOF) *m/z*: $[M + Na]^+$ Calcd. for $C_{13}H_{28}NaO_2Si$: 267.1759; found 267.1751. **FT-IR** (neat) ν (cm^{-1}) 3414, 1251, 836.

Synthesis of mono-ketone 17

17 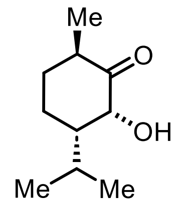

To a stirred solution of *trans*-menthene-1,2-diol (100 mg, 0.581 mmol) and pyridine (91 mg, 1.16 mmol, 2.0 equiv) in dry dichloromethane (DCM, 3 mL) at 0 °C under an argon atmosphere, pyridinium chlorochromate (PCC, 250 mg, 1.16 mmol, 2.0 equiv) was added portion-wise. The reaction mixture was allowed to warm to room temperature and stirred for 24 h. The mixture was diluted with DCM (10 mL) and washed sequentially with 1.5 N HCl (2 × 10 mL) followed by brine (10 mL). The organic layer was separated, dried over anhydrous sodium sulfate (Na_2SO_4), filtered, and concentrated under reduced pressure. The crude residue was purified by column chromatography on silica gel using hexane/ethyl acetate (99:1, v/v) as the eluent to afford the desired oxidized product as a colorless oil (89 mg, 90% yield, *rr* = 7:3). **^{1}H NMR** (400 MHz, $CDCl_3$) δ 4.21 (d, *J* = 3.0 Hz, 1H), 3.12 – 2.91 (m, 1H), 2.55 – 2.20 (m, 1H), 2.16 – 1.99 (m, 1H), 1.82 – 1.76 (m, 2H), 1.45 – 1.34 (m, 1H), 1.33 – 1.19 (m, 2H), 1.07 – 1.02 (m, 3H), 1.00 – 0.94 (m, 6H). **^{13}C NMR** (101 MHz, $CDCl_3$) δ 214.98, 76.31, 51.05, 39.95, 34.46, 28.33, 22.94, 21.01, 20.71, 14.30. **HRMS** (ESI/TOF) *m/z*: $[M + Na]^+$ Calcd. for $C_{10}H_{18}NaO_2$: 193.1569; found 193.1563. **FT-IR** (neat) ν (cm^{-1}) 1702.

Synthesis of bis-O-acetyl derivative 18

18

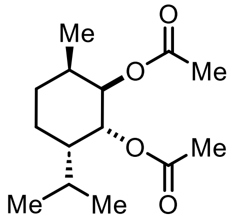

Trans-menthene-1,2-diol (150 mg, 0.872 mmol) and DMAP (10 mg, 0.087 mmol, 0.1 equiv) were dissolved in dry pyridine (2.0 mL), cooled to 0 °C under an inert atmosphere (N_2), and acetic anhydride (177 mg, 1.74 mmol, 2.0 equiv) was added dropwise. The reaction mixture was then allowed to warm to room temperature and stirred for 16 h. Progress of the reaction was monitored by ^{1}H NMR. Upon completion, the reaction was quenched by slow addition of water (5 mL), followed by extraction with ethyl acetate (2 × 10 mL). The organic layer was separated, and washed with 1.5 N HCl (1 × 5 mL), and then brine (1 × 5 mL). The organic layer was dried over anhydrous sodium sulfate (Na_2SO_4), filtered, and concentrated under reduced pressure. The crude residue was purified by silica gel column chromatography using hexanes/ethyl acetate (99:1, v/v) as the eluent to afford the diacetate product as a colorless oil (164 mg, 73% yield, *dr* = 13:1). **^{1}H NMR** (400 MHz, $CDCl_3$) δ 5.16 – 5.05 (m, major), 4.99 – 4.93 (m, major), 4.90 – 4.84 (m, minor), 4.68 – 4.63 (m, minor), 2.09 (s, 6H), 1.94 – 1.77 (m, 1H), 1.72 – 1.69 (m, 1H), 1.57 – 1.41 (m, 2H), 1.37 – 1.23 (m, 3H), 0.94 – 0.90 (m, 3H), 0.90 – 0.83 (m, 6H). **^{13}C NMR** (101 MHz, $CDCl_3$) δ 169.89, 169.73, 71.93, 70.18, 41.50, 30.08, 28.62, 28.45, 24.15, 21.02, 20.93, 20.84, 20.77, 17.33. **HRMS** (ESI/TOF) *m/z*: $[M + Na]^+$ Calcd. for $C_{14}H_{24}NaO_4$: 279.1577; found 279.1567. **FT-IR** (neat) ν (cm^{-1}) 1734, 1215.

7. Synthesis of new types of (bi)functional monomers

Synthesis of bis-thioether 19

19

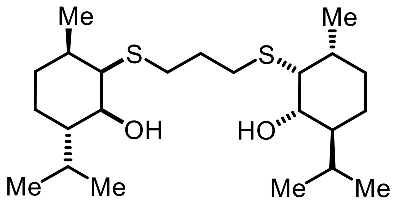

2-Chloro-6-isopropyl-3-methylcyclohexan-1-ol (250 mg, 1,31 mmol) and 1,3-propanedithiol (71 mg, 655 µmol) were dissolved in ethanol (2.5 mL) and cooled with an ice bath while a solution of NaOH in H_2O (8 M, 170 µL) was slowly added. The reaction mixture was stirred at room temperature for 18 h. The formed NaCl was filtrated off and the solvent evaporated under reduced pressure. Water was added to the residue and the product was extracted using chloroform. The combined organic phases were treated with brine, dried on Na_2SO_4 and the solvent evaporated. The product was purified via column chromatography (hexane:ethyl acetate, 8:2 v/v), obtaining the product as a viscous, colorless oil (227 mg, 83% yield). **^{1}H NMR** (400 MHz, $CDCl_3$) δ 4.14 (s, minor), 3.82 (s, major), 3.07 – 3.00 (m, major), 2.87 – 2.75 (m, 3H), 2.75 – 2.59 (m, 3H + minor), 2.25 – 2.12 (m, 2H), 2.07 – 1.95 (m, 2H), 1.93 – 1.85 (m, 1H), 1.79 – 1.65 (m, 3H), 1.53 – 1.43 (m, 2H), 1.37 – 1.17 (m, 6H), 1.11 – 0.92 (m, 18H). **^{13}C NMR** (101 MHz, $CDCl_3$) δ 74.8, 74.7, 72.3, 72.2, 56.2, 56.1, 52.0, 51.9, 51.8, 42.1, 42.1, 41.6, 37.4, 37.3, 37.0, 32.8, 32.7, 32.3, 31.9, 31.8, 31.7, 30.1, 30.0, 29.7, 29.5, 29.5, 29.5, 29.4, 29.2, 29.1, 29.0, 25.7, 24.1, 21.1, 20.8, 20.7, 19.7, 18.2, 14.1. **HRMS** (ESI/TOF) *m/z*: $[M + Na]^+$ Calcd. for $C_{23}H_{44}NaO_2S_2$: 439.2675; found 439.2663. **FT-IR** (neat) ν(cm^{-1}) 3415, 1671, 1452, 970.

Synthesis of aziridine 20

20

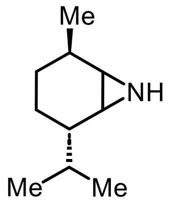

The 1,2-azido-alcohol **3** (140 mg, 706 µmol) and triphenylphosphine (222 mg, 847 µmol) were dissolved in THF (3.5 mL) and the reaction mixture was kept at reflux temperature for 4 h. the solvent was removed under reduced pressure and the residue dissolved in diethyl ether, and the precipitate removed via filtration. The residue was purified via column chromatography (hexane:ethyl acetate, 9:1 to 8:2 v/v), obtaining the product as a colorless oil (80 mg, 74% yield, *rr* = 7:3). **^{1}H NMR** (400 MHz, $CDCl_3$) δ 2.27 – 2.22 (m, major), 2.13 – 2.09 (m, minor), 1.98 (d, *J* = 6.1 Hz, major), 1.84 (d, *J* = 6.1 Hz, minor), 1.75 – 1.24 (m, 7H), 1.07 – 1.00 (m, 5H), 0.96 – 0.92 (m, 4H). **^{13}C NMR** (101 MHz, $CDCl_3$) δ 42.1, 42.0, 36.5, 36.1, 34.1, 33.5, 32.4, 32.1, 30.6, 30.5, 29.8, 26.8, 25.8, 22.7, 22.0, 20.9, 20.8, 20.1, 19.8, 19.7. **HRMS** (ESI/TOF) *m/z*: $[M + H]^+$ Calcd. for $C_{10}H_{20}N$: 154.1590; found 154.1588. **FT-IR** (neat) ν(cm^{-1}) 1743, 1678, 1459.

Synthesis of bis-epoxide 21

21

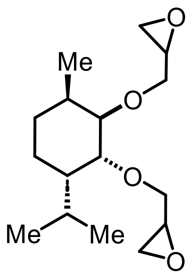

To a stirred solution of the diallyl product **13** (250 mg, 990 µmol) in DCM (2 mL) cooled by an ice bath, was added *m*-CPBA (570 mg, 2.48 mmol) in DCM (4 mL). The reaction mixture was allowed to reach room temperature and stirred for 24 h. Then, it was filtered and washed with saturated $Na_2S_2O_3$ (4 times), 10% $NaHCO_3$ (1 time) and dried over Na_2SO_4 The solvent was removed under reduced pressure and the residue was purified via column chromatography (hexane:ethyl acetate, 8:2 v/v), obtaining the product as a colorless oil (244 mg, 87% yield). **^{1}H NMR** (400 MHz, $CDCl_3$) δ 3.83 – 3.58 (m, 3H), 3.52 – 3.34 (m, 3H), 3.19 – 3.04 (m, 2H), 2.81 – 2.74 (m, 2H), 2.64 – 2.55 (m, 2H), 1.89 – 1.72 (m, 1H), 1.67 – 1.62 (m, 1H), 1.57 – 1.44 (m, 1H), 1.37 – 1.20 (m, 4H), 1.00 – 0.88 (m, 9H). **^{13}C NMR** (101 MHz, $CDCl_3$) δ 80.0, 79.5, 79.4, 78.9, 77.4, 76.9, 76.8, 76.2, 72.4, 72.3, 71.3, 71.3, 71.1, 71.1, 70.3,

70.2, 51.4, 51.3, 51.3, 51.3, 51.2, 44.5, 44.5, 44.3, 44.3, 44.2, 44.2, 44.1, 44.0, 42.3, 42.3, 42.2, 30.6, 30.6, 30.6, 28.6, 28.6, 28.4, 28.4, 23.9, 21.1, 21.1, 21.0, 17.9, 17.8. **HRMS** (ESI/TOF) *m/z*: $[M + Na]^+$ Calcd. for $C_{16}H_{28}NaO_4$: 307.1880; found 307.1870. **FT-IR** (neat) ν (cm^{-1}) 1081.

Synthesis of bis-cyclic carbonate 22

22

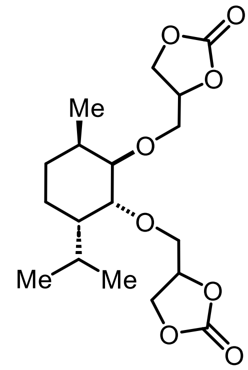

Bis-epoxide (88.0 mg, 309 µmol), **Al^Cl^** (2.0 mg, 3.1 µmol),[7] tetrabutylammonium bromide (10.0 mg, 31 µmol) and MEK (0.31 mL) were mixed in a vial equipped with a magnetic stirring bar, placed in a stainless-steel reactor and purged three times with 5 bar of CO_2. Finally, the pressure was stabilized at 20 bar of CO_2 at r.t. After placing the reactor in a metal heating block, the reactor was heated to an inside temperature of 70 °C (82 °C outside temperature) for 24 h stirring at 600 rpm. The reaction was stopped by cooling the reactor in an ice bath and subsequent depressurizing it slowly. The solvent was removed under reduced pressure and the residue was purified via column chromatography (hexane:ethyl acetate, 95:5 to 8:2 v/v), obtaining the product as a colorless oil (91 mg, 79% yield). **^{1}H NMR** (400 MHz, $CDCl_3$) δ 4.88 – 4.70 (m, 2H), 4.56 – 4.29 (m, 4H), 3.86 – 3.80 (m, 1H), 3.79 – 3.55 (m, 4H), 3.45 – 3.37 (m, 1H), 1.81 – 1.70 (m, 1H), 1.59 – 1.55 (m, 2H), 1.41 – 1.34 (m, 1H), 1.24 – 1.13 (m, 3H), 0.98 – 0.87 (m, 9H). **^{13}C NMR** (101 MHz, $CDCl_3$) δ 155.1, 155.0, 154.9, 80.4, 80.2, 80.1, 79.9, 77.4, 77.3, 77.0, 75.4, 75.4, 75.4, 75.2, 75.1, 75.1, 70.4, 70.4, 70.3, 69.9, 69.5, 69.3, 69.2, 69.2, 66.4, 66.3, 66.3, 66.2, 66.1, 66.1, 66.0, 66.0, 42.8, 42.7, 42.6, 31.2, 31.1, 30.9, 28.7, 28.7, 28.5, 28.3, 28.3, 28.2, 24.1, 24.0, 21.3, 21.3, 21.2, 21.2, 21.1, 21.1, 21.0, 18.2, 18.2, 18.0, 17.9. **HRMS** (ESI/TOF) *m/z*: $[M + H]^+$ Calcd. for $C_{18}H_{29}O_8$: 373.1857; found 373.1851. **FT-IR** (neat) ν (cm^{-1}) 1786.

Synthesis of bis-acrylic ether 23

23

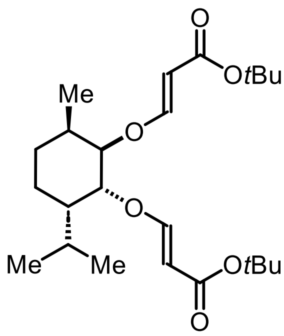

In an argon-filled vial charged with a stirring bar, menthene diol (70 mg, 406 µmol) was dissolved in DCM (700 µL), and then *tert*-butyl propiolate (133 mg, 145 µL, 2.6 equiv, 1.06 mmol) and DABCO (9.12 mg, 0.2 equiv, 81.3 µmol) were sequentially added. The reaction mixture was stirred at 25 °C for 4 hours. Hereafter, the solvent was removed under reduced pressure and the reaction mixture purified by preparative TLC (hexane:ethyl acetate 8:2 v/v), obtaining the purified product as a white solid (153 mg, 89% yield, *E/Z* = 85:15). **^{1}H NMR** (400 MHz, $CDCl_3$) δ 7.47 (d, *J* = 12.2 Hz, minor) 7.41 (d, *J* = 12.3 Hz, major), 5.57 (d, *J* = 12.2 Hz, minor), 5.32 – 5.34 (m, major), 4.18 – 4.15 (m, 1H), 3.95 – 3.90 (m, 1H), 1.91 – 1.79 (m, 1H), 1.71 – 1.46 (m, 23H), 1.42 – 1.25 (m, 2H), 1.00 – 0.81 (m, 9H). **^{13}C NMR** (101 MHz, $CDCl_3$) δ 167.4, 167.3, 165.6, 161.7, 160.8, 156.8, 105.9, 100.0, 99.9, 82.3, 81.0, 80.1, 80.1, 79.3, 41.6, 30.4, 28.6, 28.5, 28.3, 28.0, 23.9, 20.9, 20.8, 17.5. **HRMS** (ESI/TOF) *m/z*: $[M + Na]^+$ Calcd. for $C_{24}H_{40}NaO_6$: 447.2717; found 447.2723. **FT-IR** (neat) ν (cm^{-1}) 1699, 1637, 1621, 1113.

8. Copies of NMR and IR spectra of all compounds

Analytical data for PMC

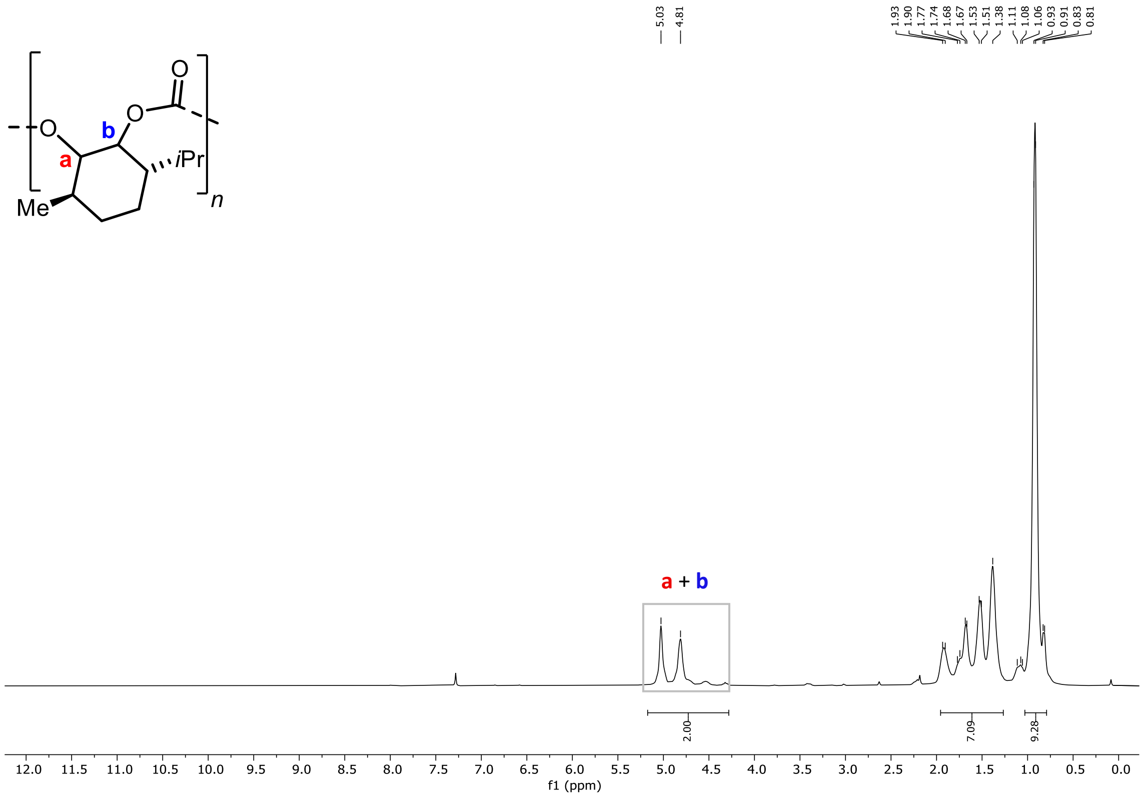

Figure S1. ^{1}H NMR spectrum ($CDCl_3$, 400 MHz) of **PMC** (entry 9, Table 1).

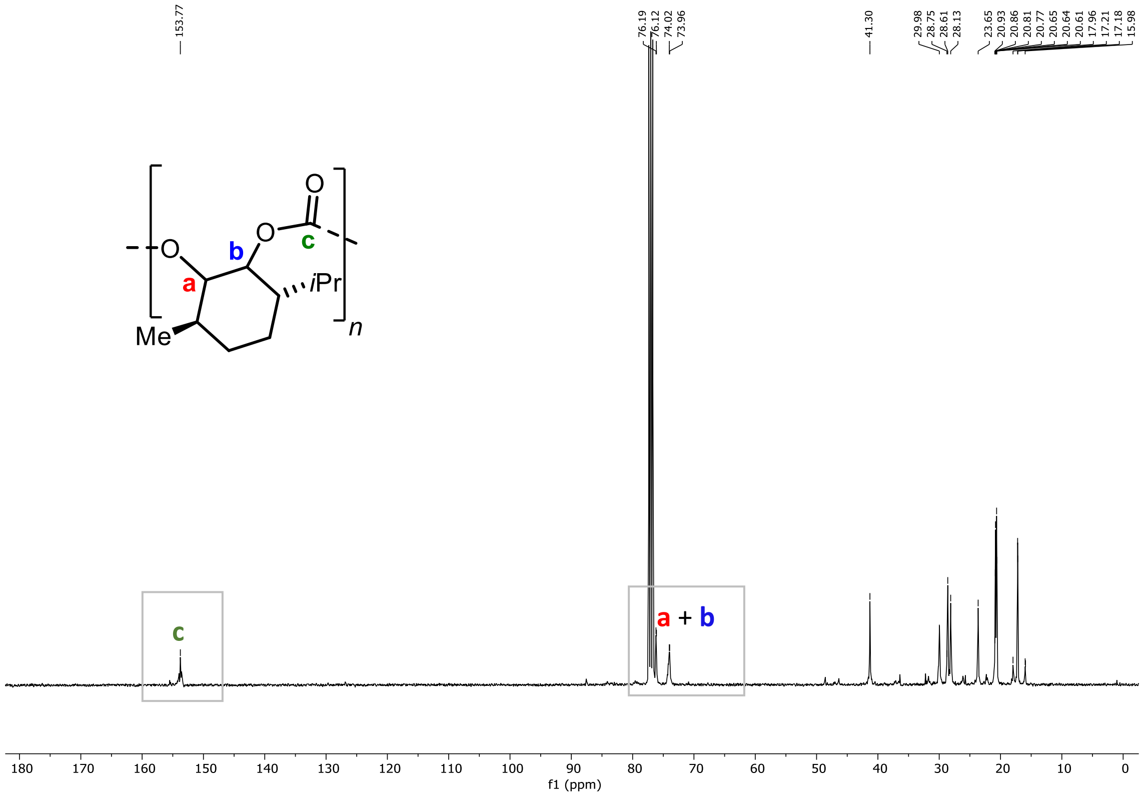

Figure S2. ^{13}C NMR spectrum ($CDCl_3$, 101 MHz) of **PMC** (entry 9, Table 1)

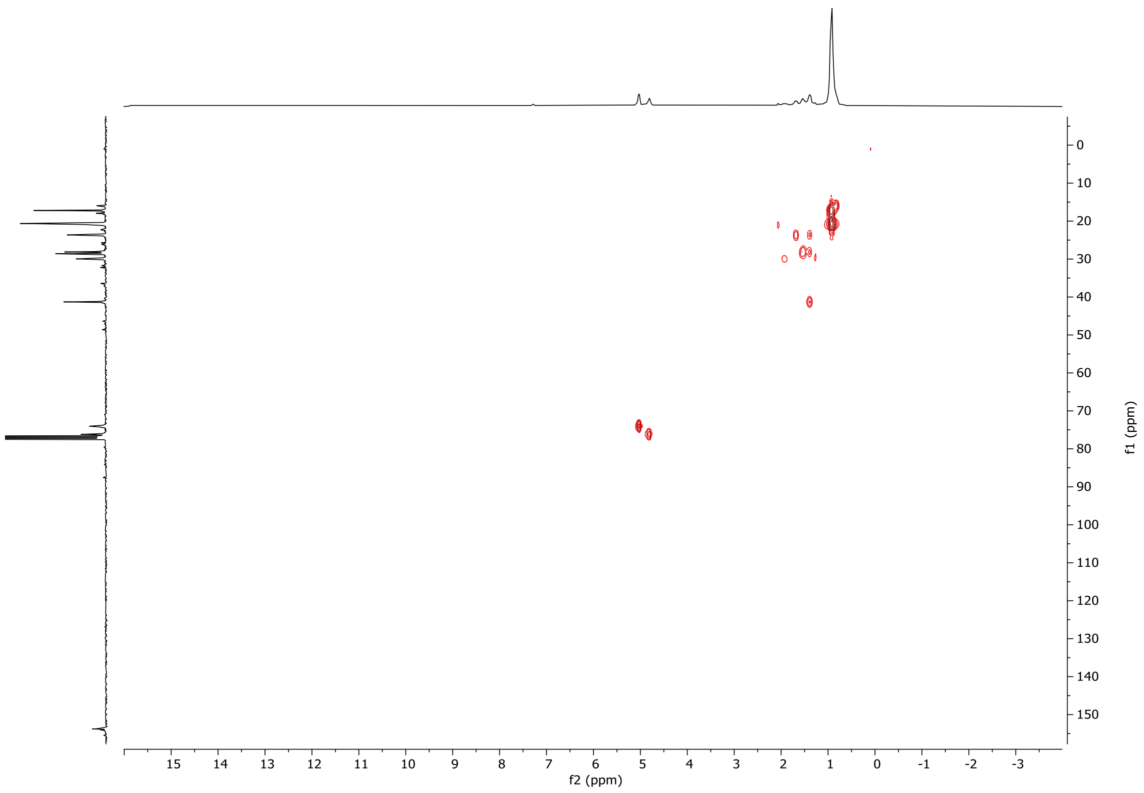

Figure S3. ^{1}H-^{13}C HMQC NMR spectrum ($CDCl_3$) of **PMC** (entry 9, Table 1).

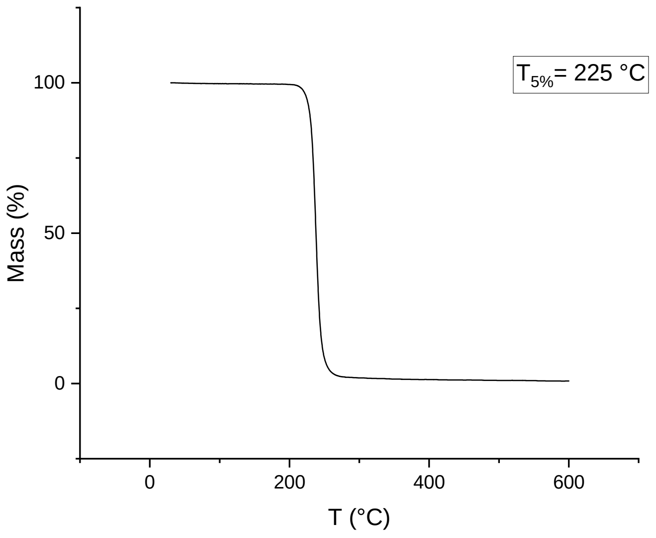

Figure S4. TGA analysis of **PMC** (entry 9, Table 1).

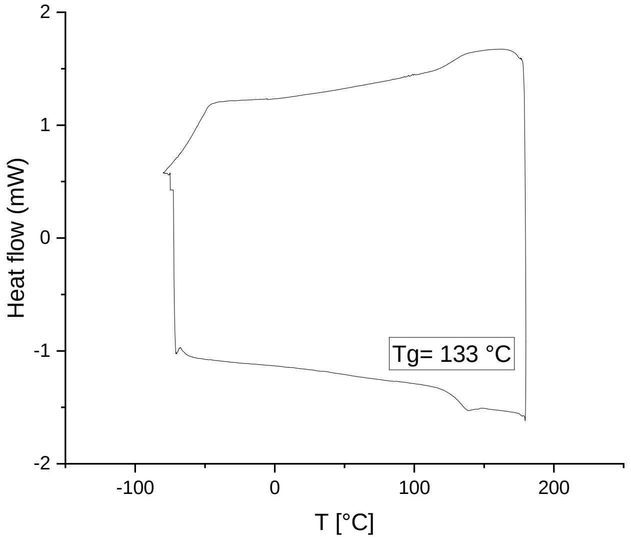

Figure S5. DSC analysis of **PMC** (entry 9, Table 1).

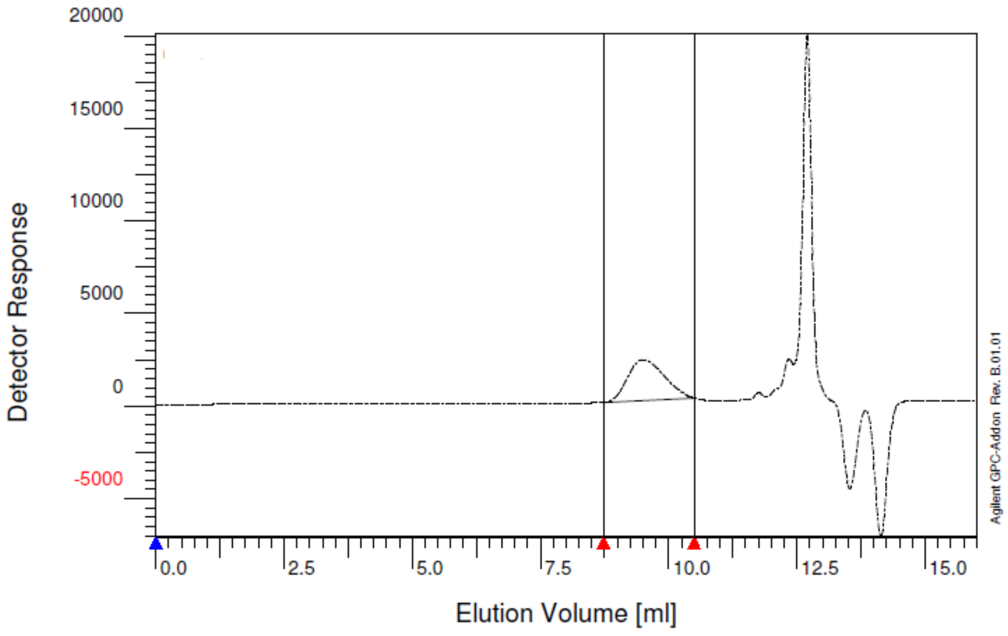

M_n (g/mol)	M_w (g/mol)	M_z (g/mol)	M_p (g/mol)	*Đ*
7.0043e3	8.5409e3	1.0146e4	8.8487e3	1.2194e0

Figure S6. GPC analysis of **PMC** (entry 9, Table 1)

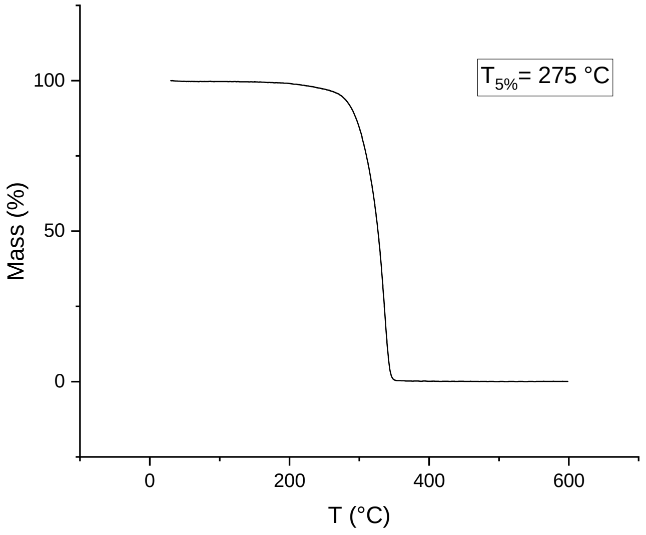

Figure S7. TGA analysis of **PMC** (entry 19, Table 1).

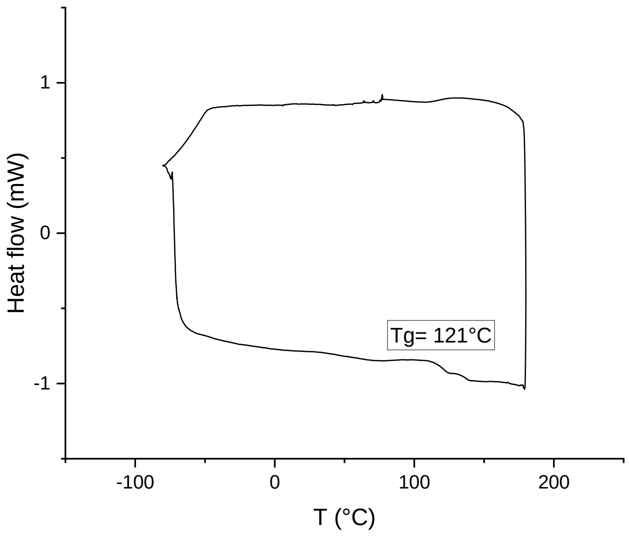

Figure S8. TGA analysis of **PMC** (entry 19, Table 1)

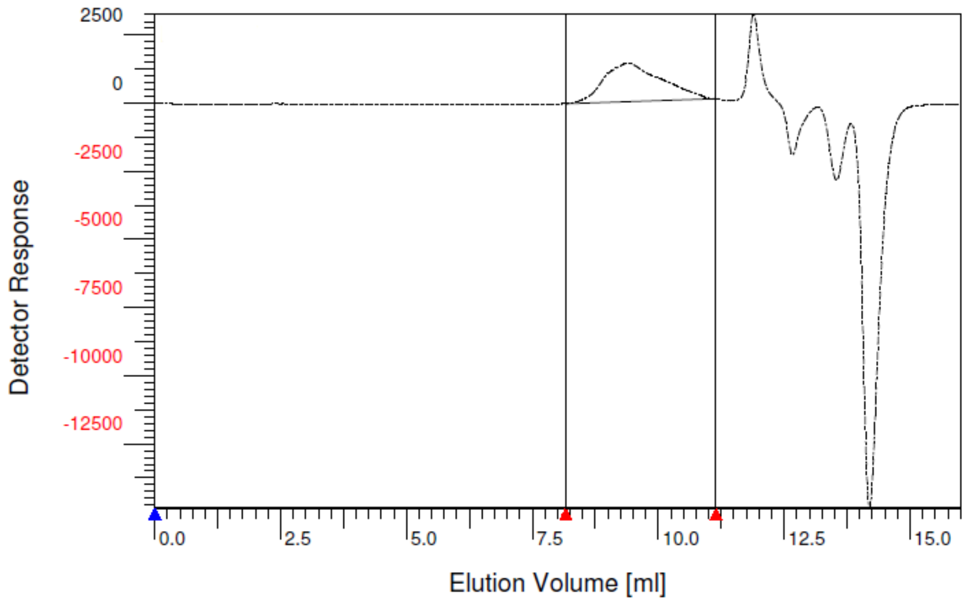

M_n (g/mol)	M_w (g/mol)	M_z (g/mol)	M_p (g/mol)	*Đ*
5.9223e3	1.0442e4	1.5815e4	1.0321e4	1.7632e0

Figure S9. GPC analysis of **PMC** (entry 19, Table 1).

Analytical data for MC:

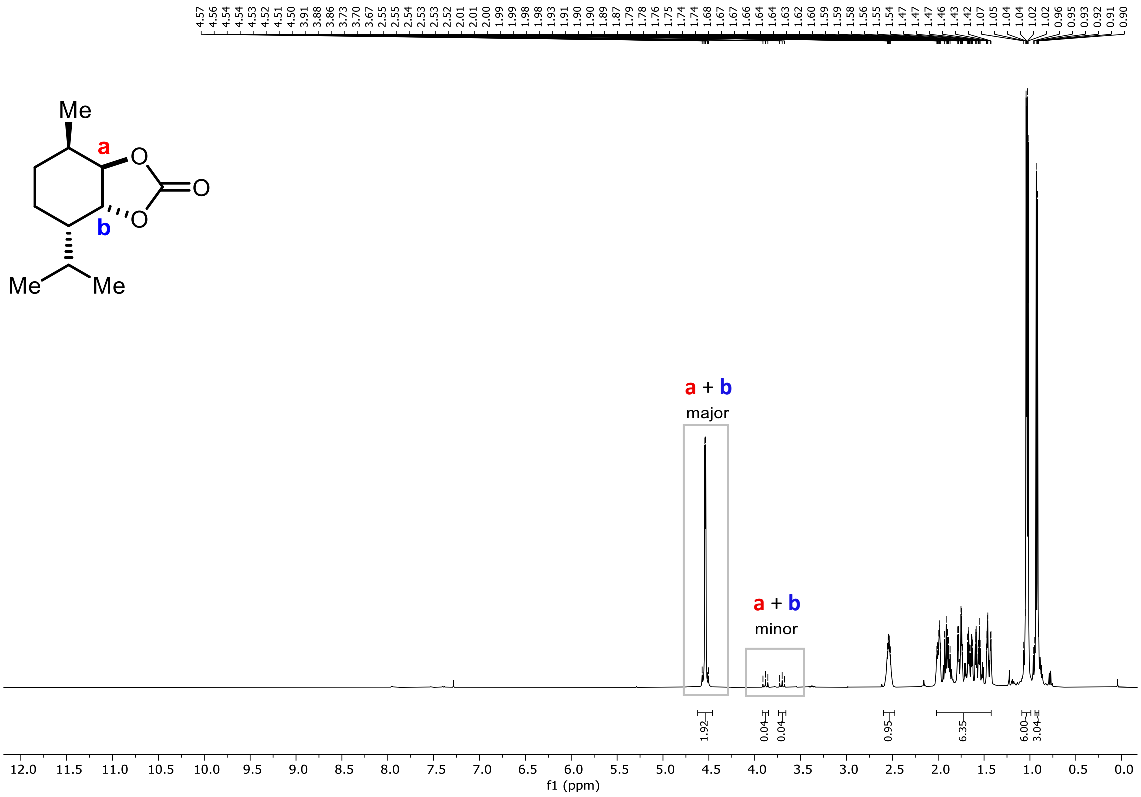

Figure S10. ^{1}H NMR spectrum ($CDCl_3$, 400 MHz) of **MC** (*dr* > 20:1).

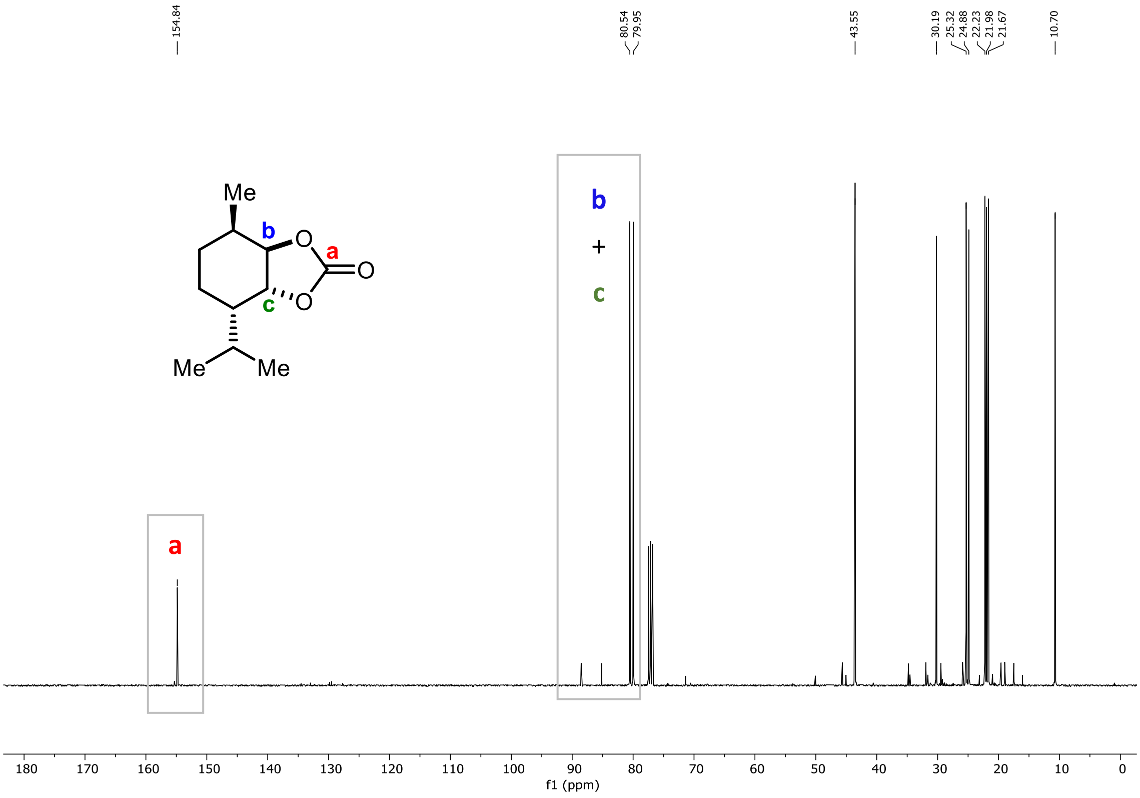

Figure S11. ^{13}C NMR spectrum ($CDCl_3$, 101 MHz) of **MC** (*dr* > 20:1).

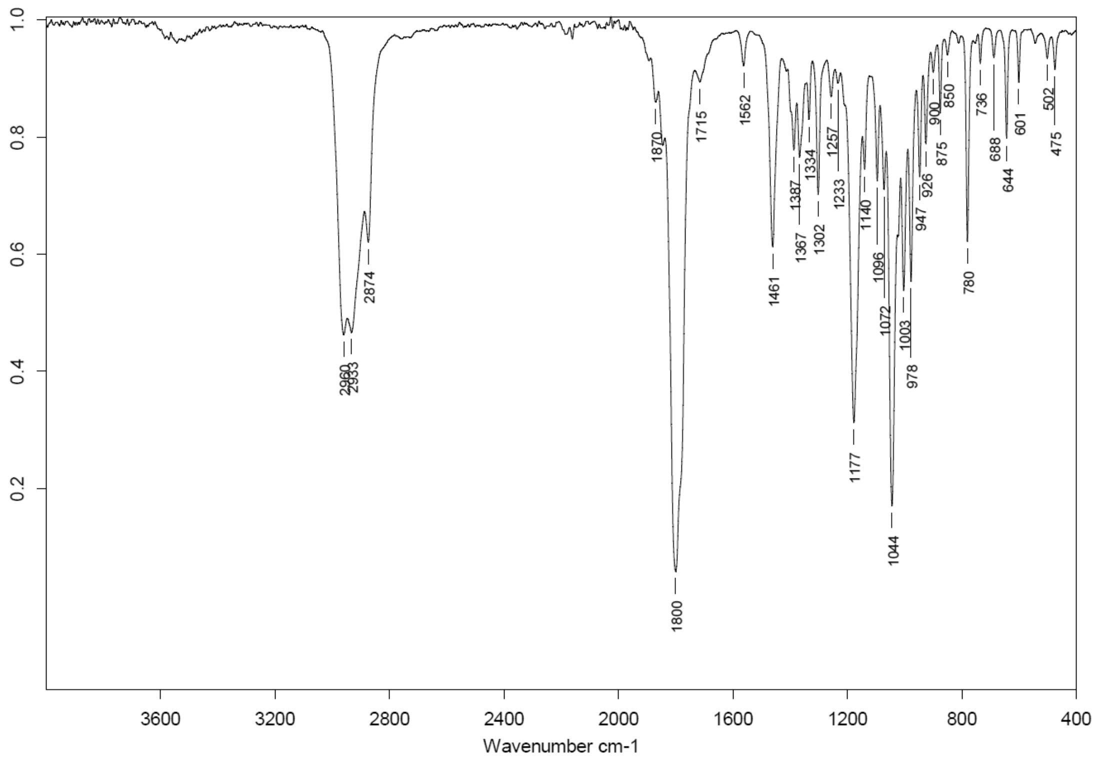

Figure S12. IR spectrum of **MC** (*dr* > 20:1).

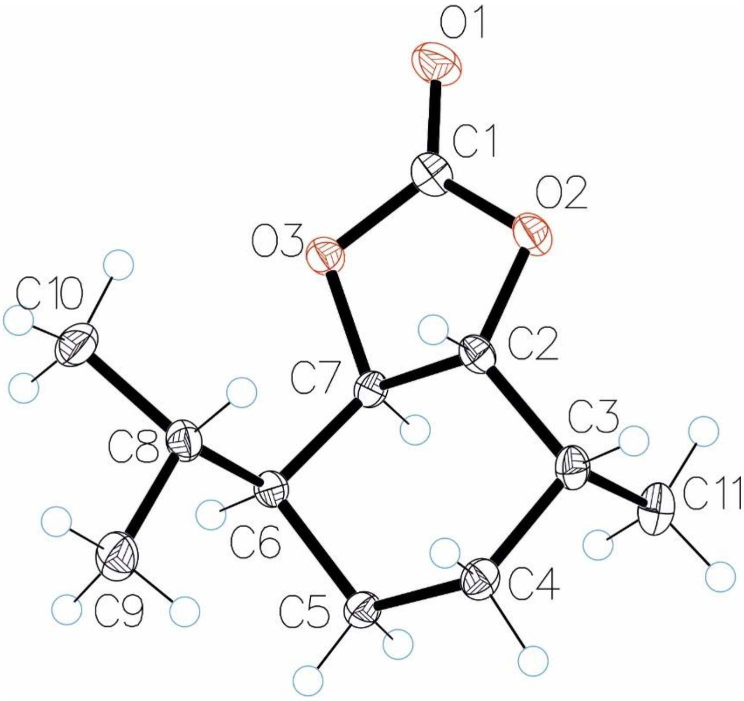

Figure S13. Molecular structure of **MC** (*dr* > 20:1). More details can be found in CCDC-2455347.

Analytical data for compound **1**

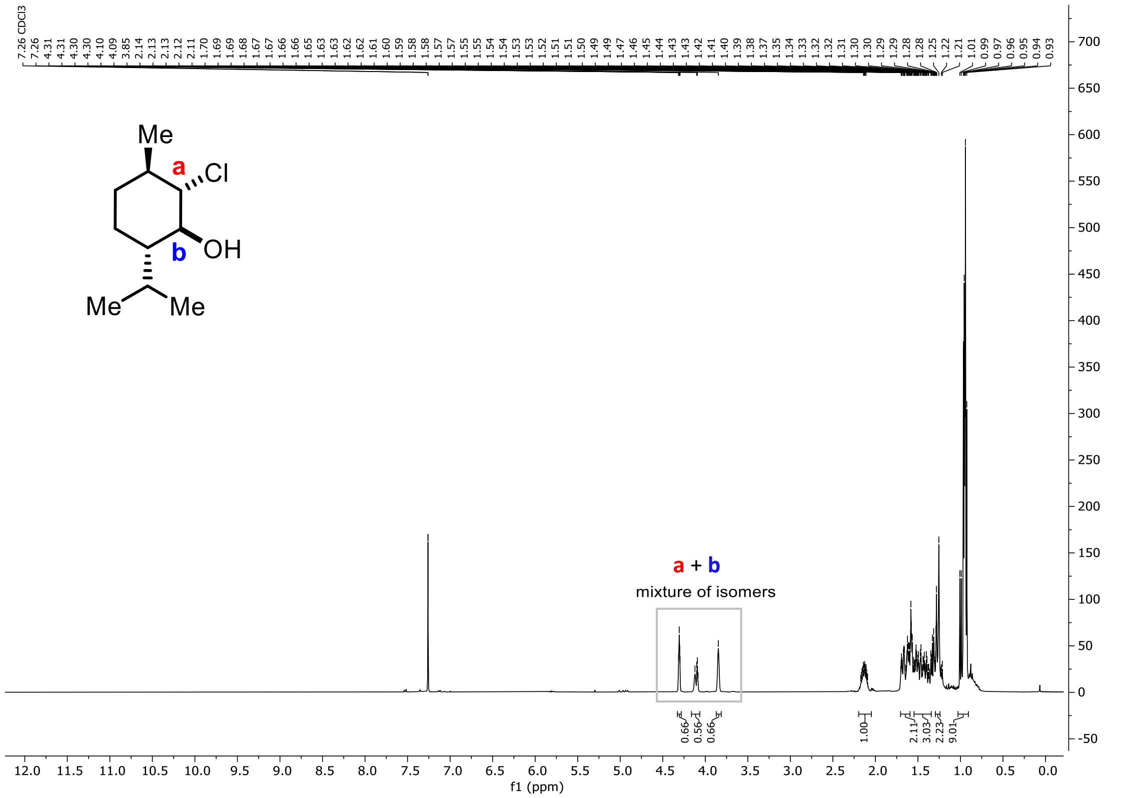

Figure S14. ^{1}H NMR spectrum ($CDCl_3$, 400 MHz) of compound **1**.

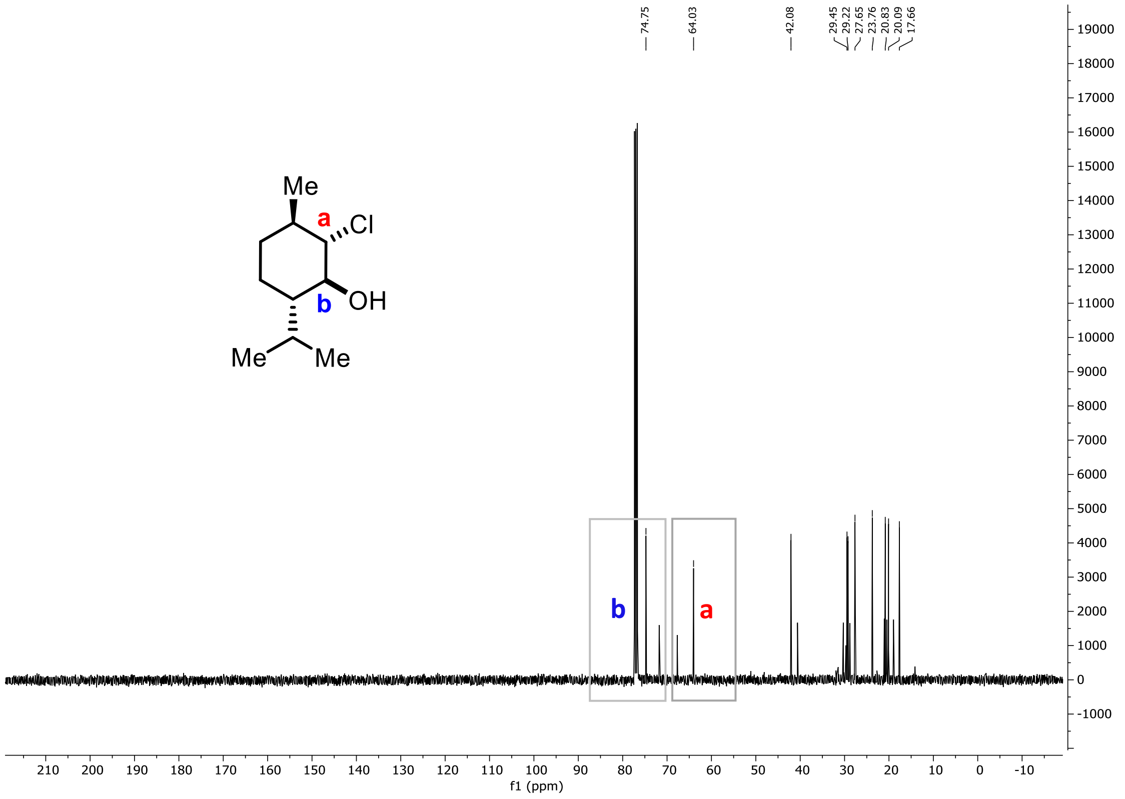

Figure S15. ^{13}C NMR spectrum ($CDCl_3$, 101 MHz) of compound **1**.

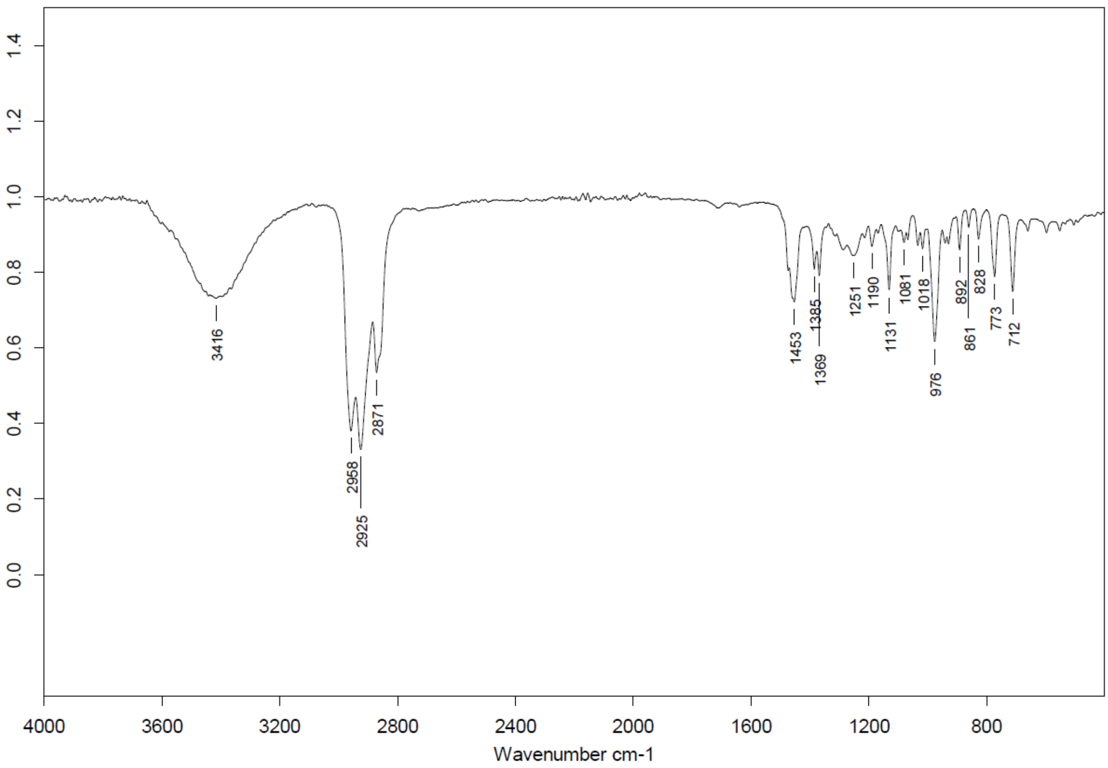

Figure S16. IR spectrum (neat) of compound **1**.

Analytical data for compound **2**

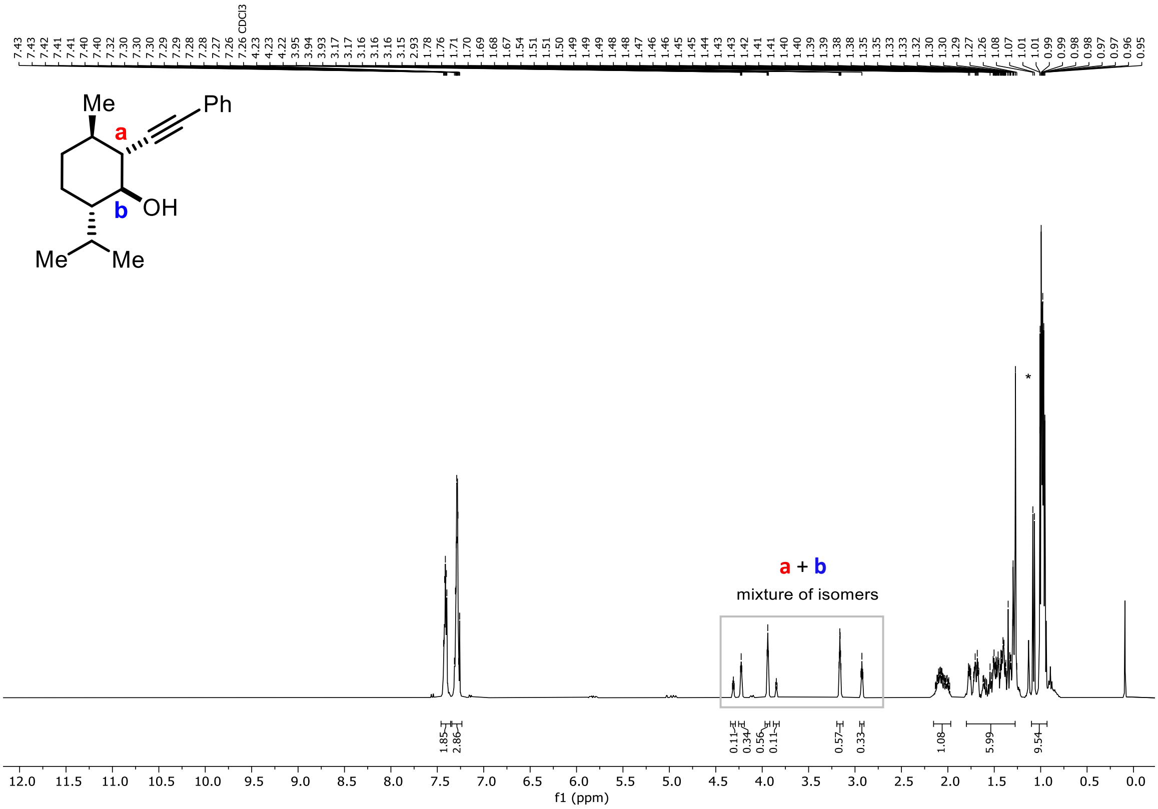

Figure S17. ^{1}H NMR spectrum ($CDCl_3$, 400 MHz) of compound **2**.

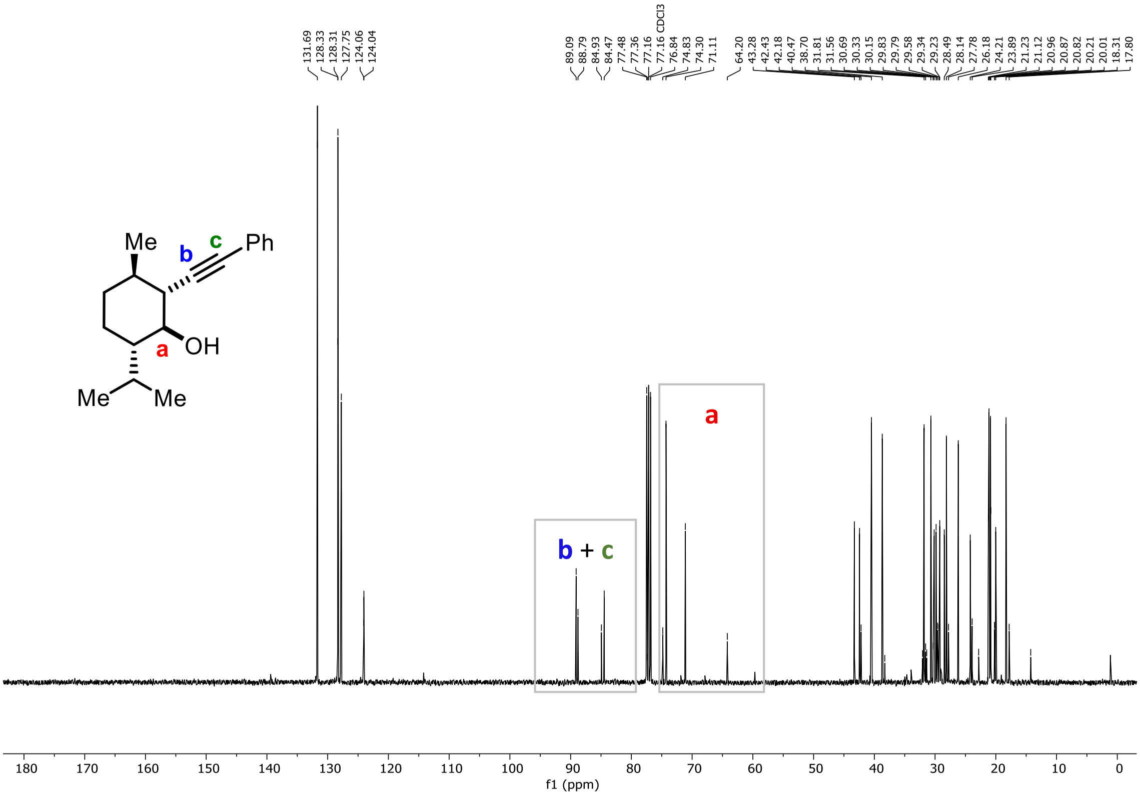

Figure S18. ^{13}C NMR spectrum ($CDCl_3$, 101 MHz) of compound **2**.

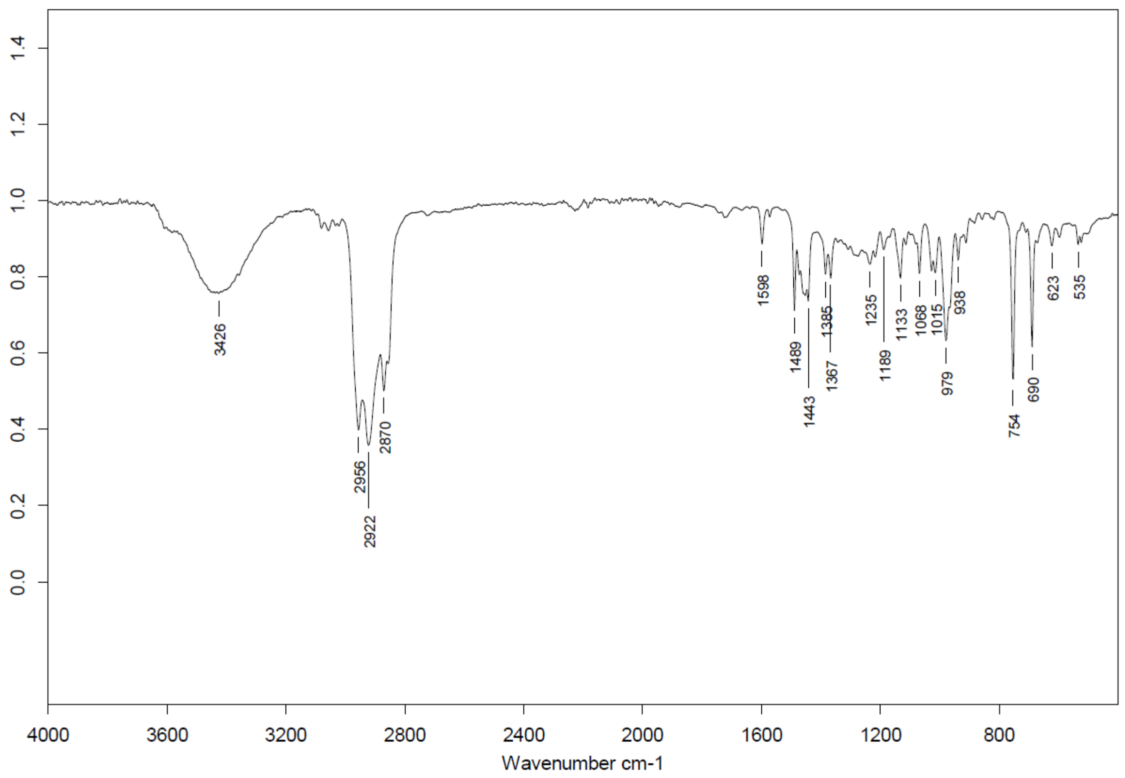

Figure S19. IR spectrum (neat) of compound **2**.

Analytical data for compound **3**

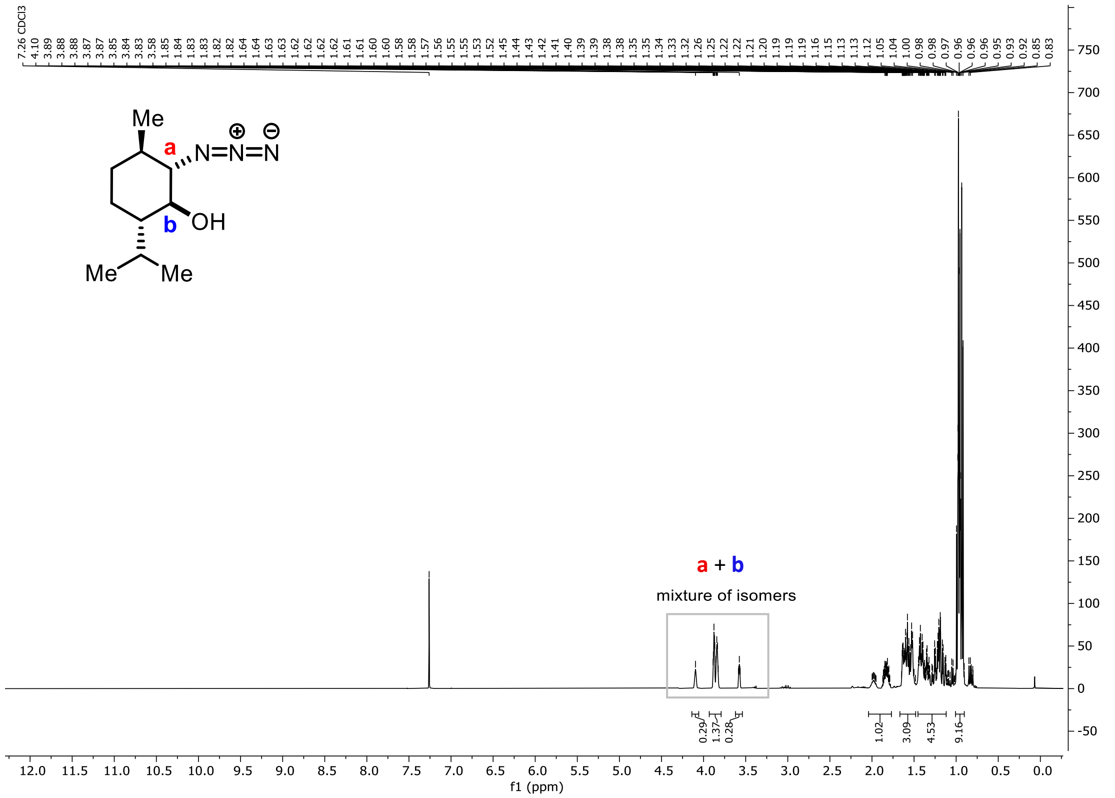

Figure S20. ^{1}H NMR spectrum ($CDCl_3$, 400 MHz) of compound **3**.

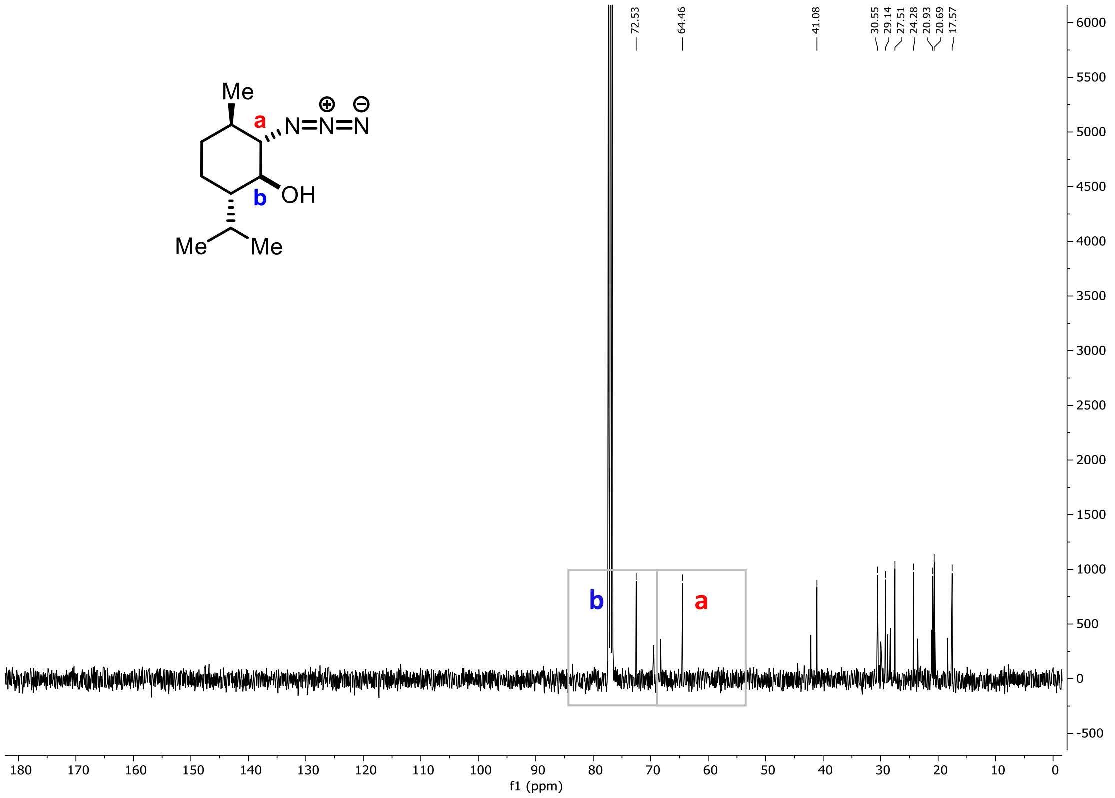

Figure S21. ^{13}C NMR spectrum ($CDCl_3$, 101 MHz) of compound **3**.

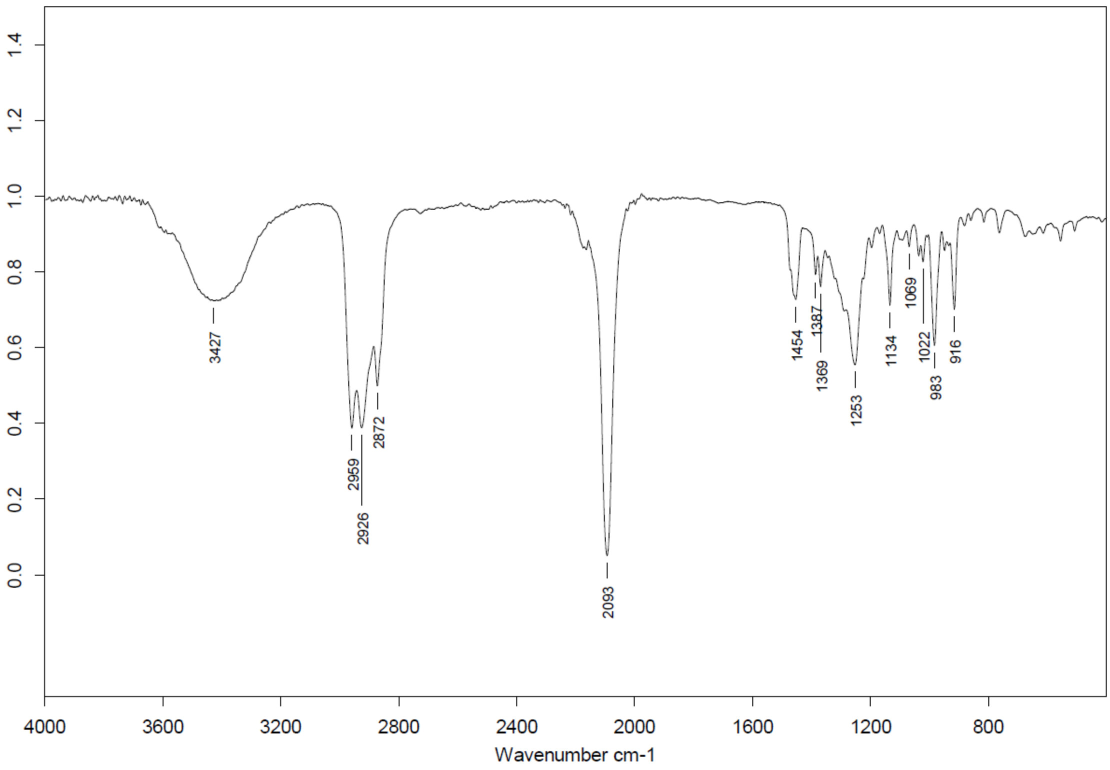

Figure S22. IR spectrum (neat) of compound **3**.

Analytical data for compound **4**

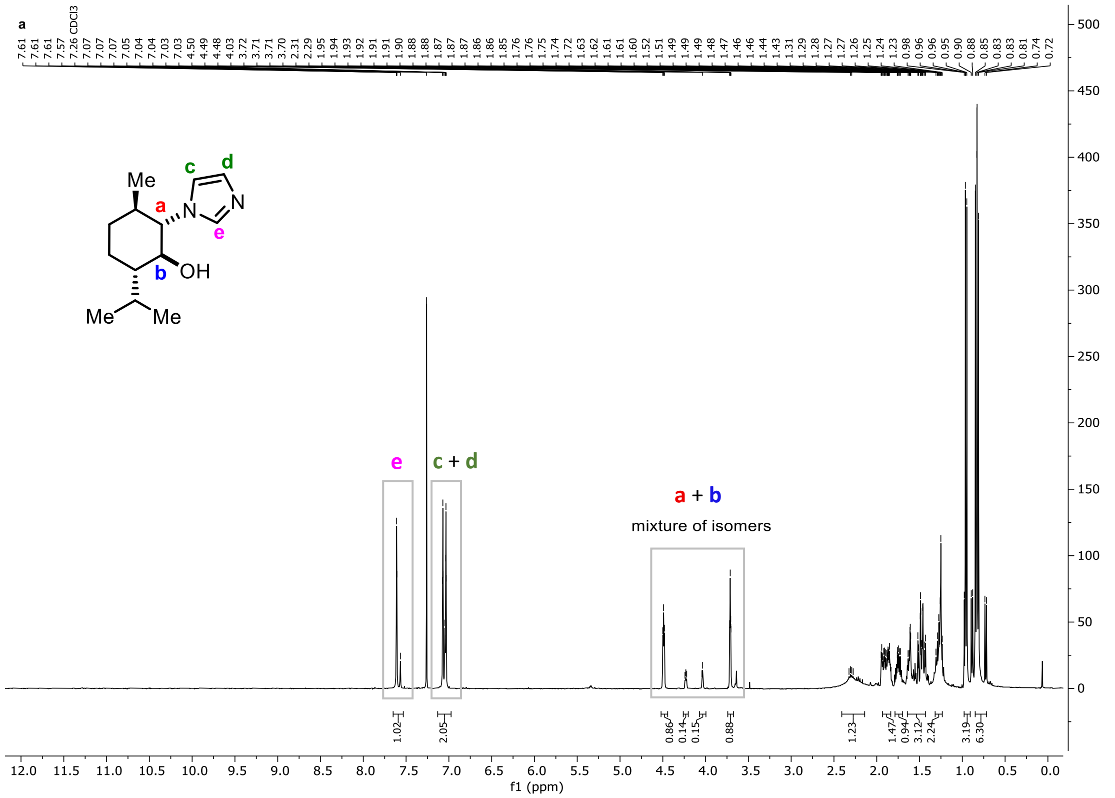

Figure S23. ^{1}H NMR spectrum ($CDCl_3$, 400 MHz) of compound **4**.

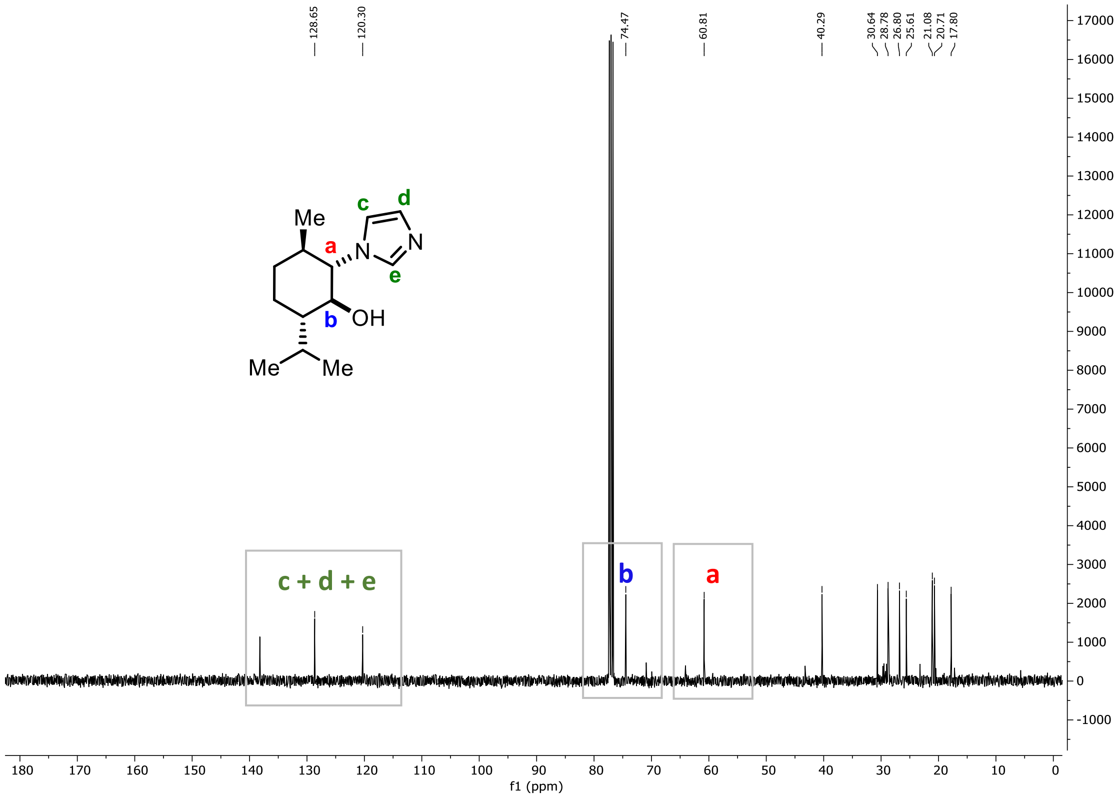

Figure S24. ^{13}C NMR spectrum ($CDCl_3$, 101 MHz) of compound **4**.

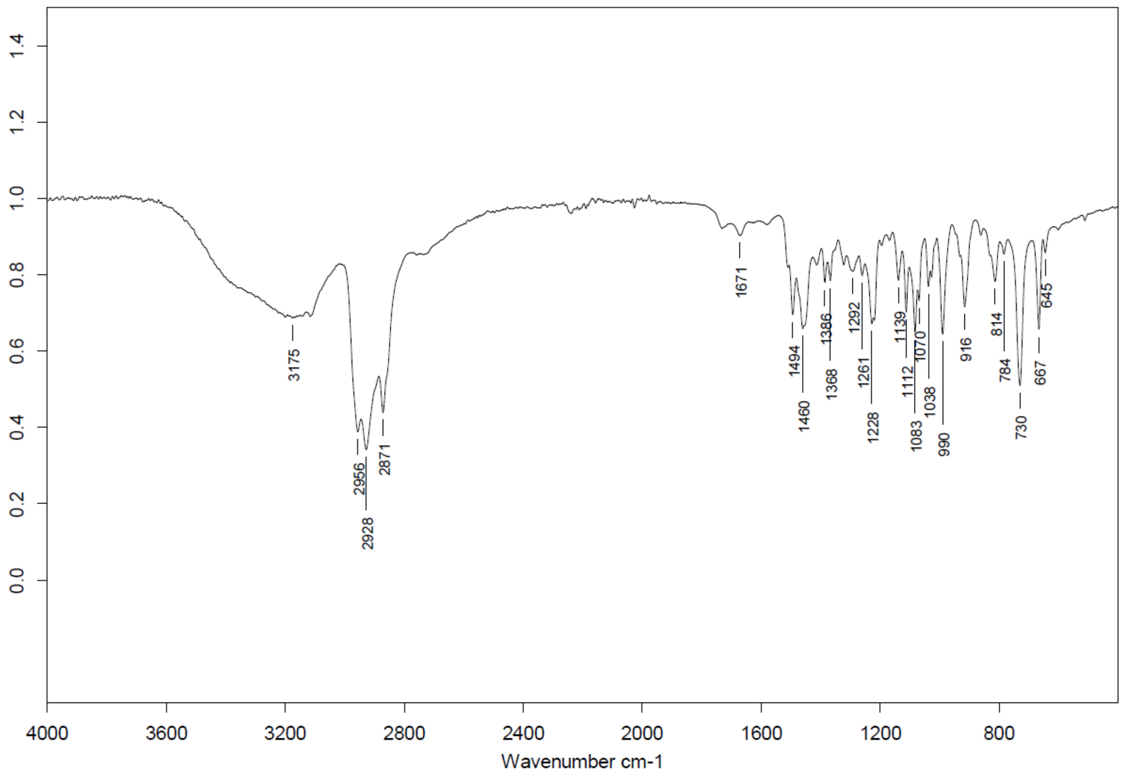

Figure S25. IR spectrum (neat) of compound **4**.

Analytical data for compound **5**

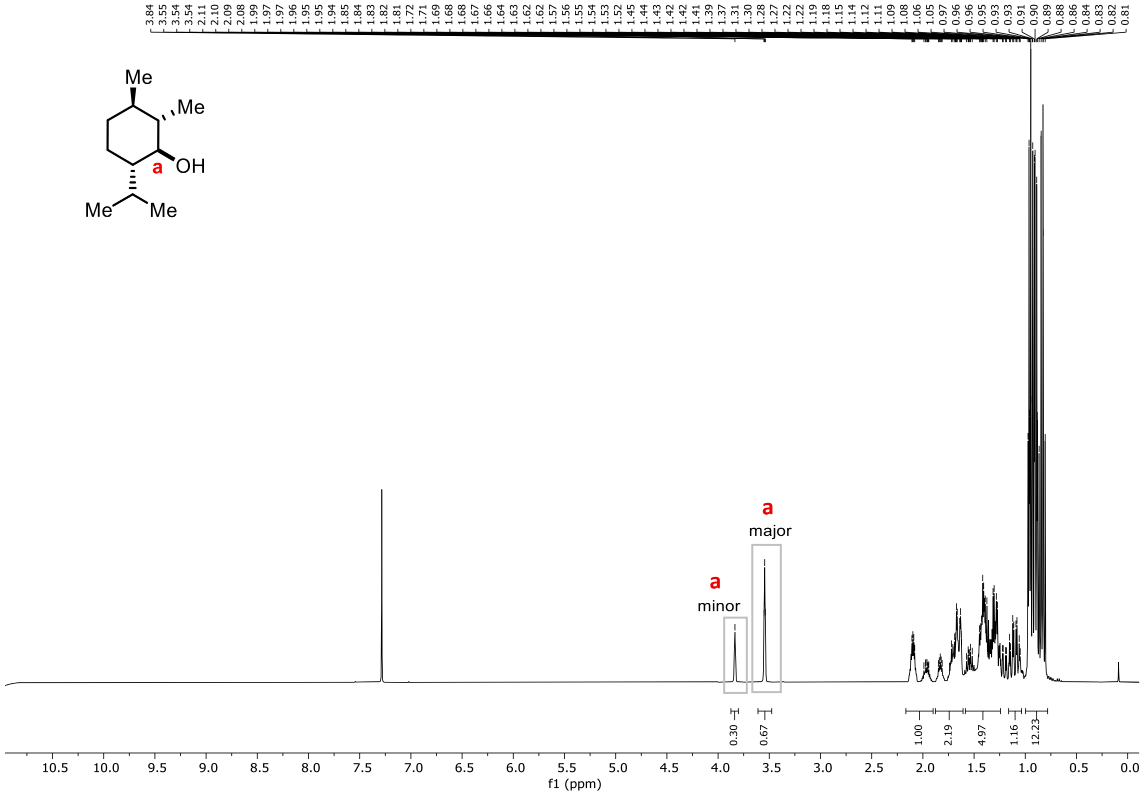

Figure S26. ^{1}H NMR spectrum ($CDCl_3$, 400 MHz) of compound **5**.

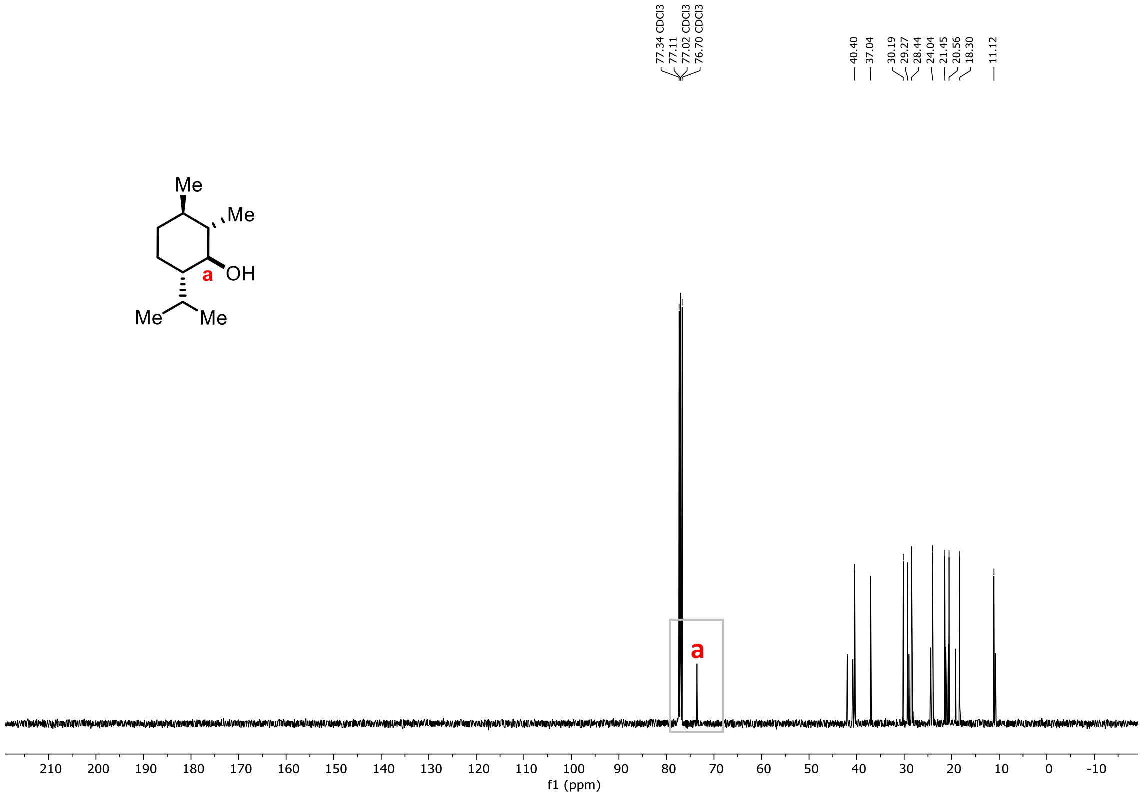

Figure S27. ^{13}C NMR spectrum ($CDCl_3$, 101 MHz) of compound **5**.

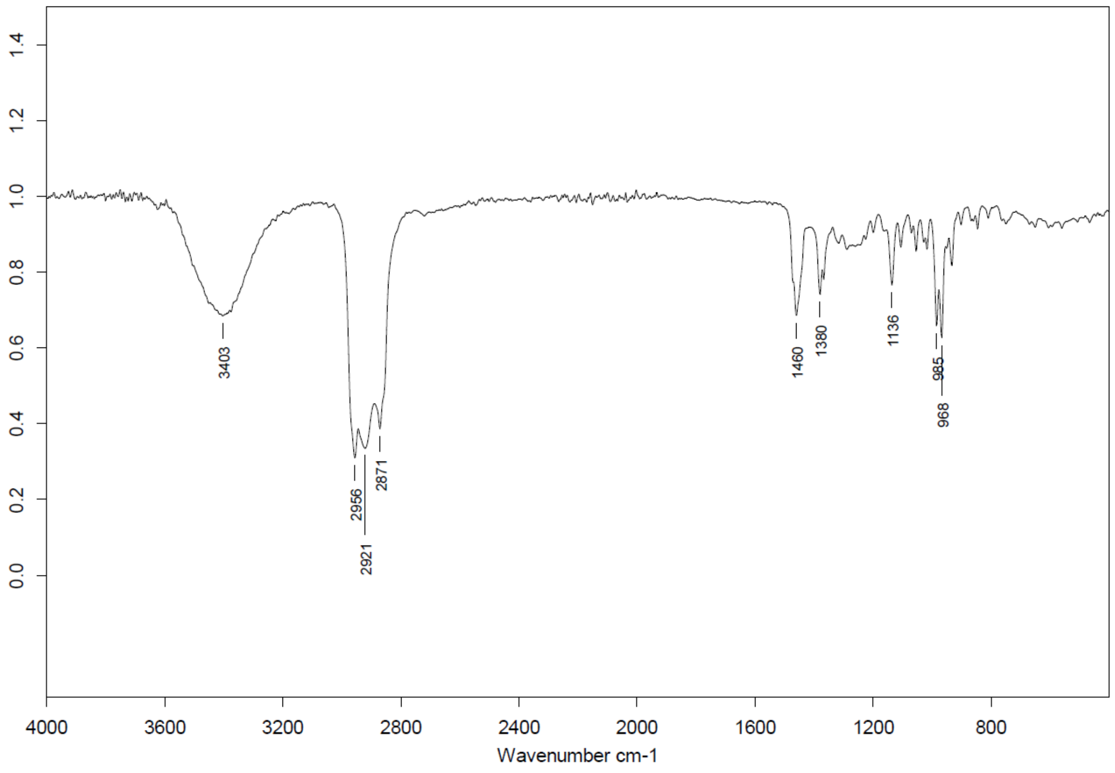

Figure S28. IR spectrum (neat) of compound **5**.

Analytical data of compound **6**

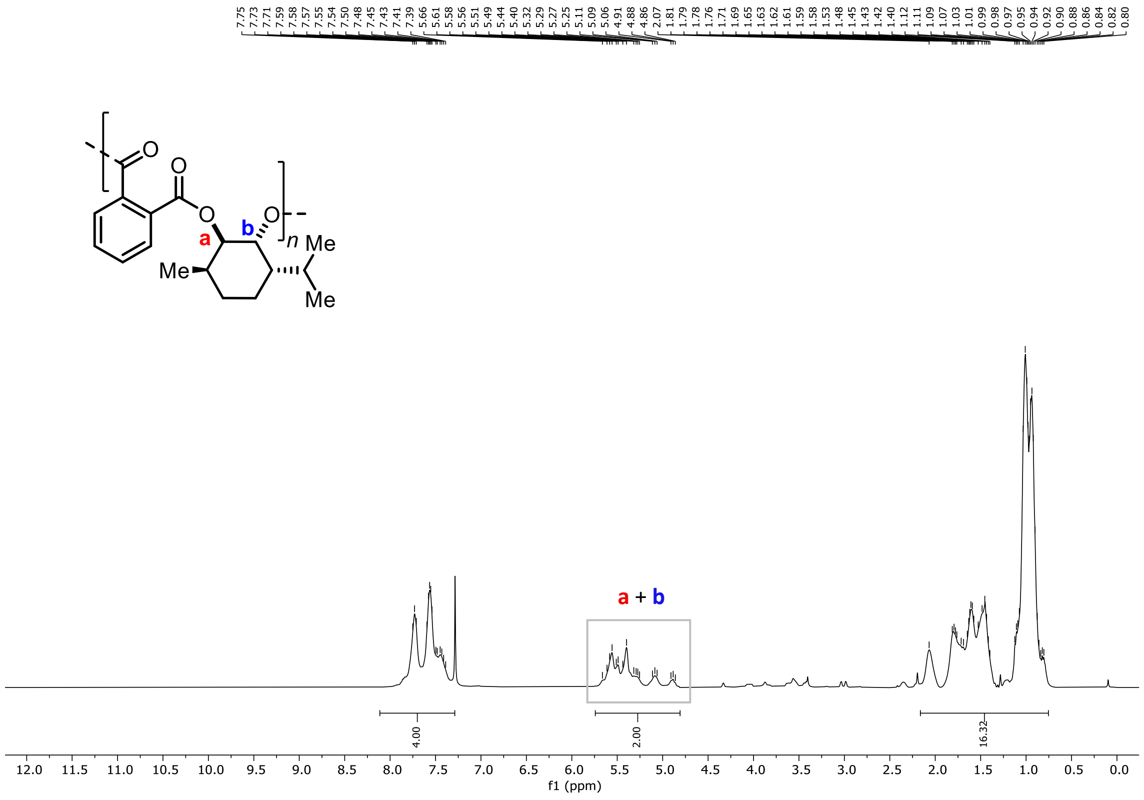

Figure S29. ^{1}H NMR spectrum ($CDCl_3$, 400 MHz) of compound **6**.

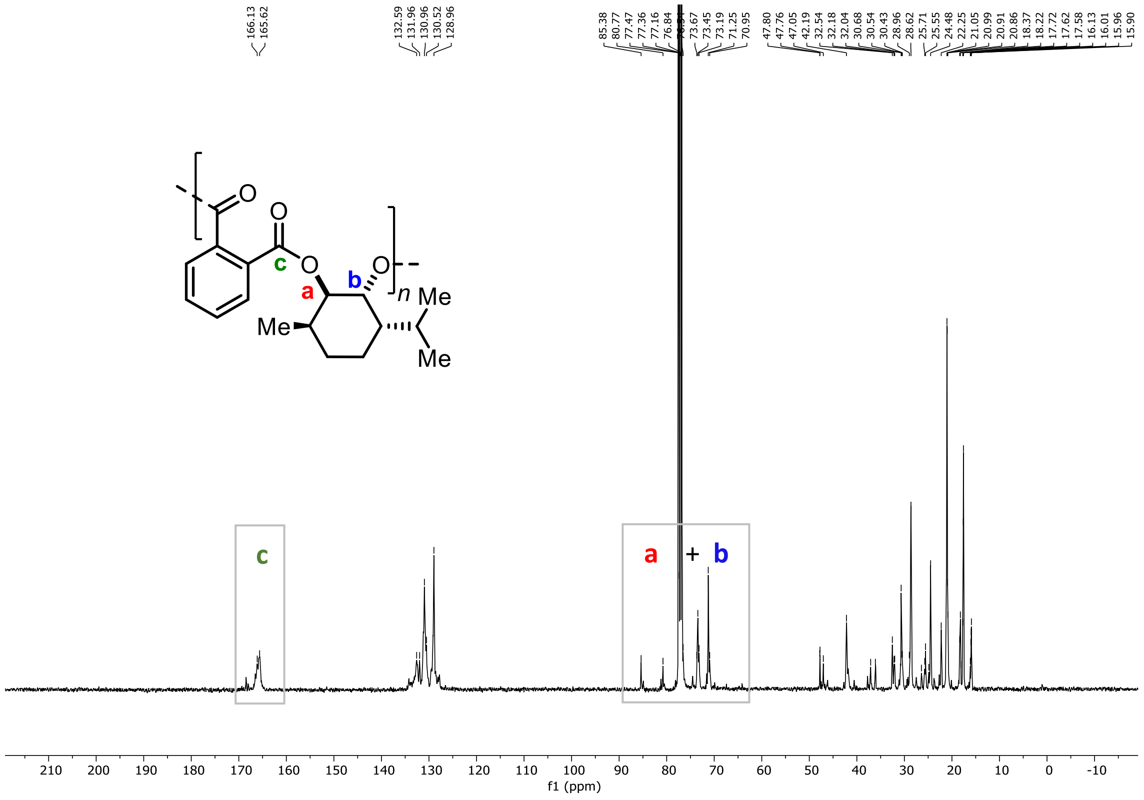

Figure S30. ^{13}C NMR spectrum ($CDCl_3$, 101 MHz) of compound **6**.

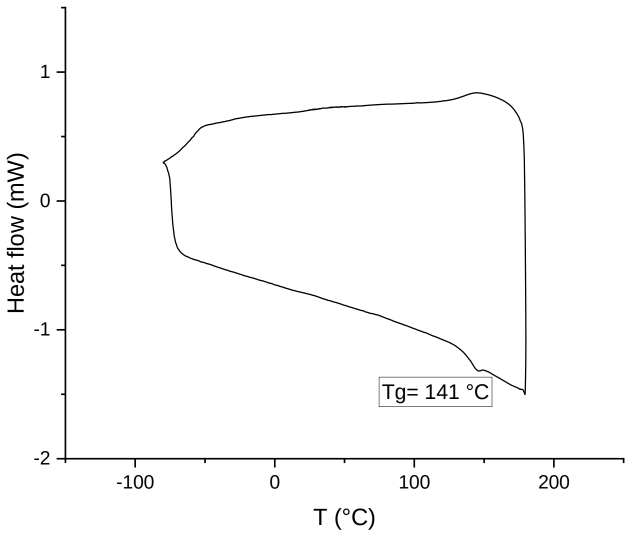

Figure S31. DSC analysis of compound **6**.

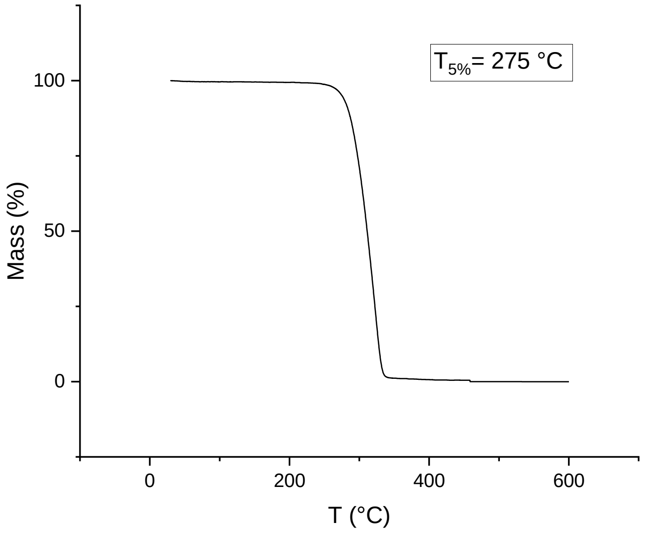

Figure S32. TGA analysis of compound **6**.

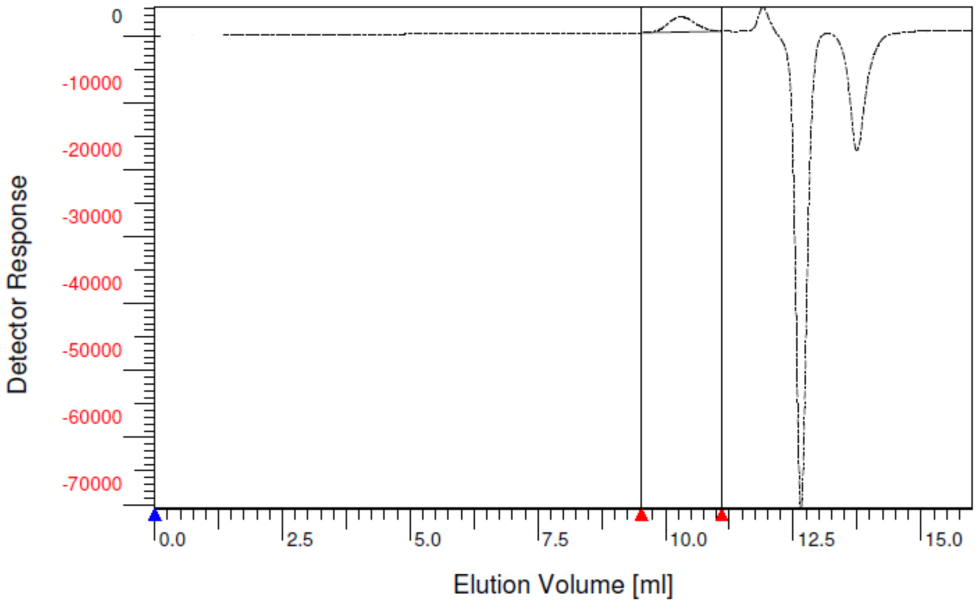

M_n (g/mol)	M_w (g/mol)	M_z (g/mol)	M_p (g/mol)	*Đ*
3.5568e3	3.8866e3	4.2338e3	3.8518e3	1.0927e0

Figure S33. GPC analysis of compound **6**.

Analytical data for compound **7**

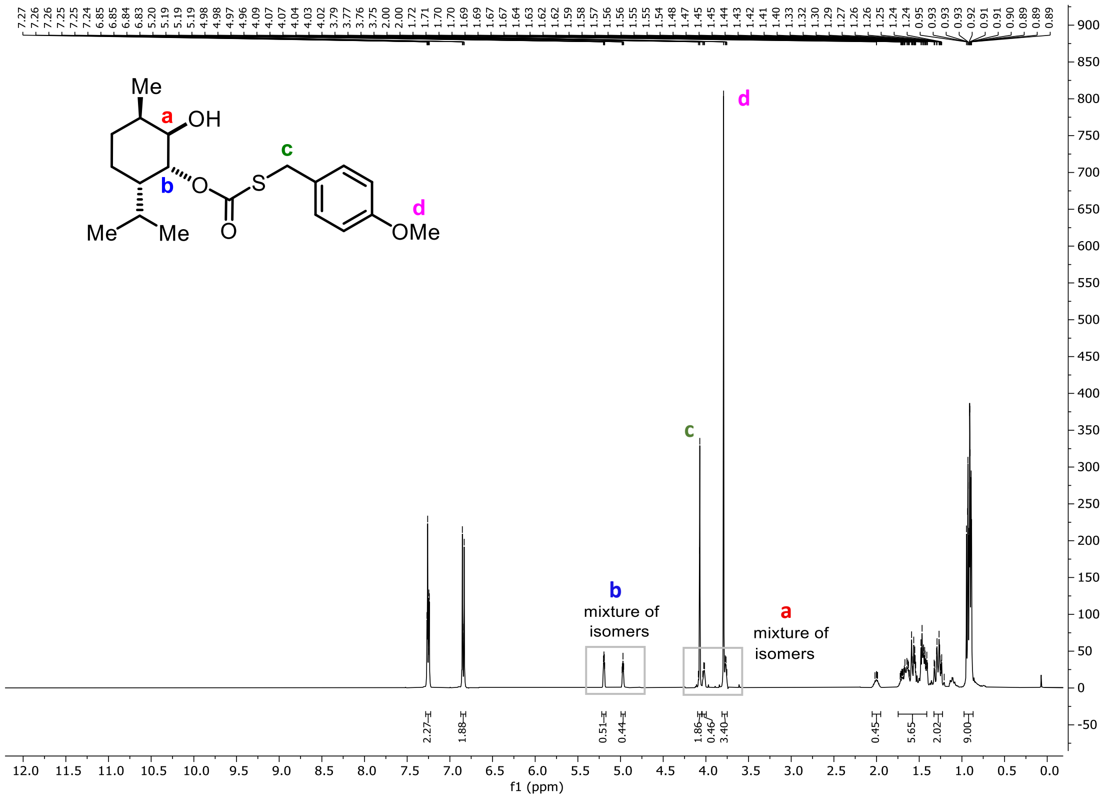

Figure S34. ^{1}H NMR spectrum ($CDCl_3$, 400 MHz) of compound **7**.

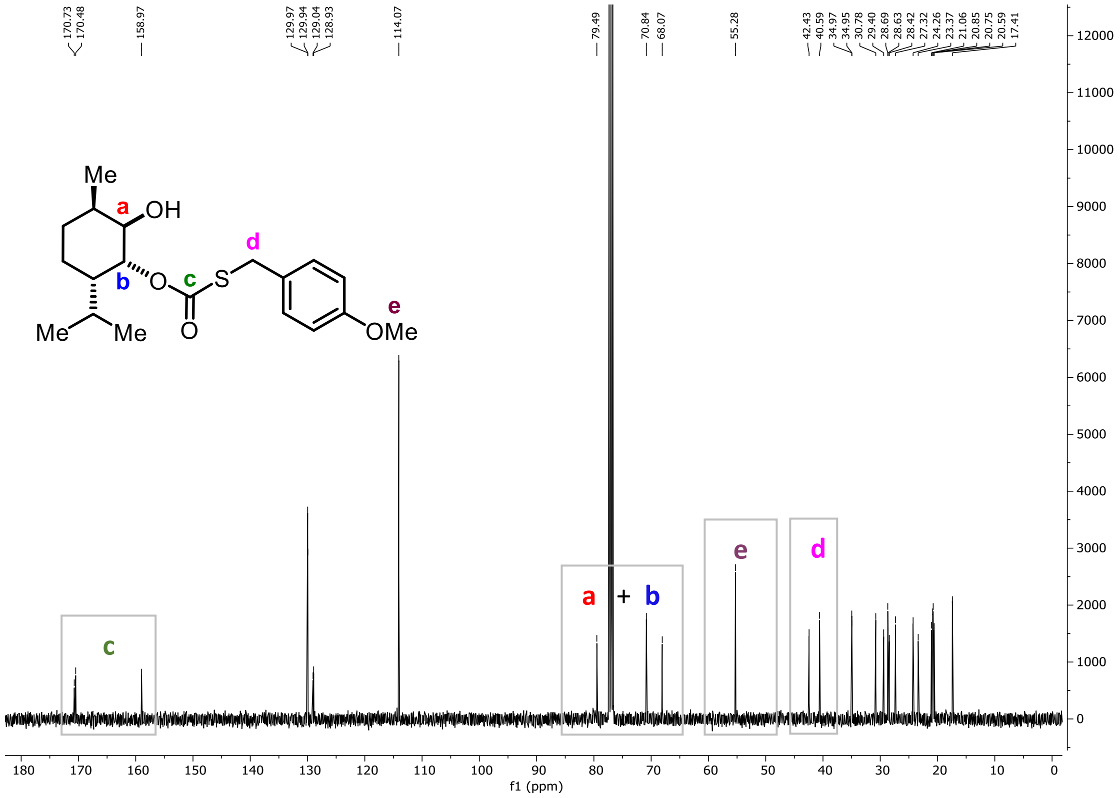

Figure S35. ^{13}C NMR spectrum ($CDCl_3$, 101 MHz) of compound **7**.

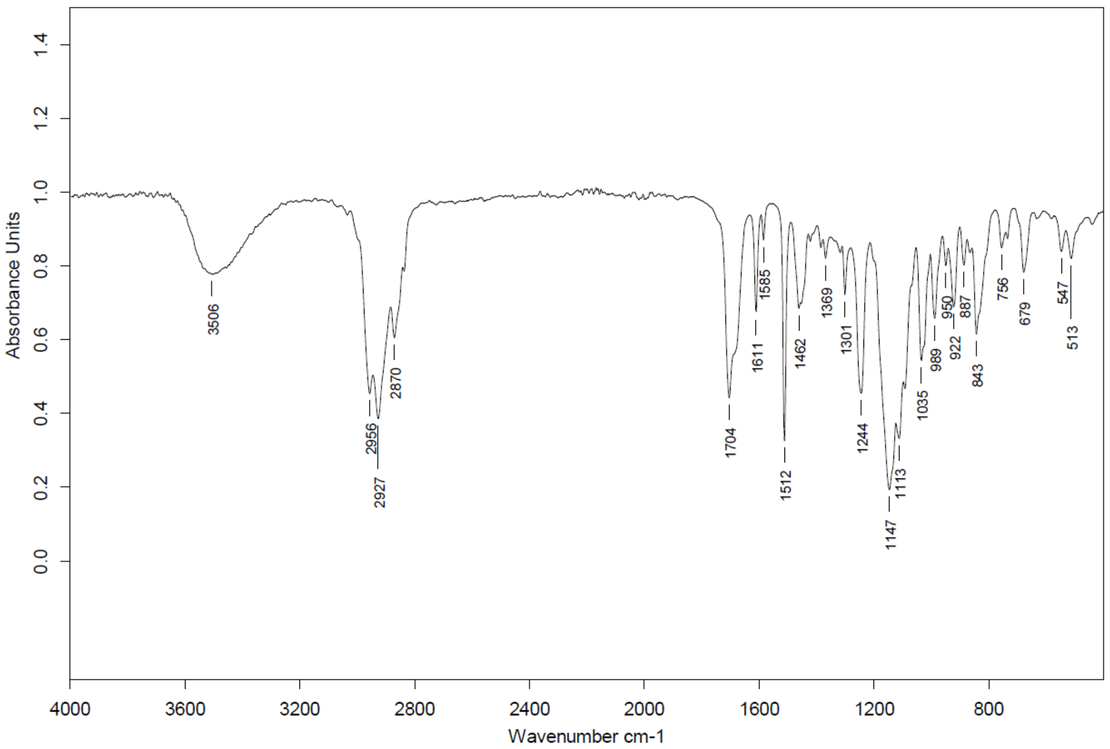

Figure S36. IR spectrum (neat) of compound **7**.

Analytical data for compound **8**

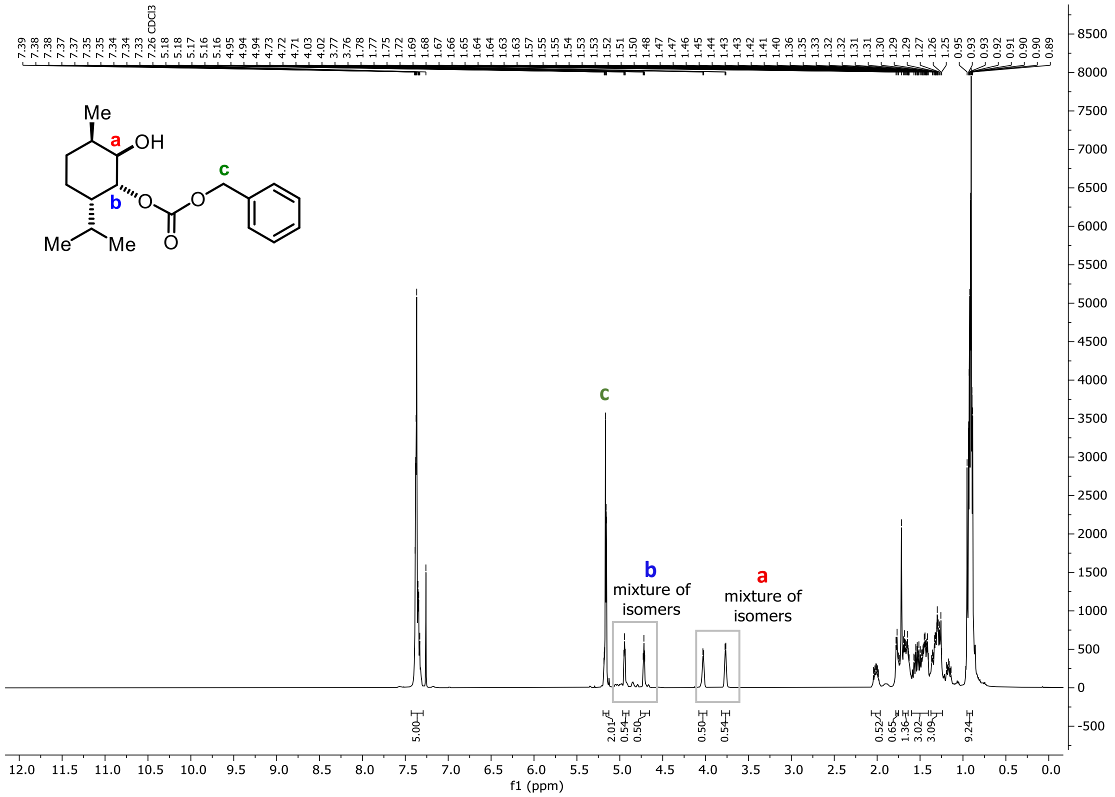

Figure S37. ^{1}H NMR spectrum ($CDCl_3$, 400 MHz) of compound **8**.

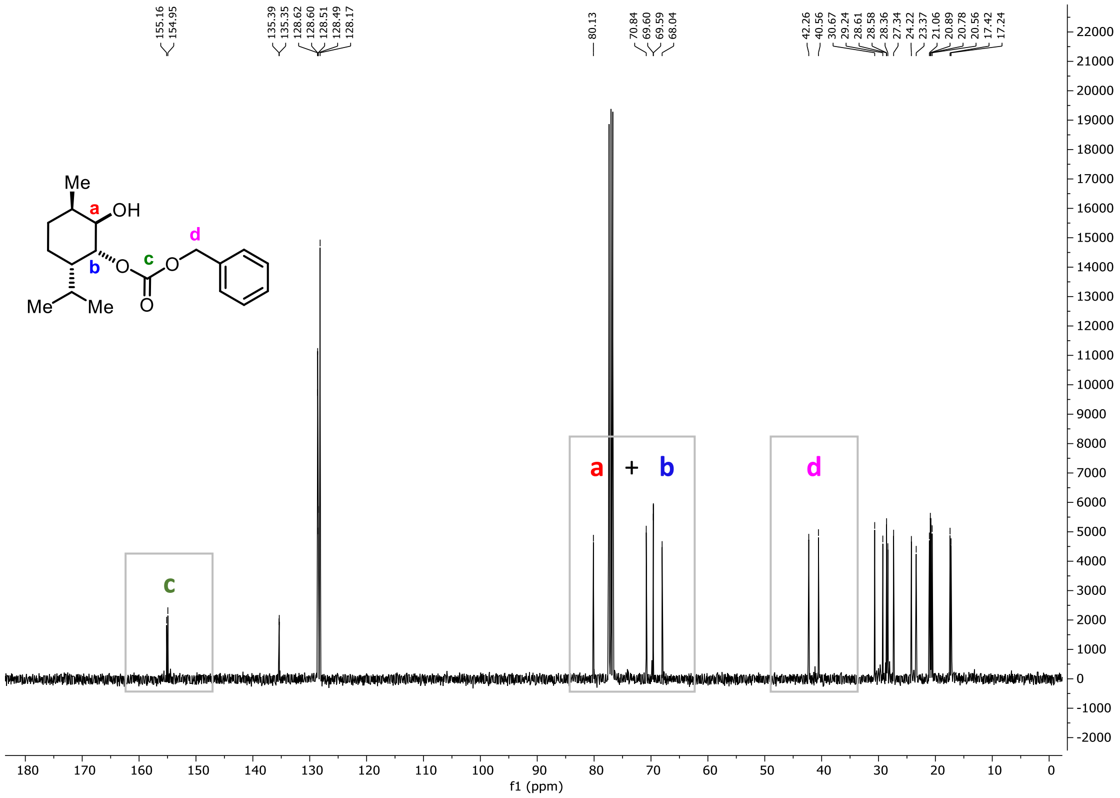

Figure S38. ^{13}C NMR spectrum ($CDCl_3$, 101 MHz) of compound **8**.

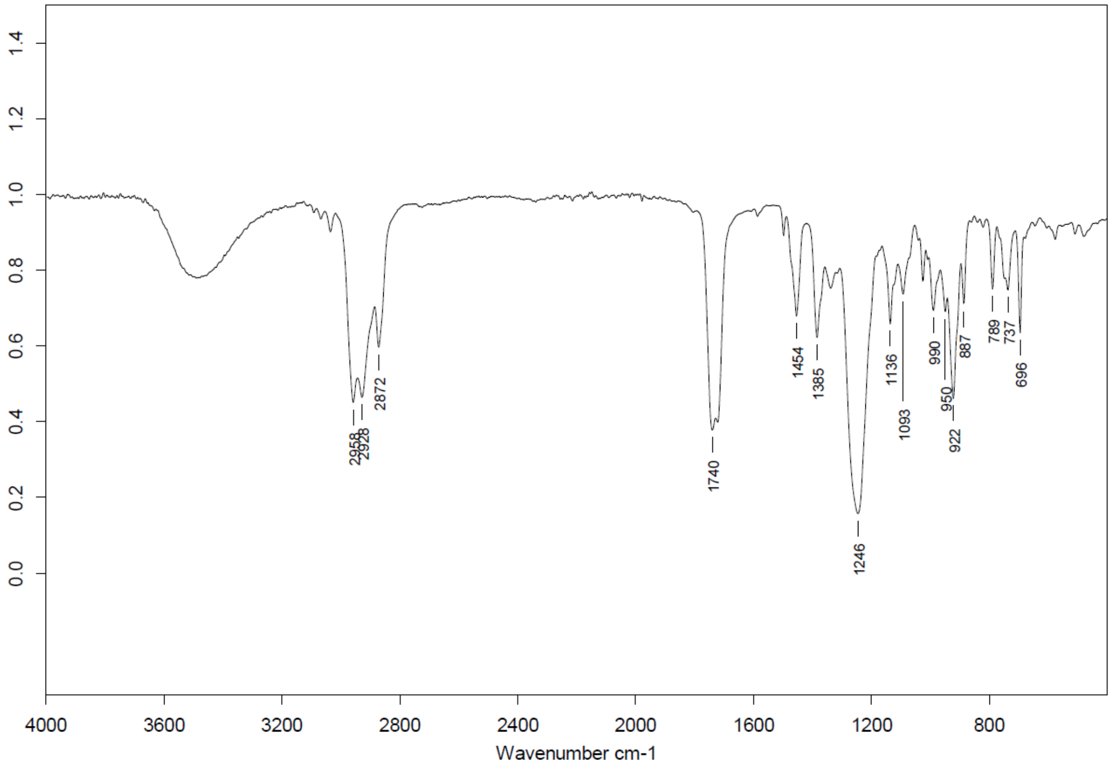

Figure S39. IR spectrum (neat) of compound **8**.

Analytical data for compound **9**

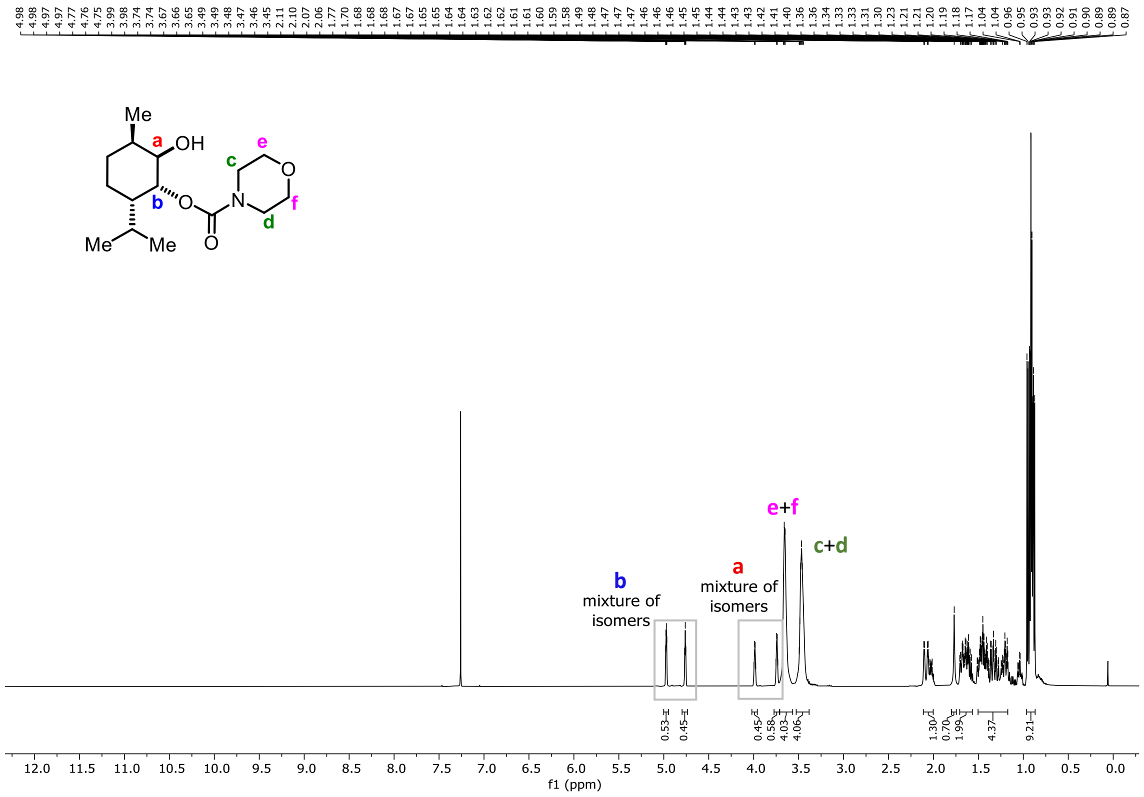

Figure S40. ^{1}H NMR spectrum ($CDCl_3$, 500 MHz) of compound **9**.

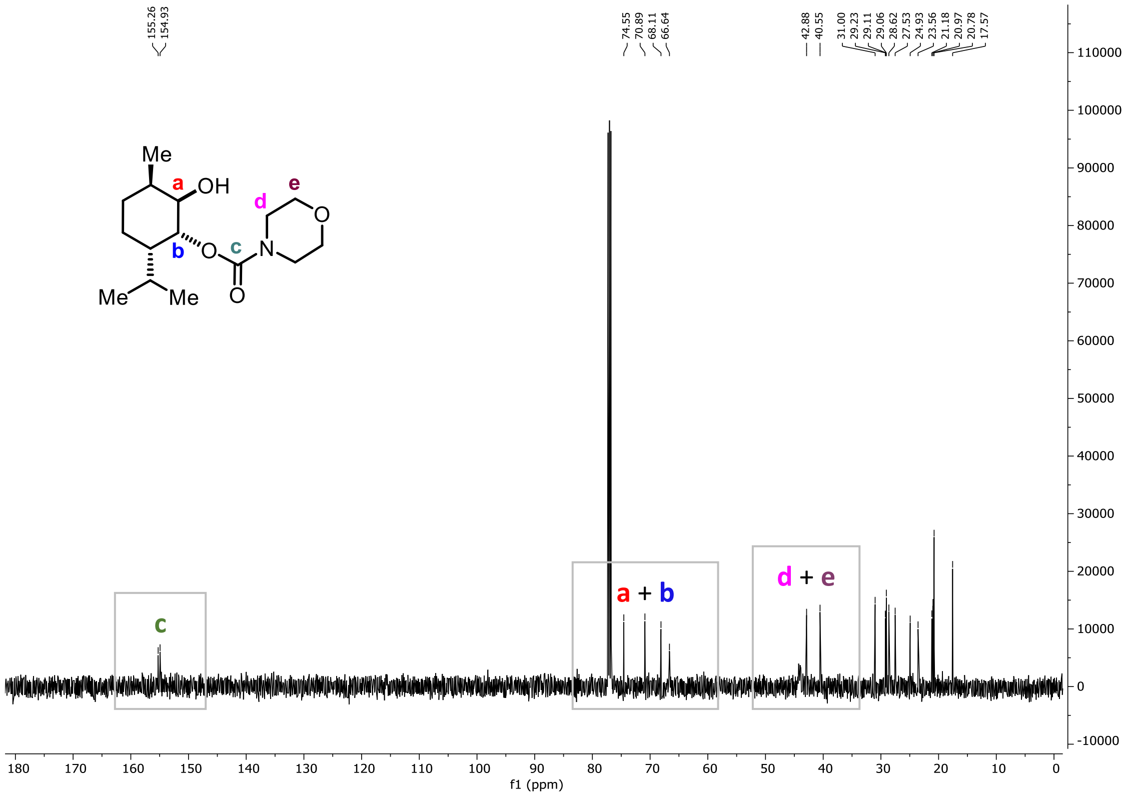

Figure S41. ^{13}C NMR spectrum ($CDCl_3$, 126 MHz) of compound **9**.

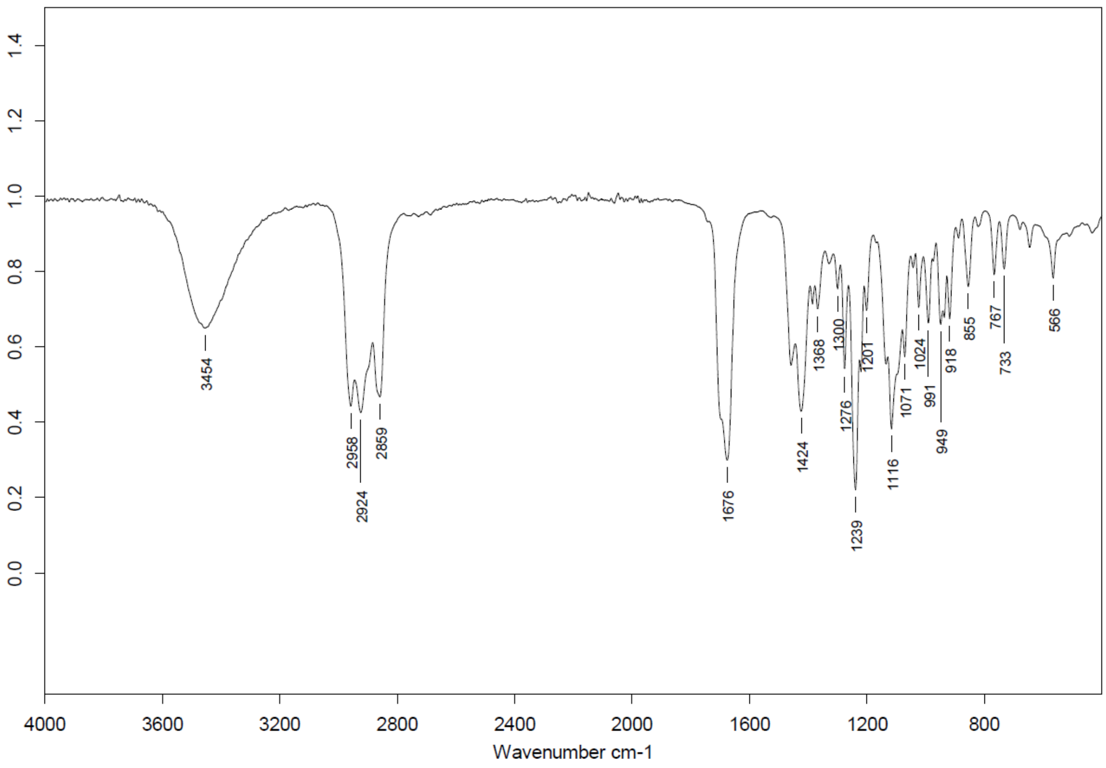

Figure S42. IR spectrum (neat) of compound **9**.

Analytical data for compound **10**

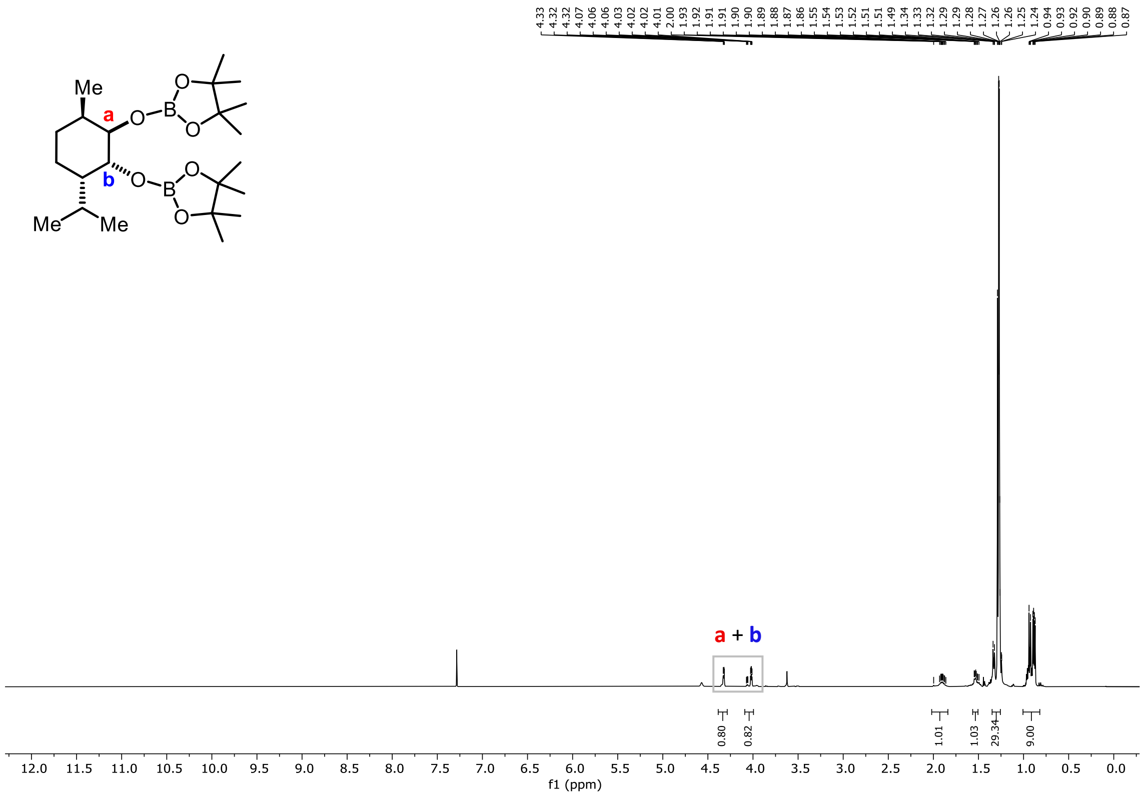

Figure S43. ^{1}H NMR spectrum ($CDCl_3$, 400 MHz) of compound **10**.

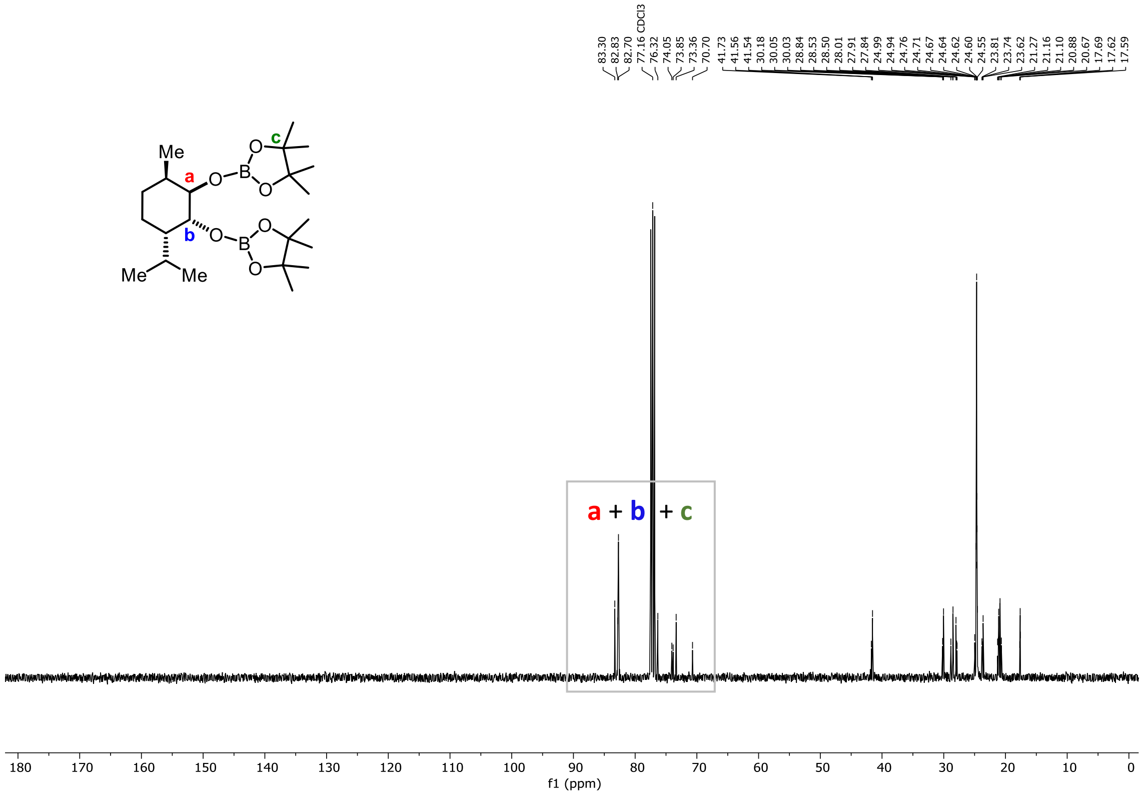

Figure S44. ^{13}C NMR spectrum ($CDCl_3$, 101 MHz) of compound **10**.

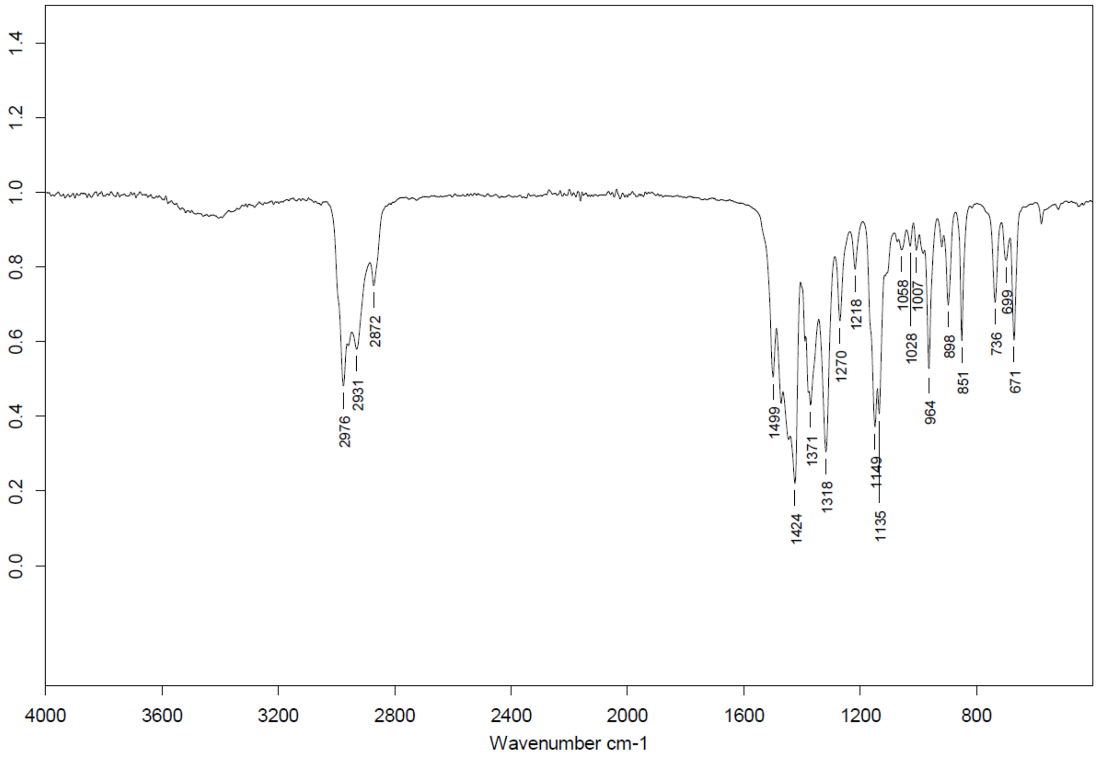

Figure S45. IR spectrum of compound **10**.

Analytical data for compound **12**

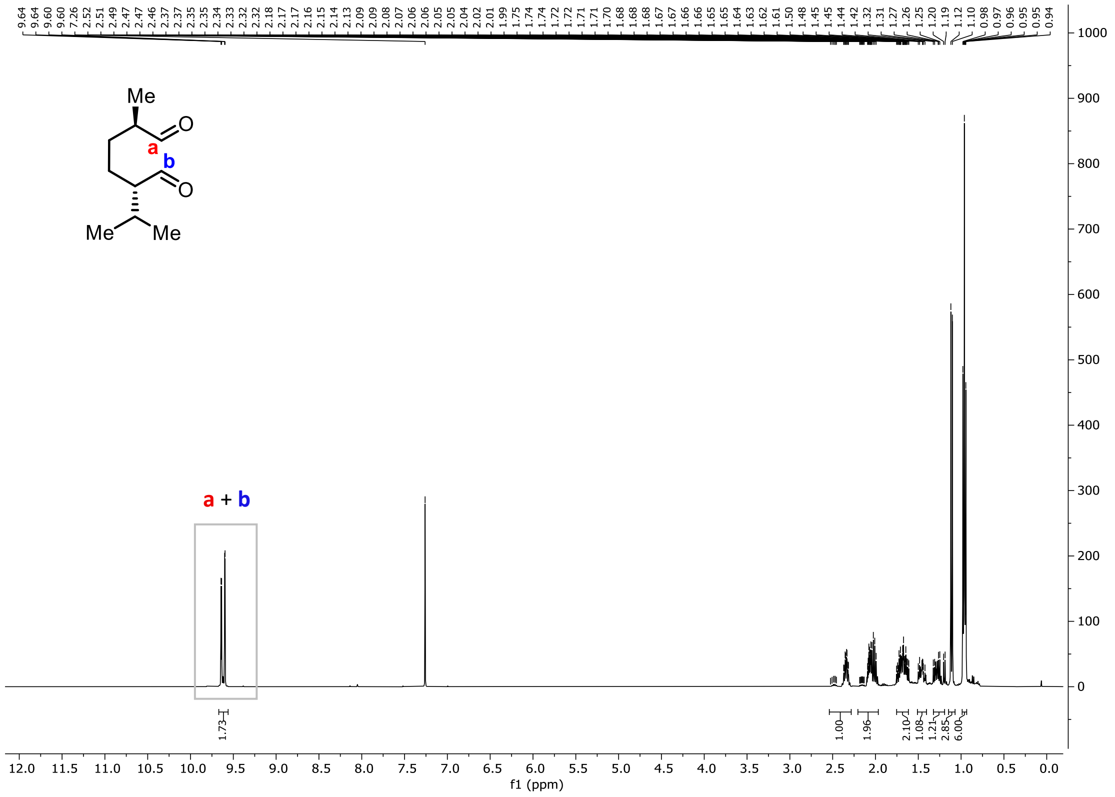

Figure S46. ^{1}H NMR spectrum ($CDCl_3$, 400 MHz) of compound **12**.

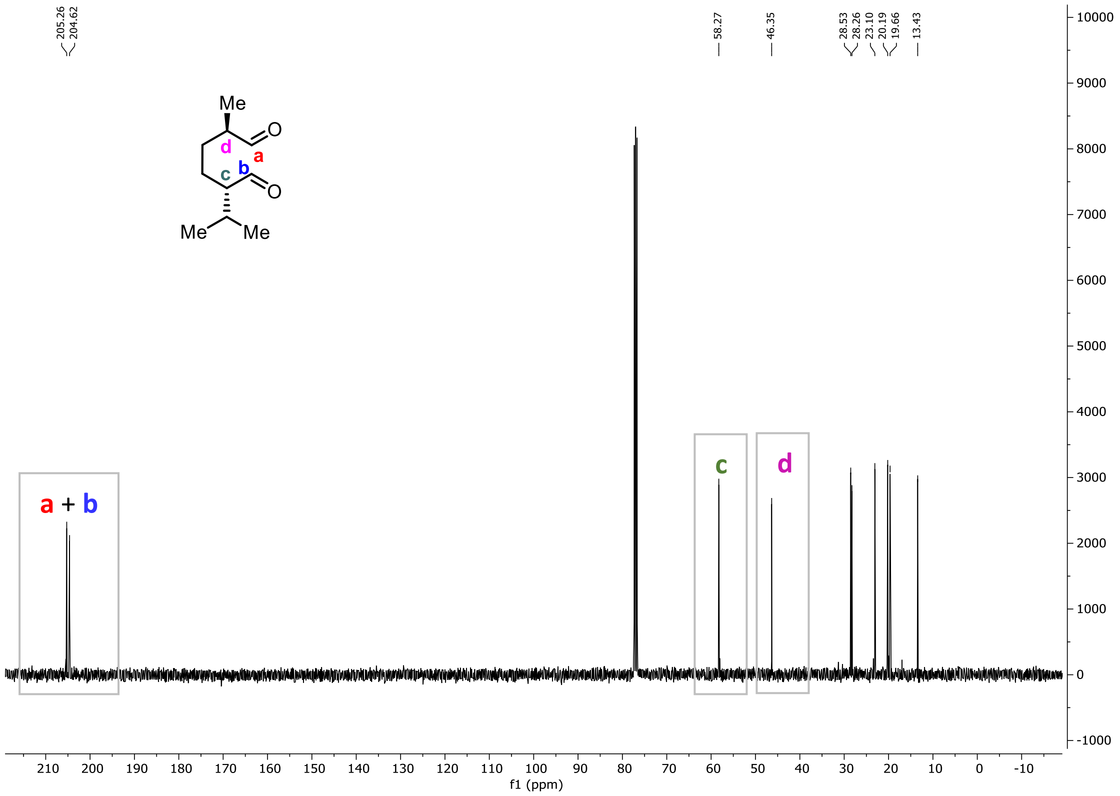

Figure S47. ^{13}C NMR spectrum ($CDCl_3$, 101 MHz) of compound **12**.

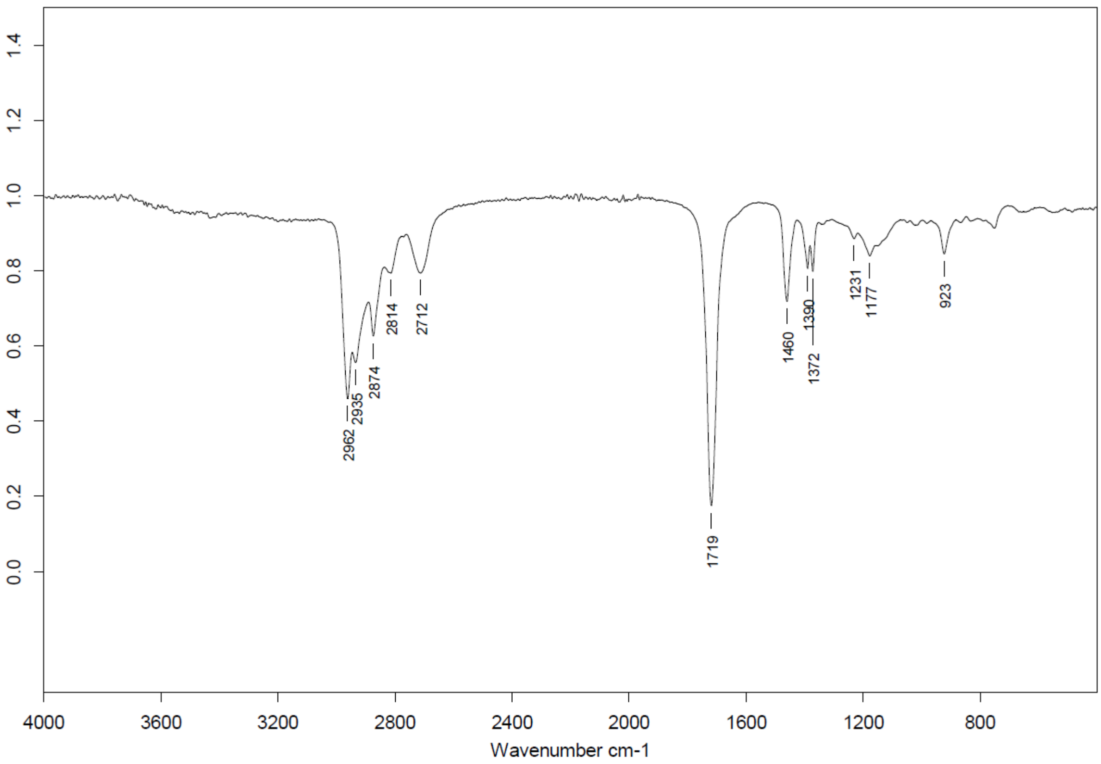

Figure S48. IR spectrum of compound **12**.

Analytical data for compound **13**

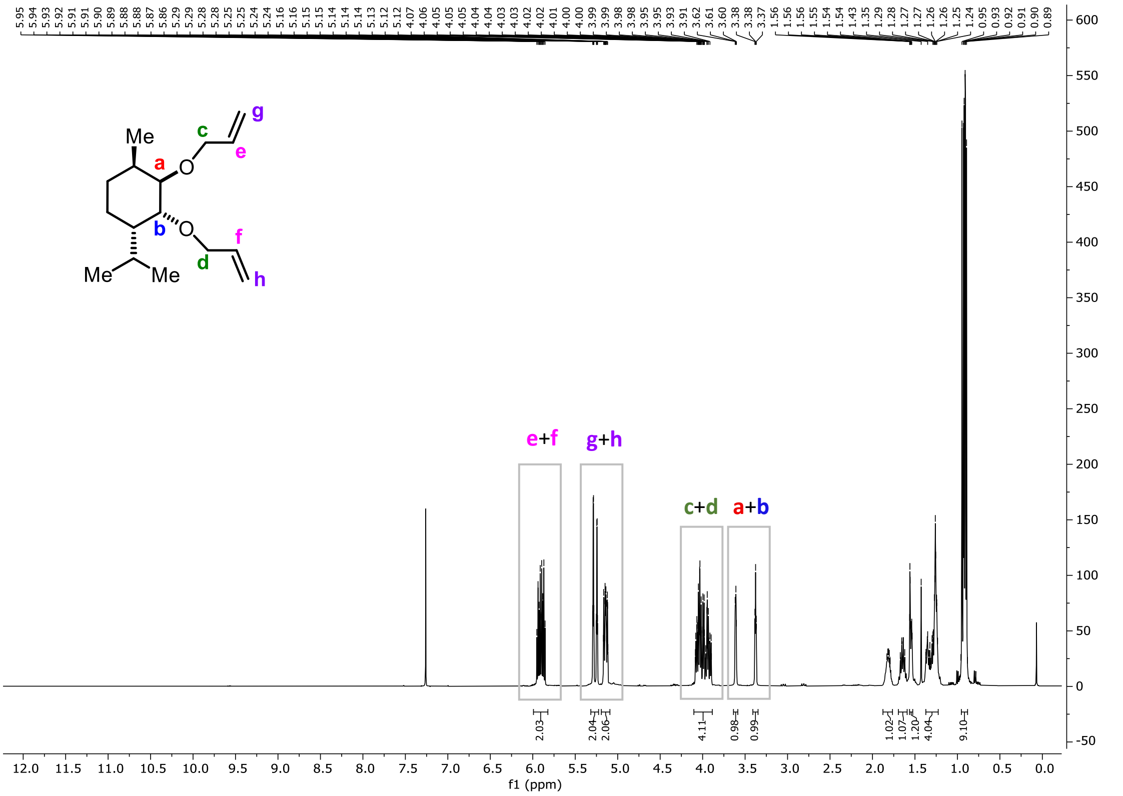

Figure S49. ^{1}H NMR spectrum ($CDCl_3$, 400 MHz) of compound **13**.

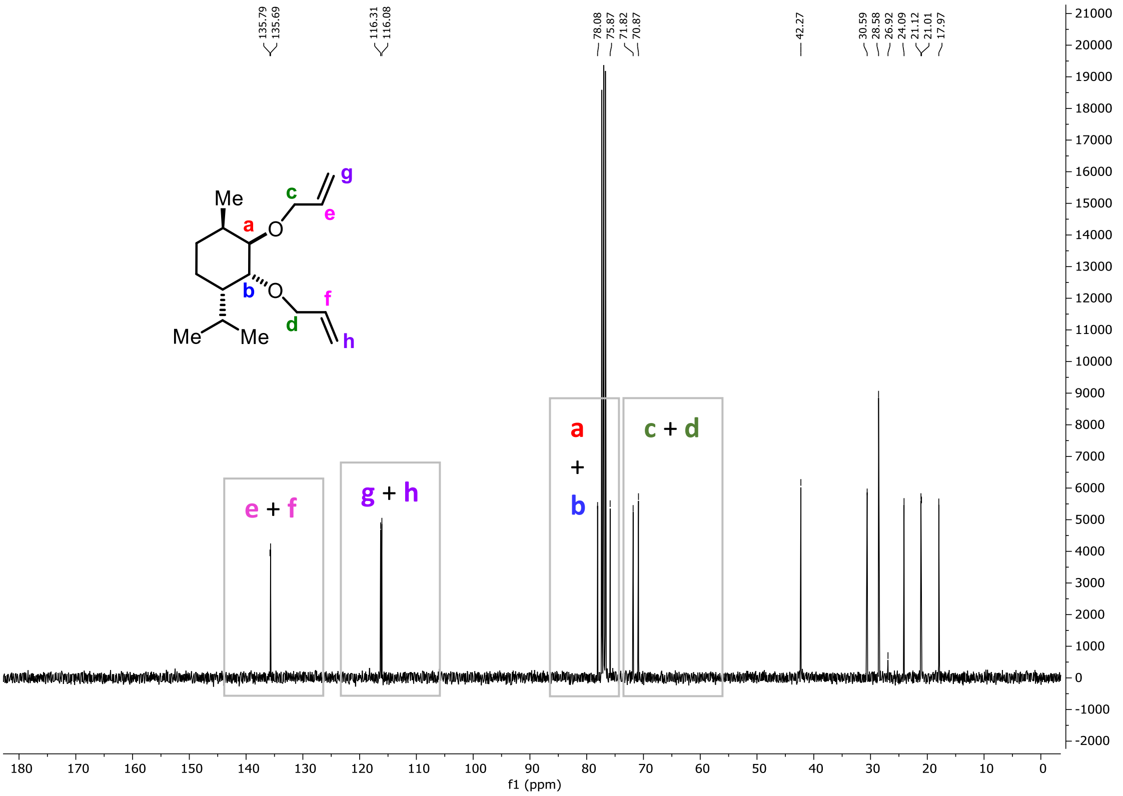

Figure S50. ^{13}C NMR spectrum ($CDCl_3$, 101 MHz) of compound **13**.

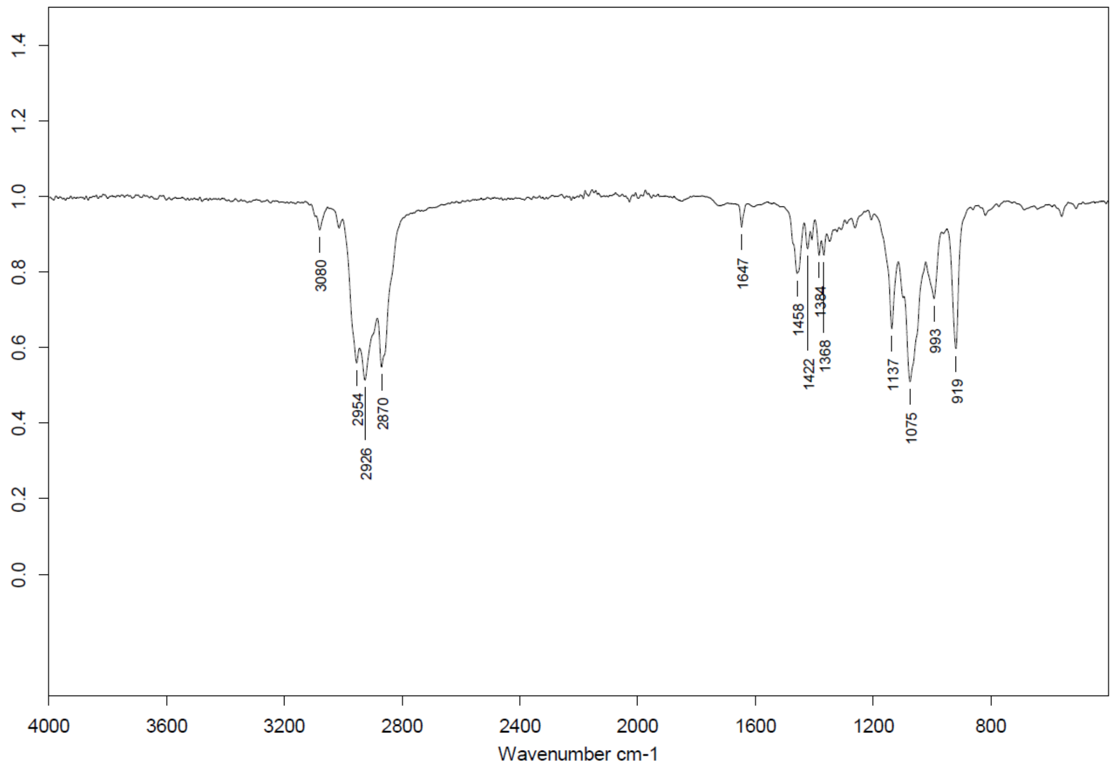

Figure S51. IR spectrum of compound **13**.

Analytical data for compound **14**

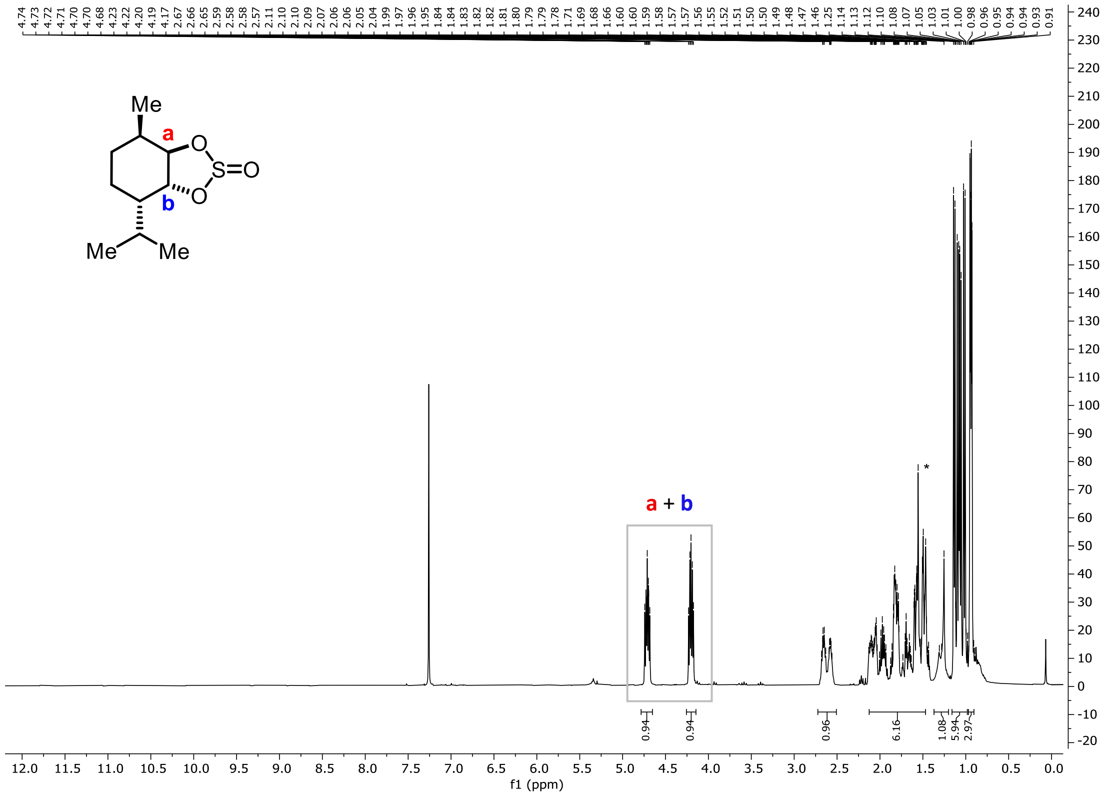

Figure S52. ^{1}H NMR spectrum ($CDCl_3$, 400 MHz) of compound **14**.

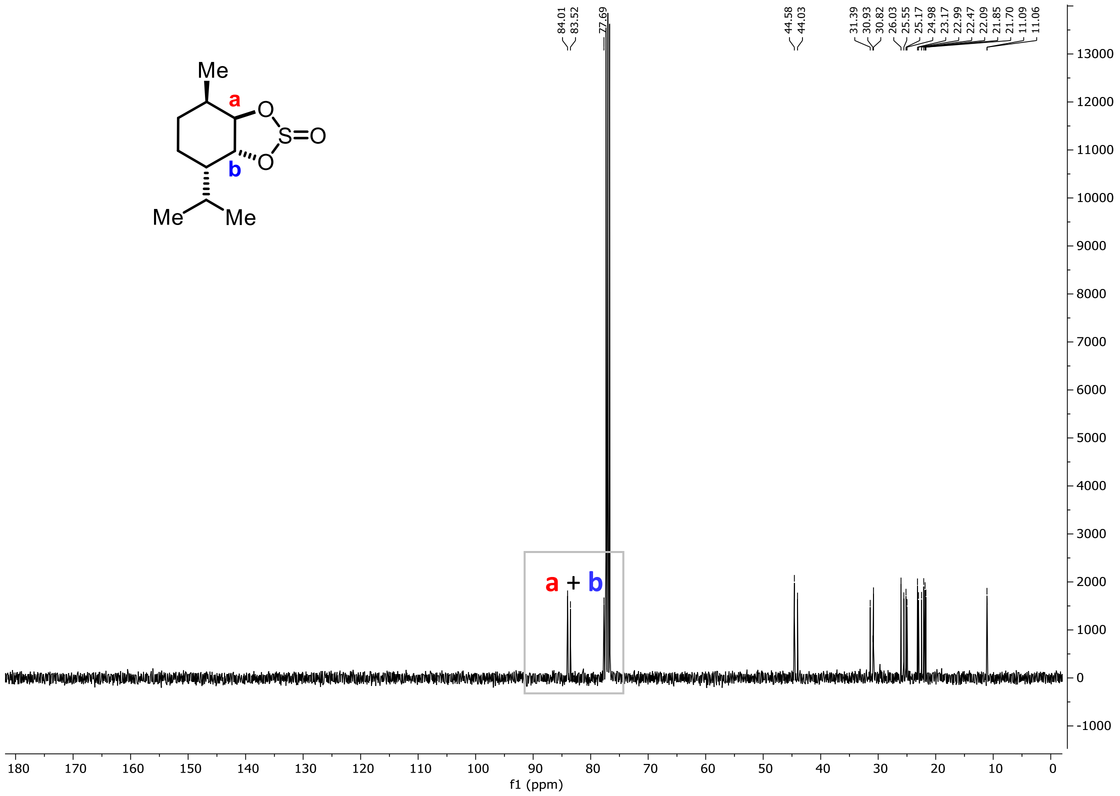

Figure S53. ^{13}C NMR spectrum ($CDCl_3$, 101 MHz) of compound **14**.

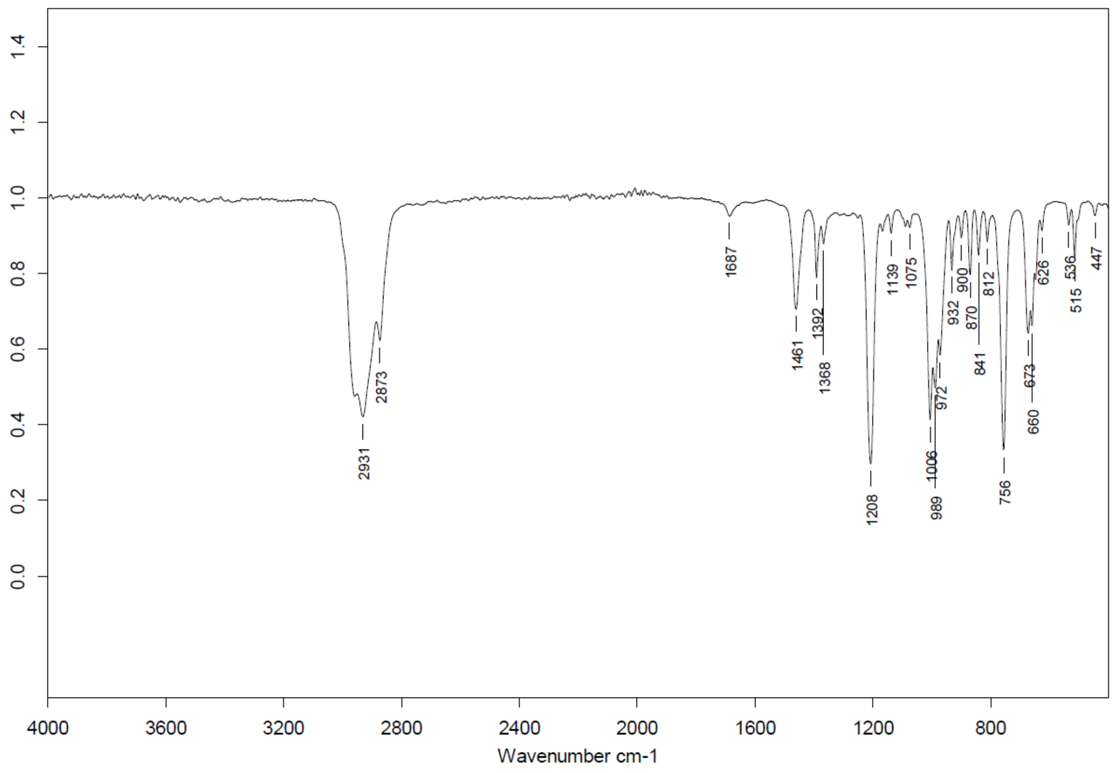

Figure S54. IR spectrum of compound **14**.

Analytical data for compound **15**

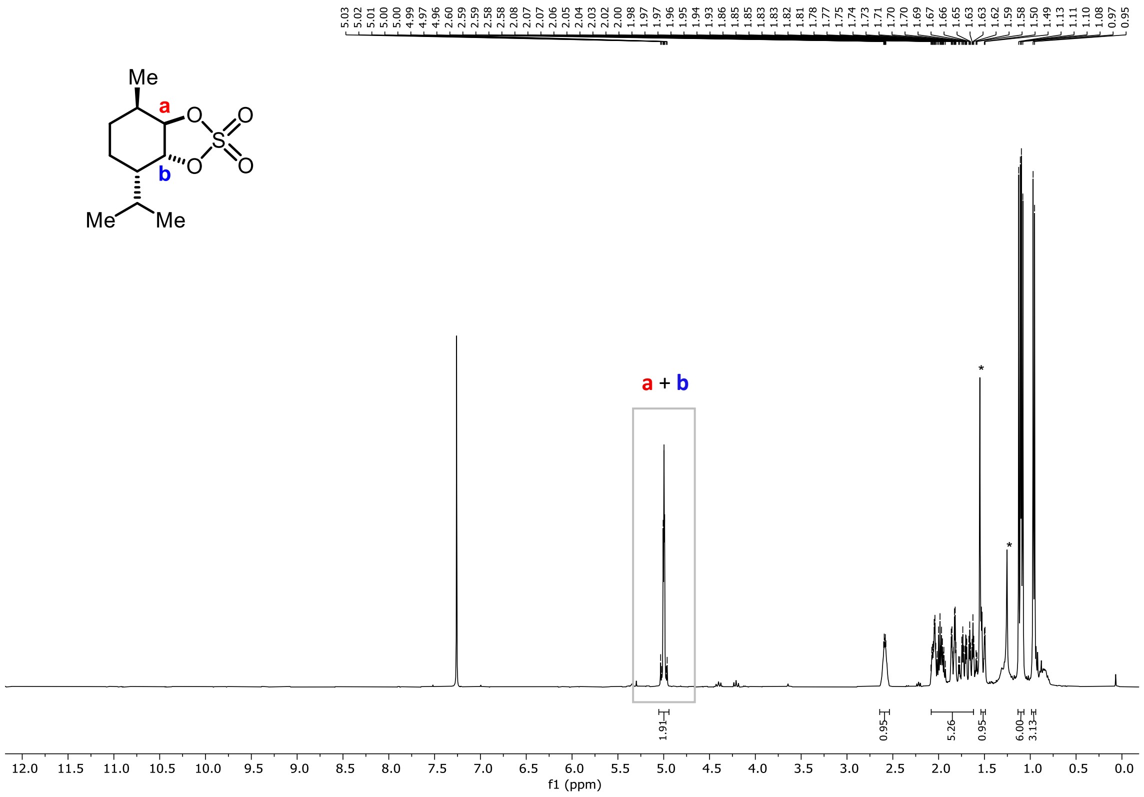

Figure S55. ^{1}H NMR spectrum ($CDCl_3$, 400 MHz) of compound **15**.

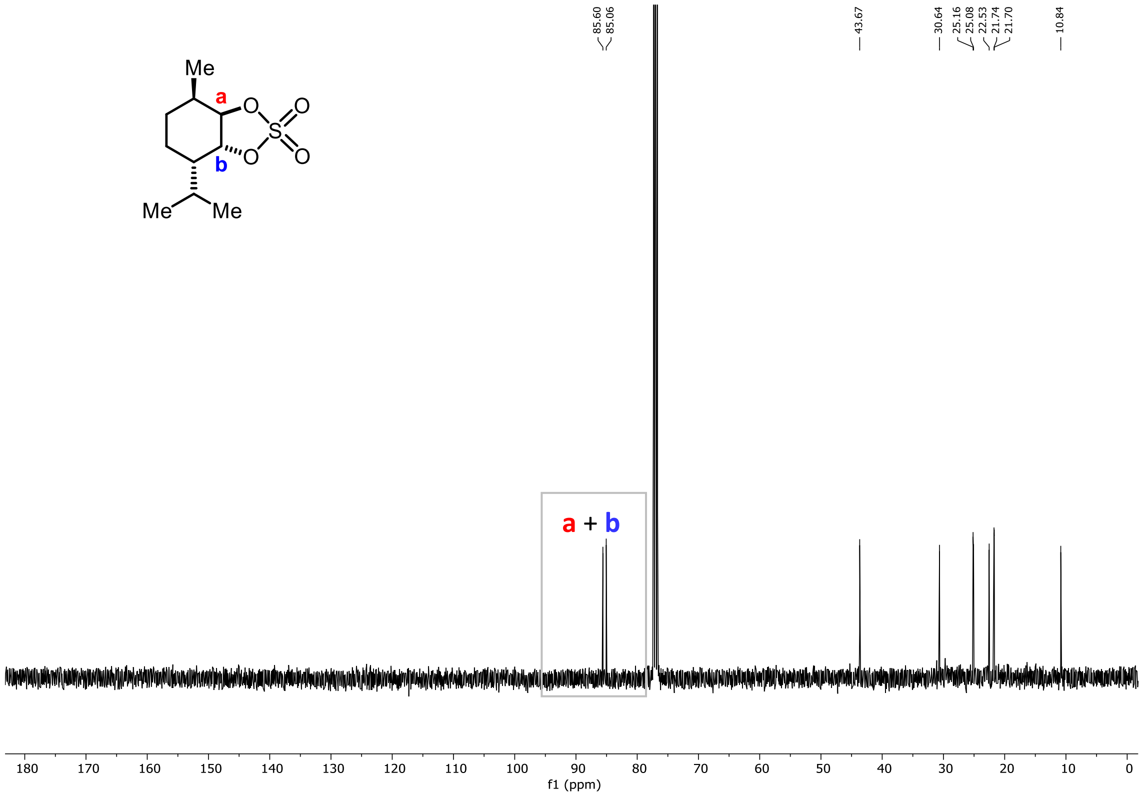

Figure S56. ^{13}C NMR spectrum ($CDCl_3$, 101 MHz) of compound **15**.

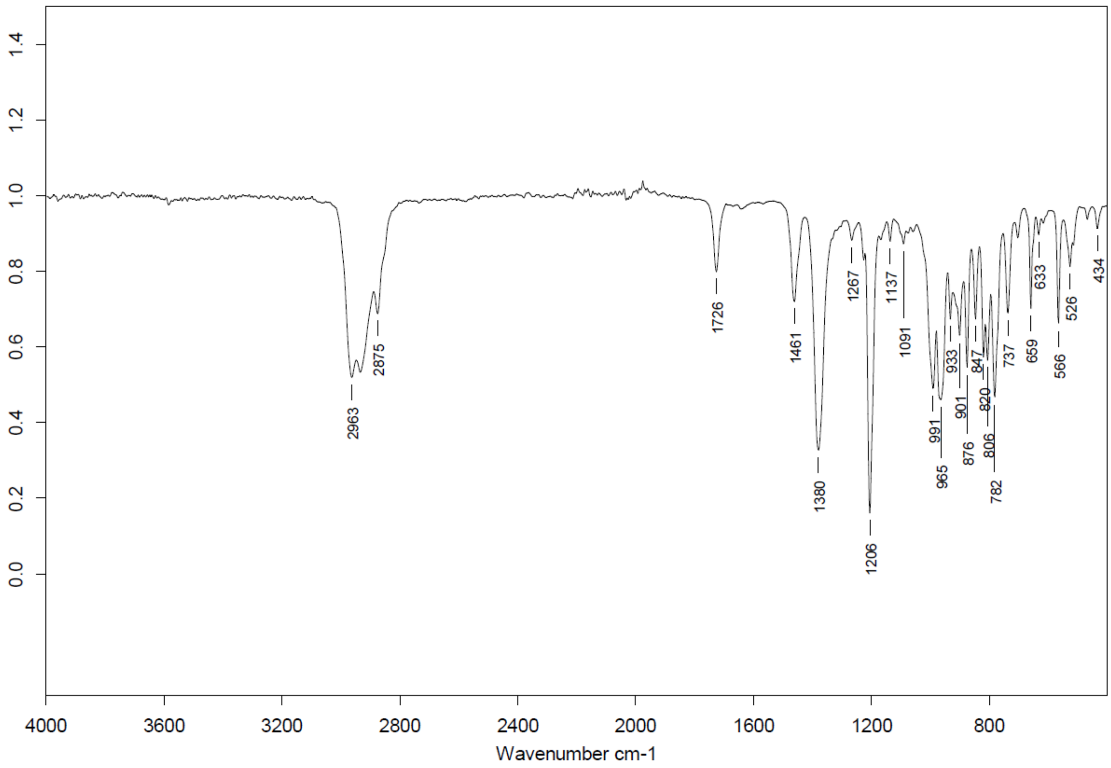

Figure S57. IR spectrum of compound **15**.

Analytical data for compound **16**

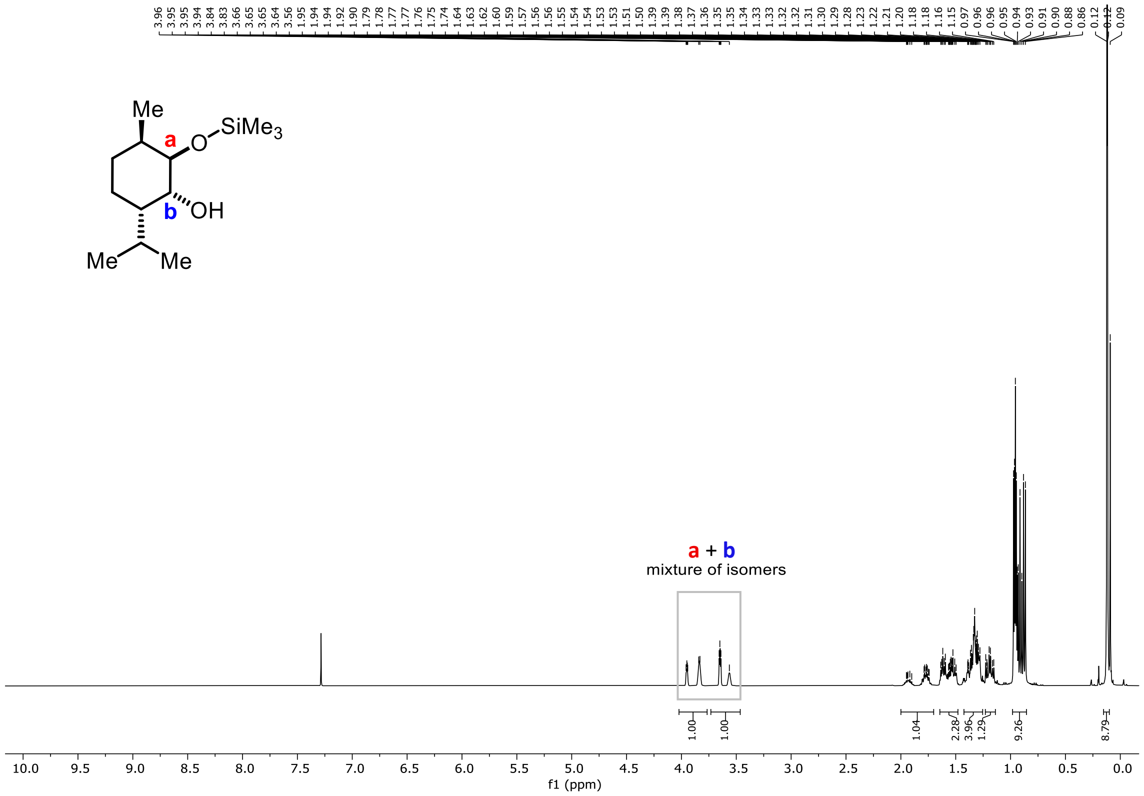

Figure S58. ^{1}H NMR spectrum ($CDCl_3$, 400 MHz) of compound **16**.

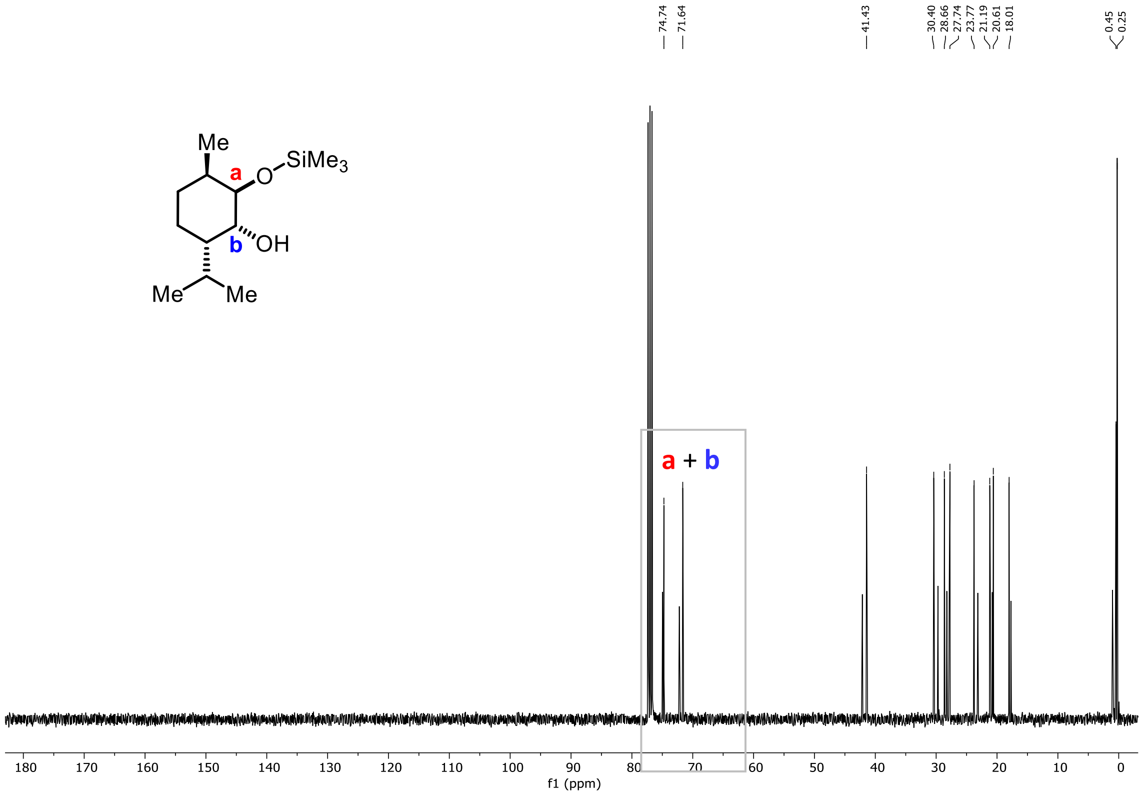

Figure S59. ^{13}C NMR spectrum ($CDCl_3$, 101 MHz) of compound **16**.

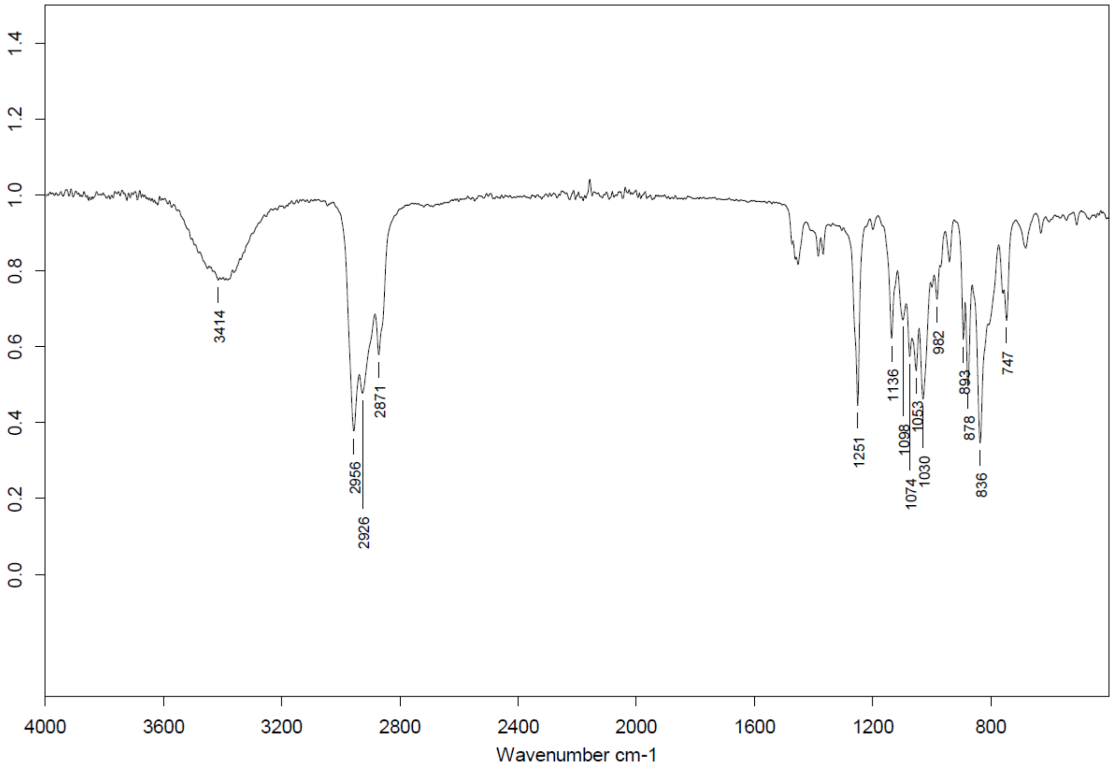

Figure S60. IR spectrum of compound **16**.

Analytical data for compound **17**

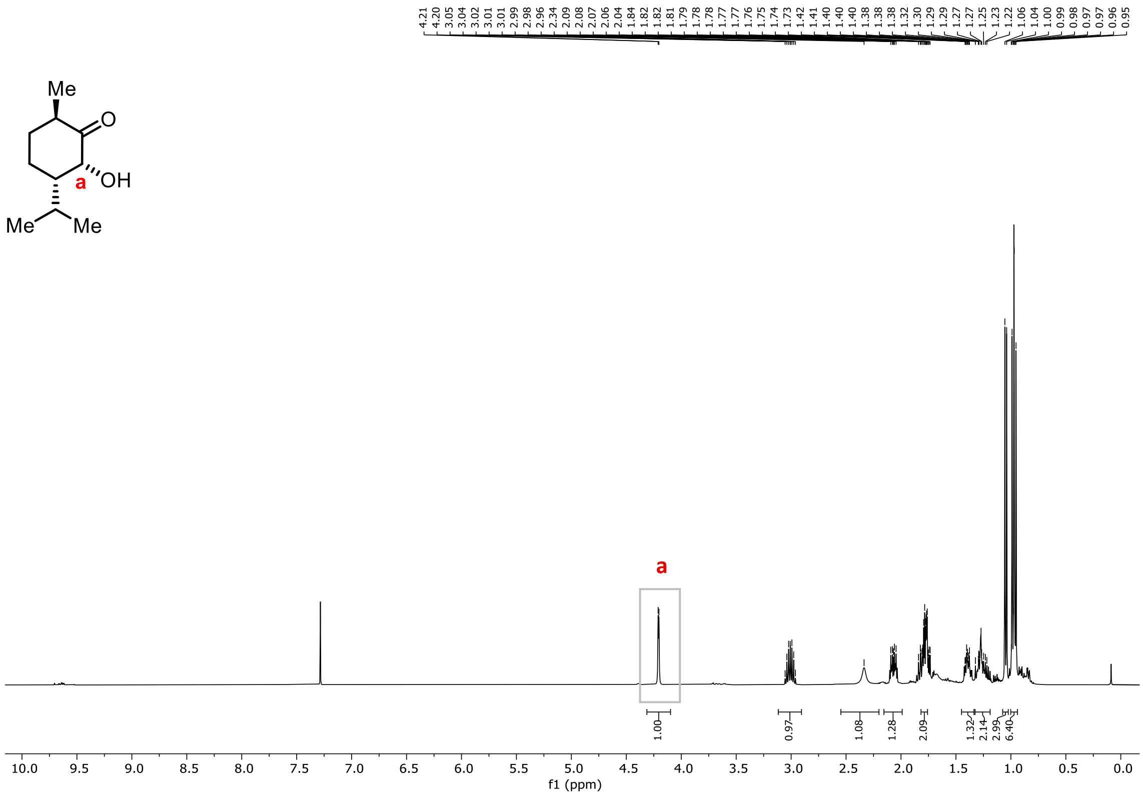

Figure S61. ^{1}H NMR spectrum ($CDCl_3$, 400 MHz) of compound **17**.

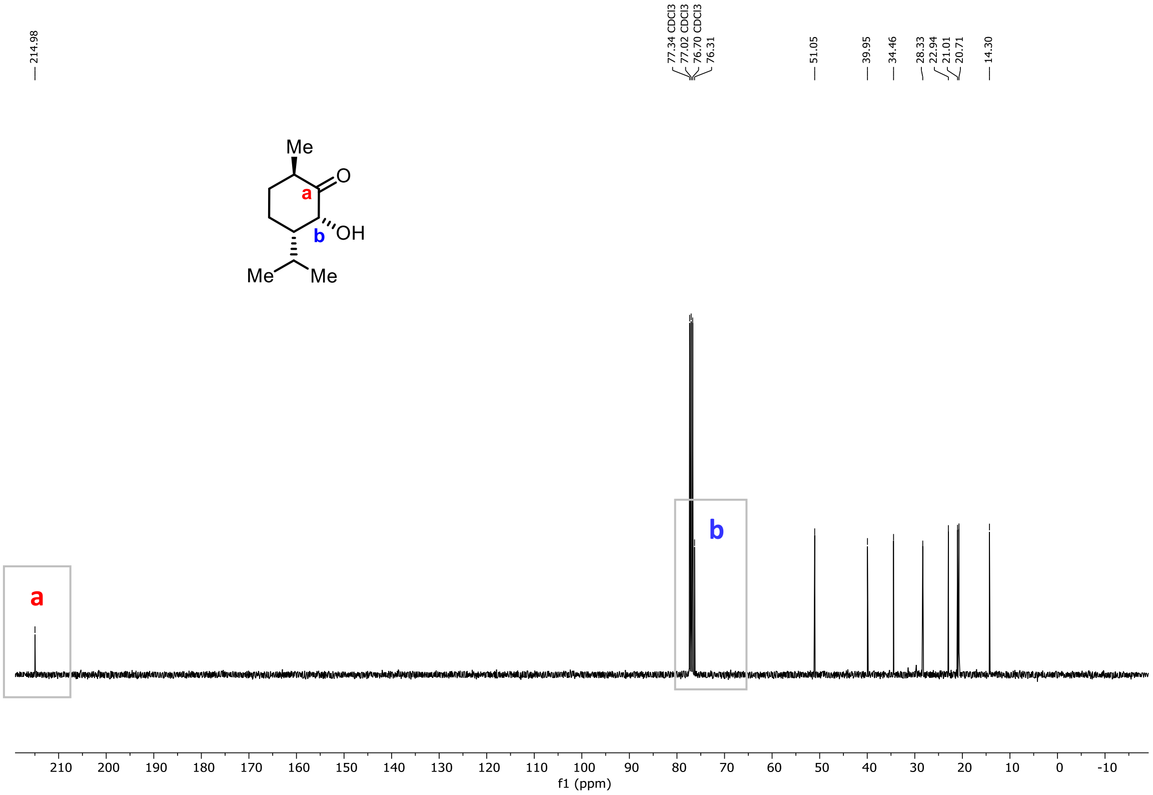

Figure S62. ^{13}C NMR spectrum ($CDCl_3$, 101 MHz) of compound **17**.

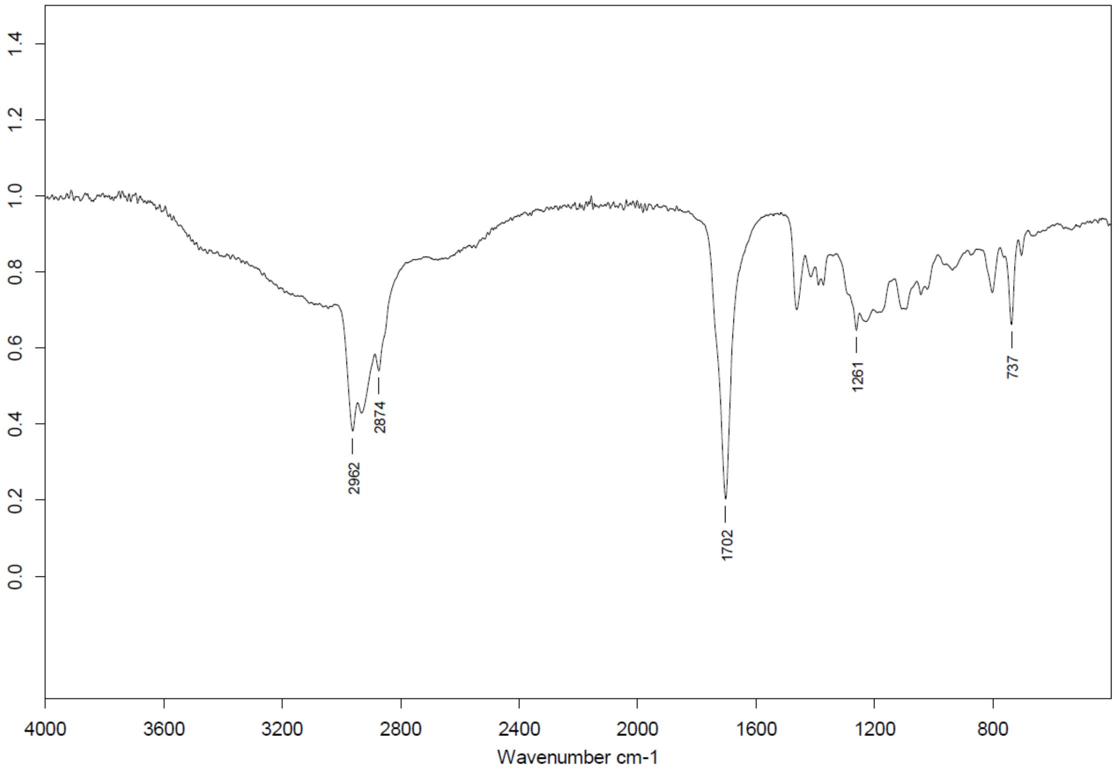

Figure S63. IR spectrum of compound **17**.

Analytical data for compound **18**

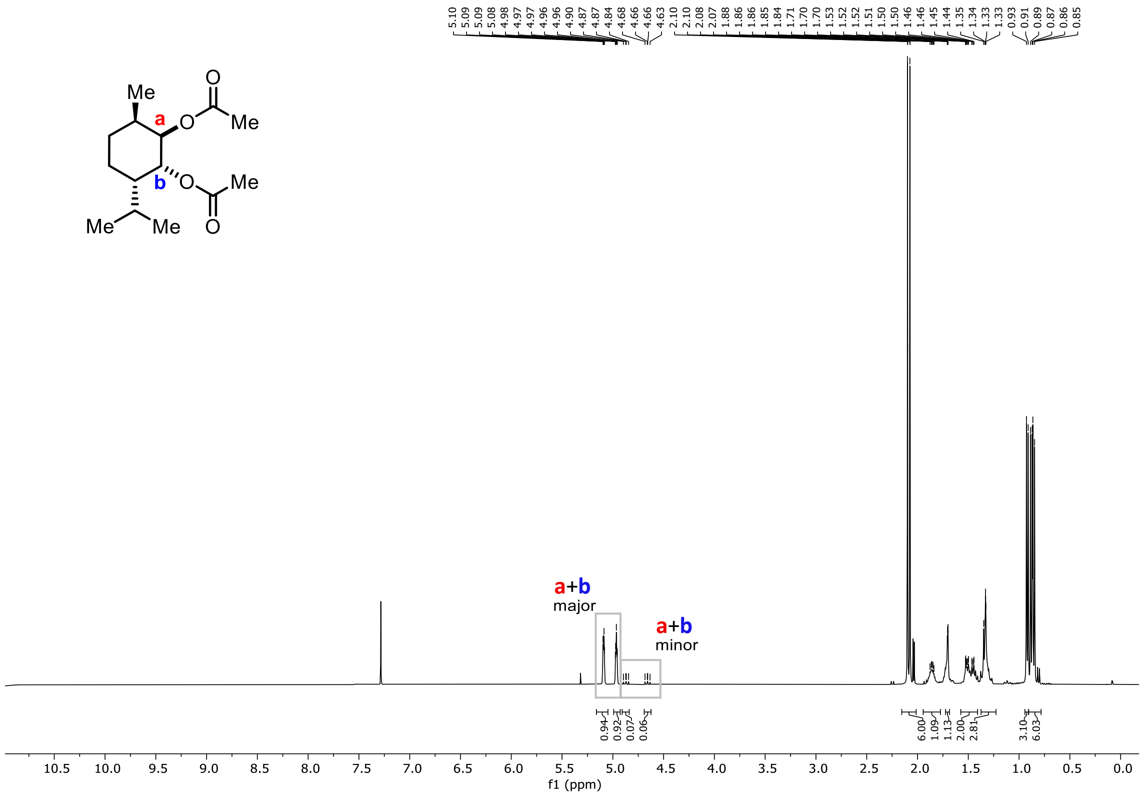

Figure S64. ^{1}H NMR spectrum ($CDCl_3$, 400 MHz) of compound **18**.

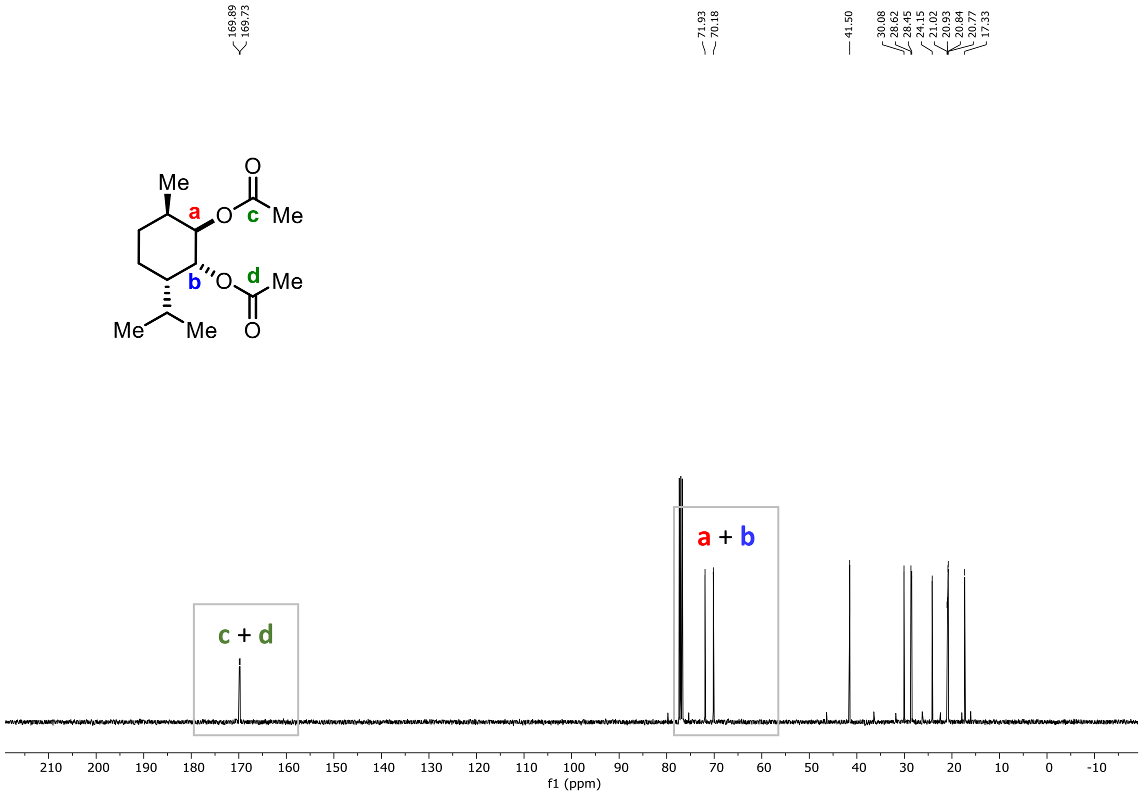

Figure S65. ^{13}C NMR spectrum ($CDCl_3$, 101 MHz) of compound **18**.

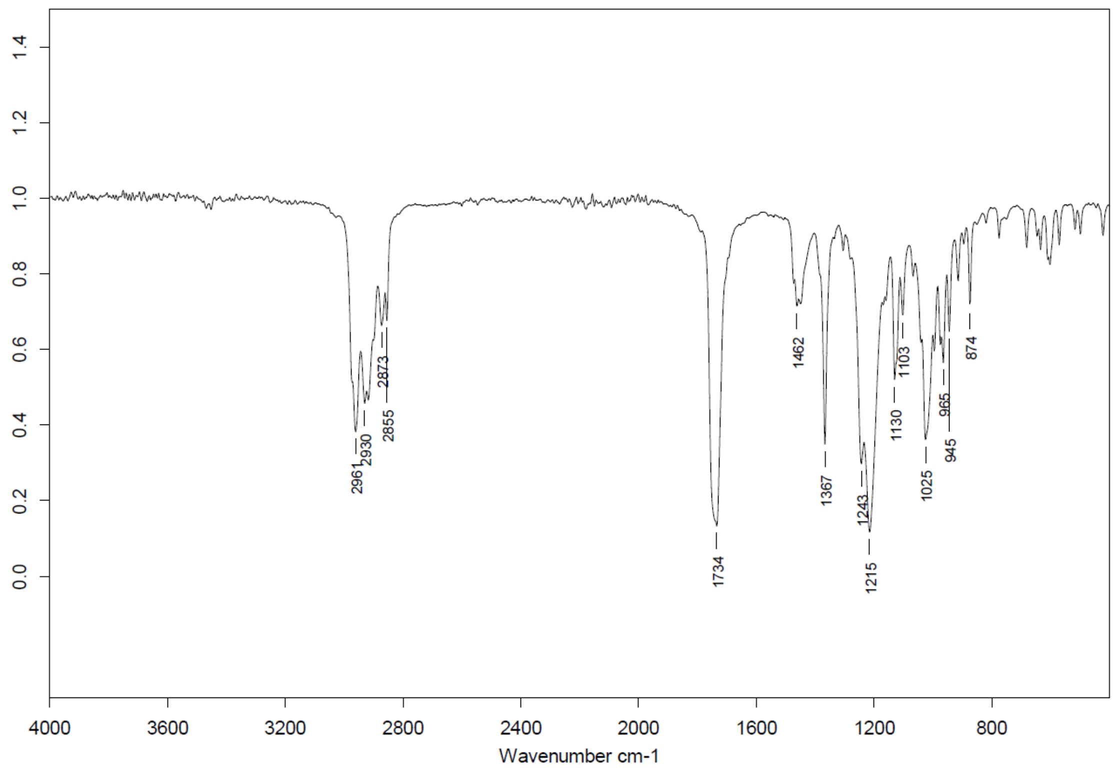

Figure S66. IR spectrum of compound **18**.

Analytical data for compound **19**

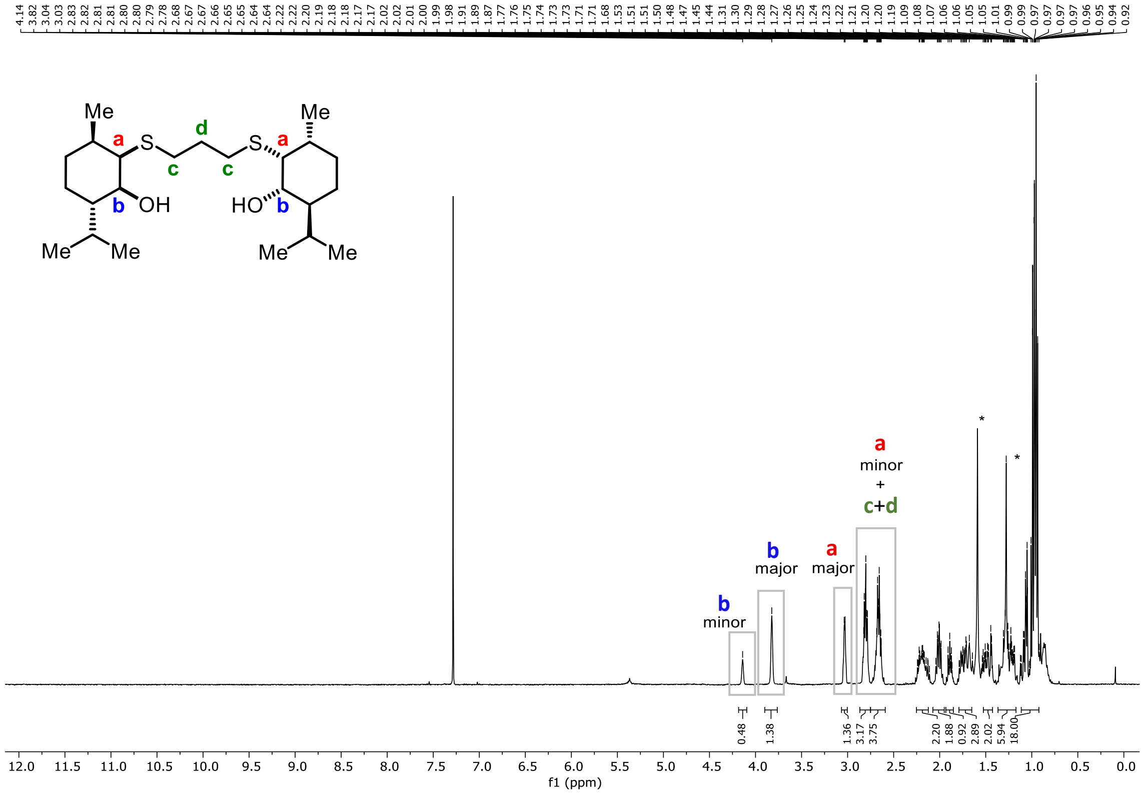

Figure S67. ^{1}H NMR spectrum ($CDCl_3$, 400 MHz) of compound **19**.

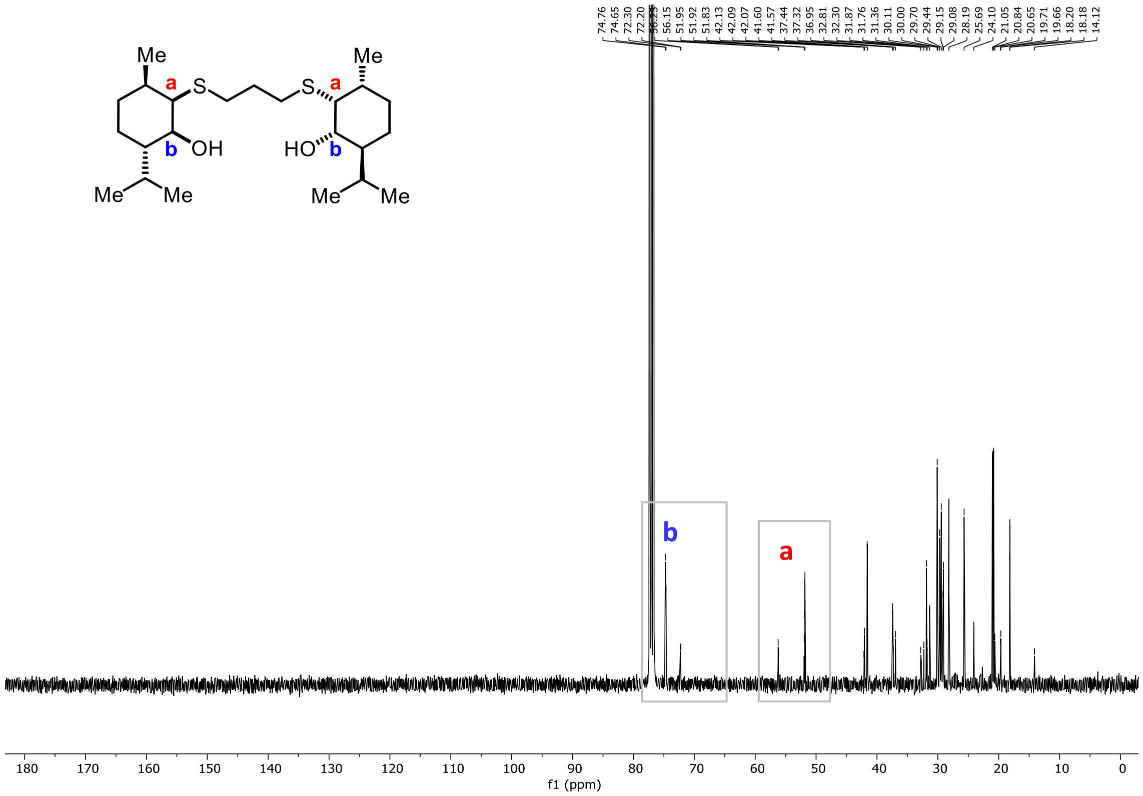

Figure S68. ^{13}C NMR spectrum ($CDCl_3$, 101 MHz) of compound **19**.

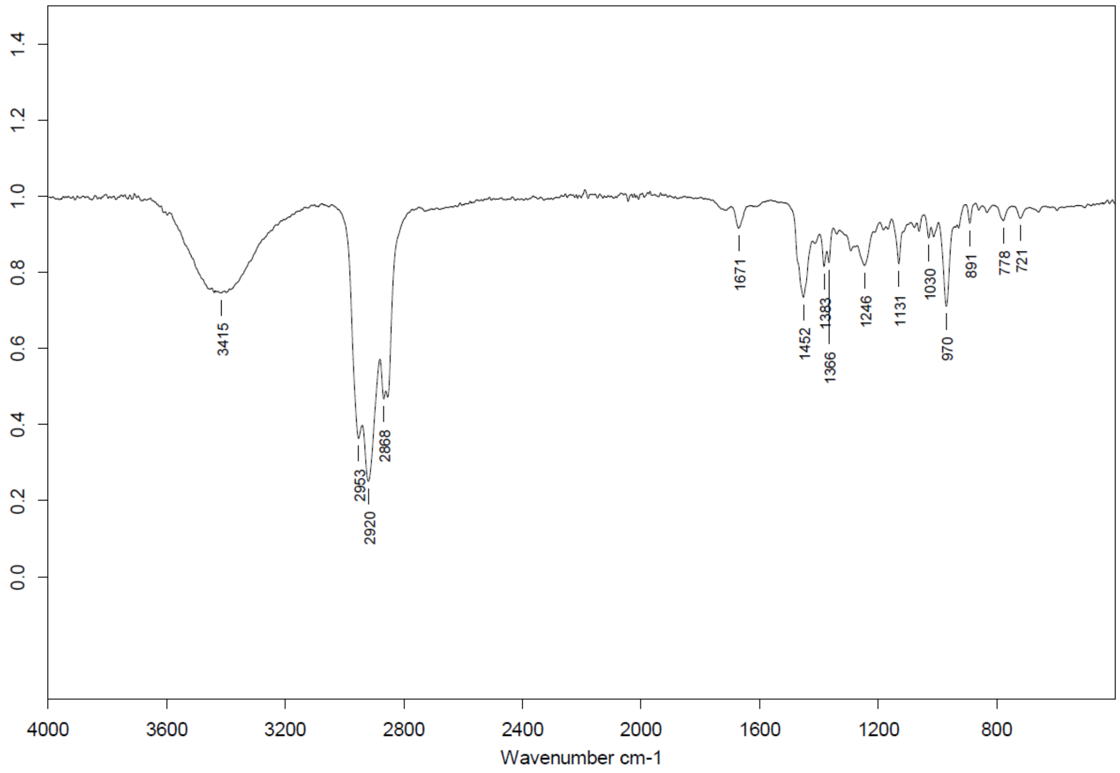

Figure S69. IR spectrum of compound **19**.

Analytical data for compound **20**

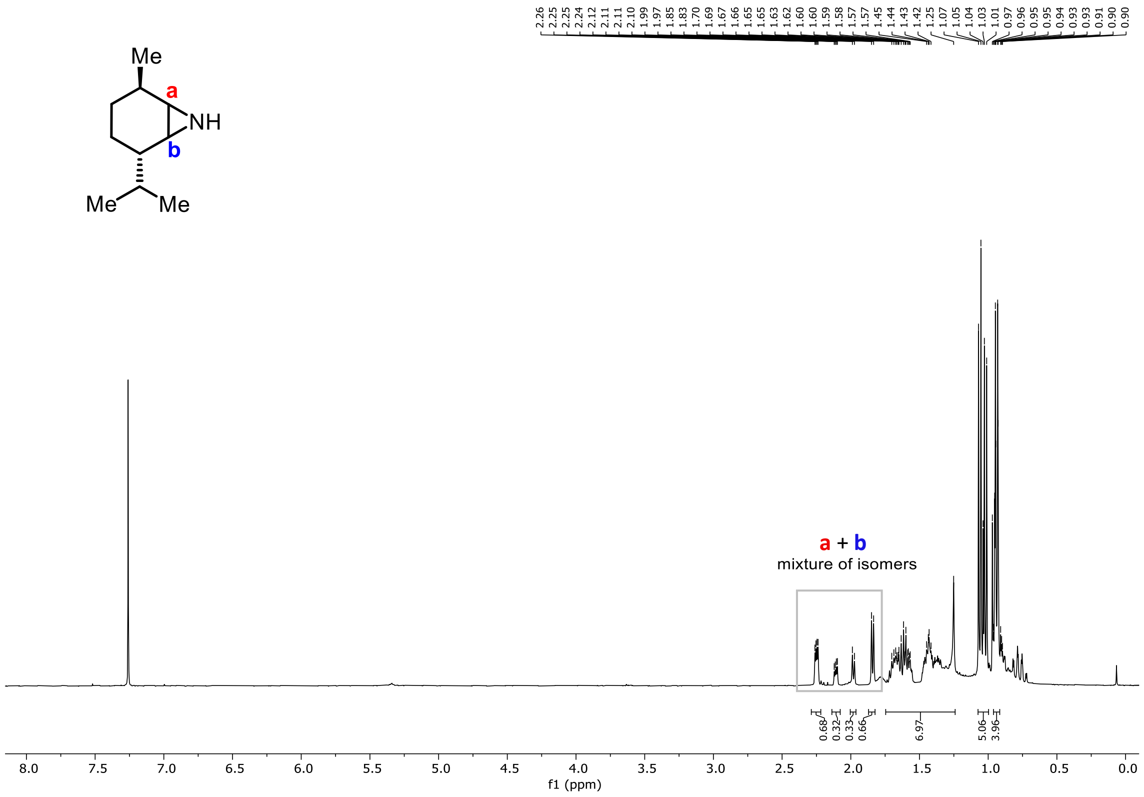

Figure S70. ^{1}H NMR spectrum ($CDCl_3$, 400 MHz) of compound **20**.

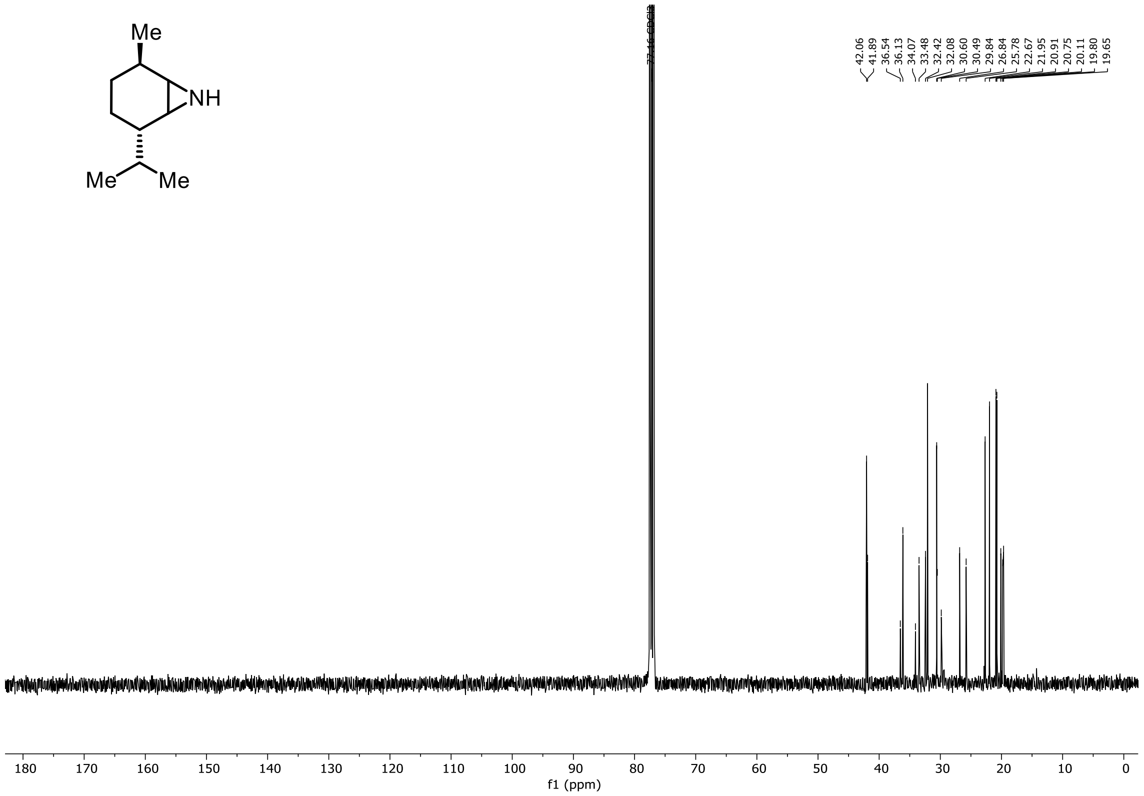

Figure S71. ^{13}C NMR spectrum ($CDCl_3$, 101 MHz) of compound **20**.

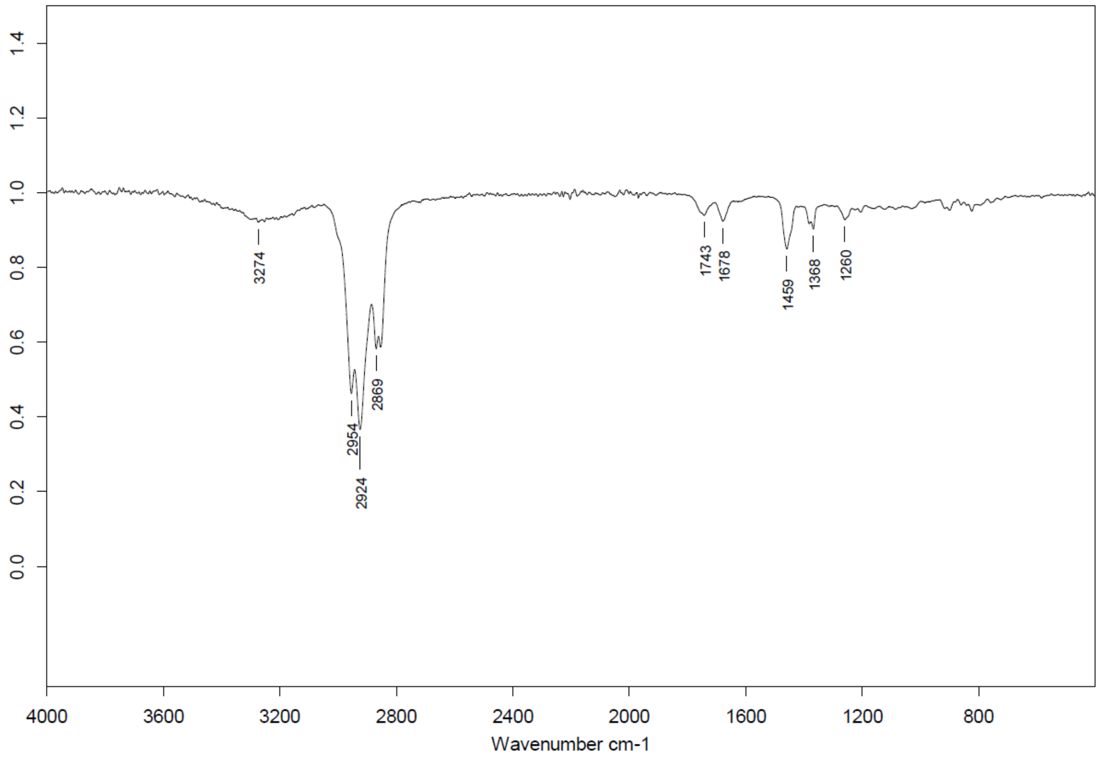

Figure S72. IR spectrum of compound **20**.

Analytical data for compound **21**

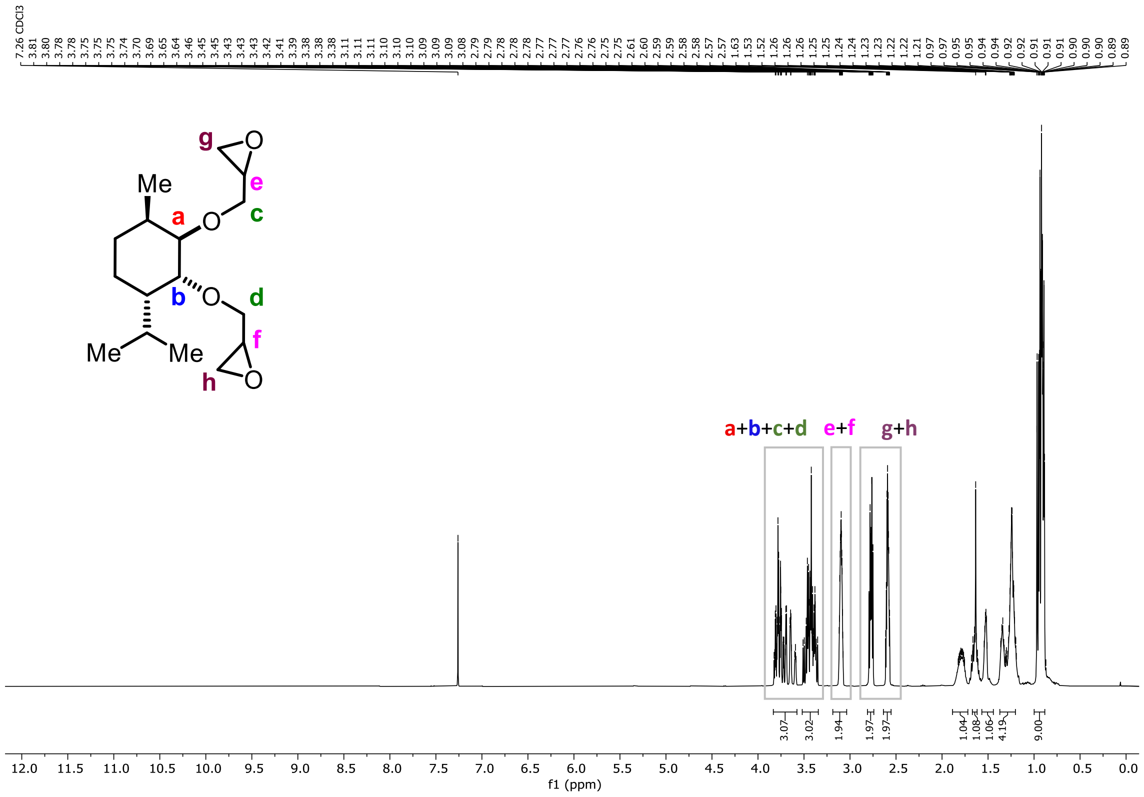

Figure S73. ^{1}H NMR spectrum ($CDCl_3$, 400 MHz) of compound **21**.

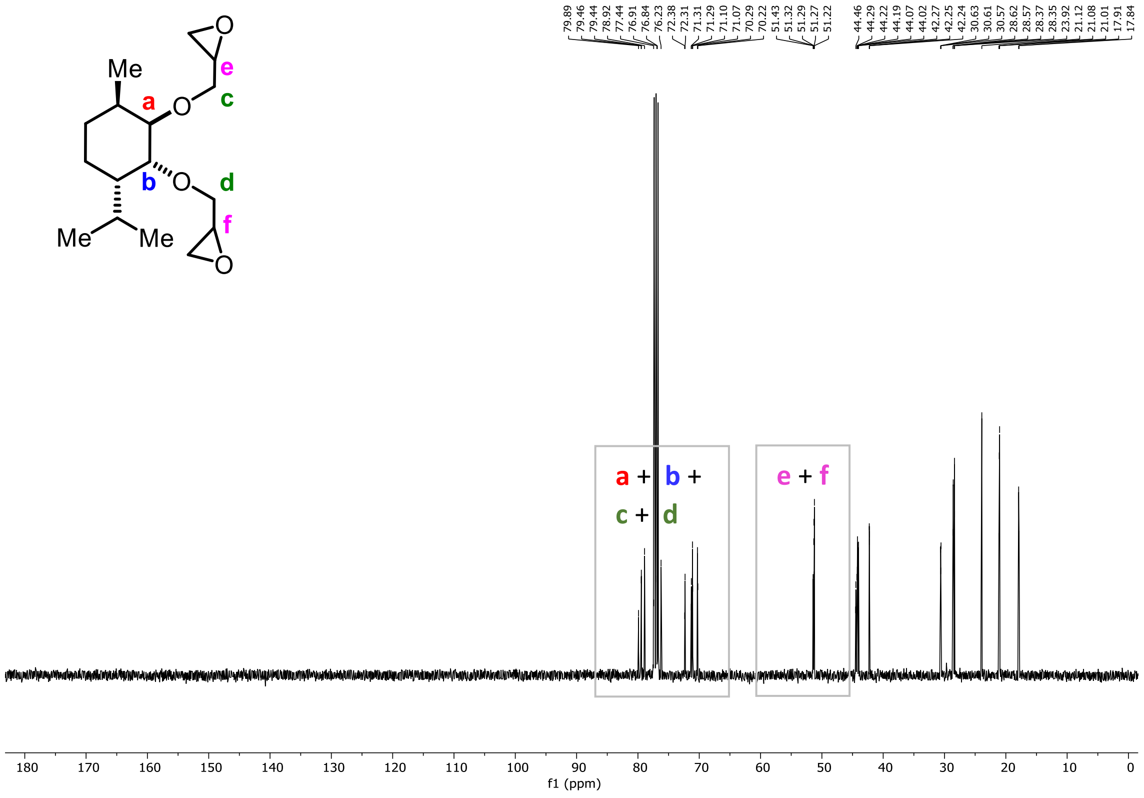

Figure S74. ^{13}C NMR spectrum ($CDCl_3$, 101 MHz) of compound **21**.

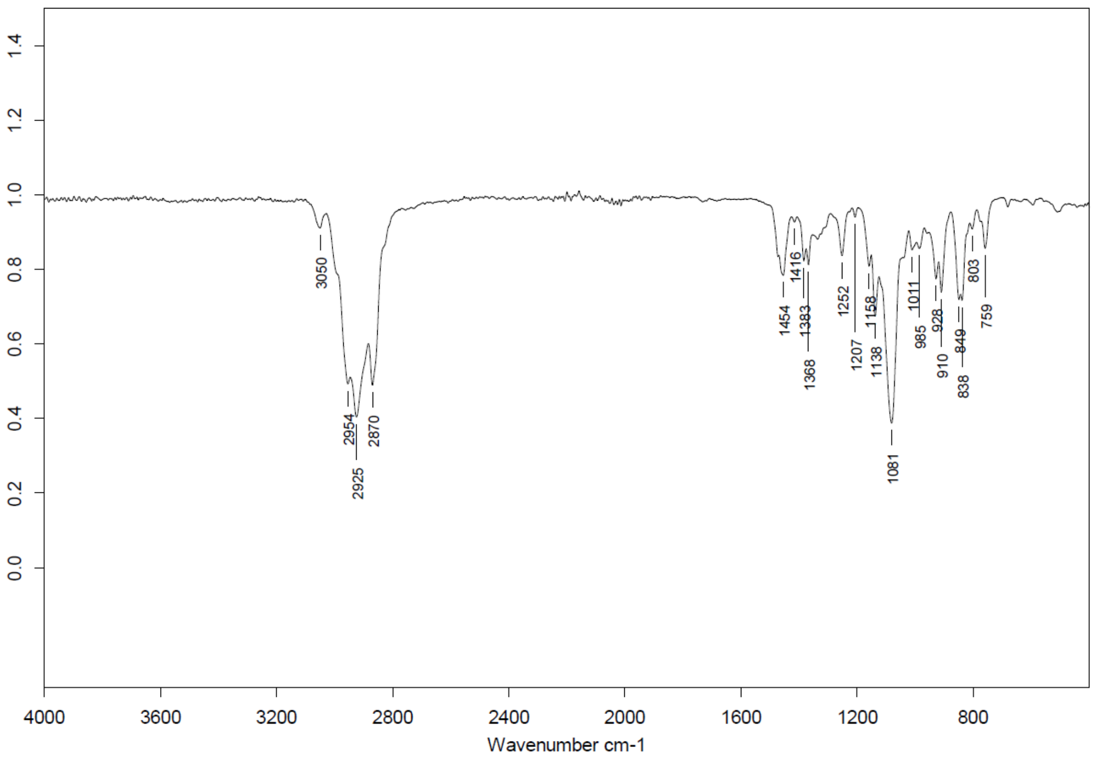

Figure S75. IR spectrum of compound **21**.

Analytical data for compound **22**

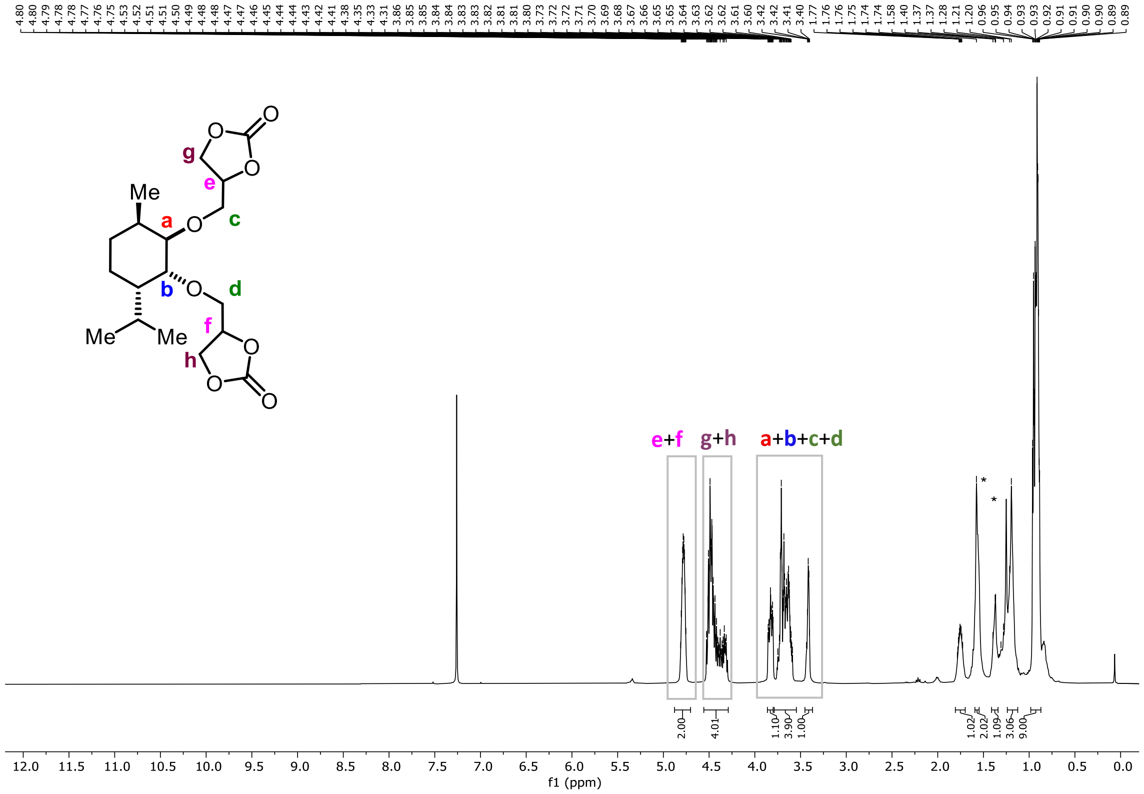

Figure S76. ^{1}H NMR spectrum ($CDCl_3$, 400 MHz) of compound **22**.

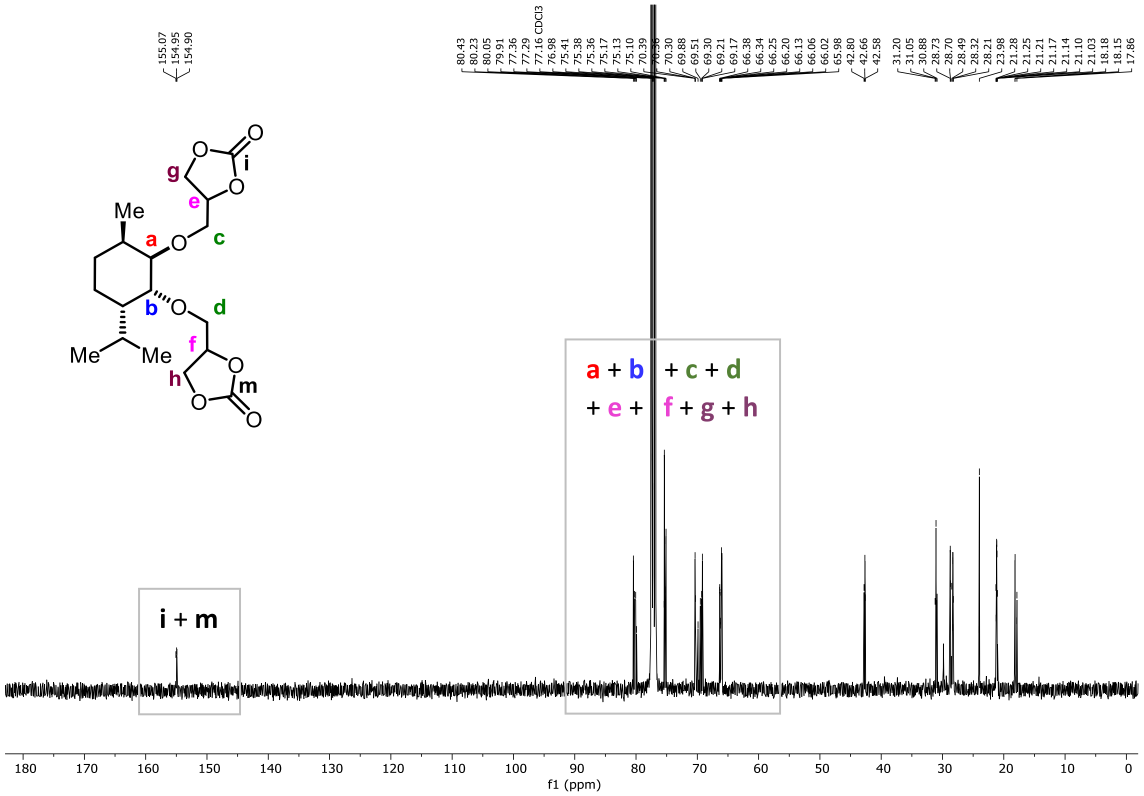

Figure S77. ^{13}C NMR spectrum ($CDCl_3$, 101 MHz) of compound **22**.

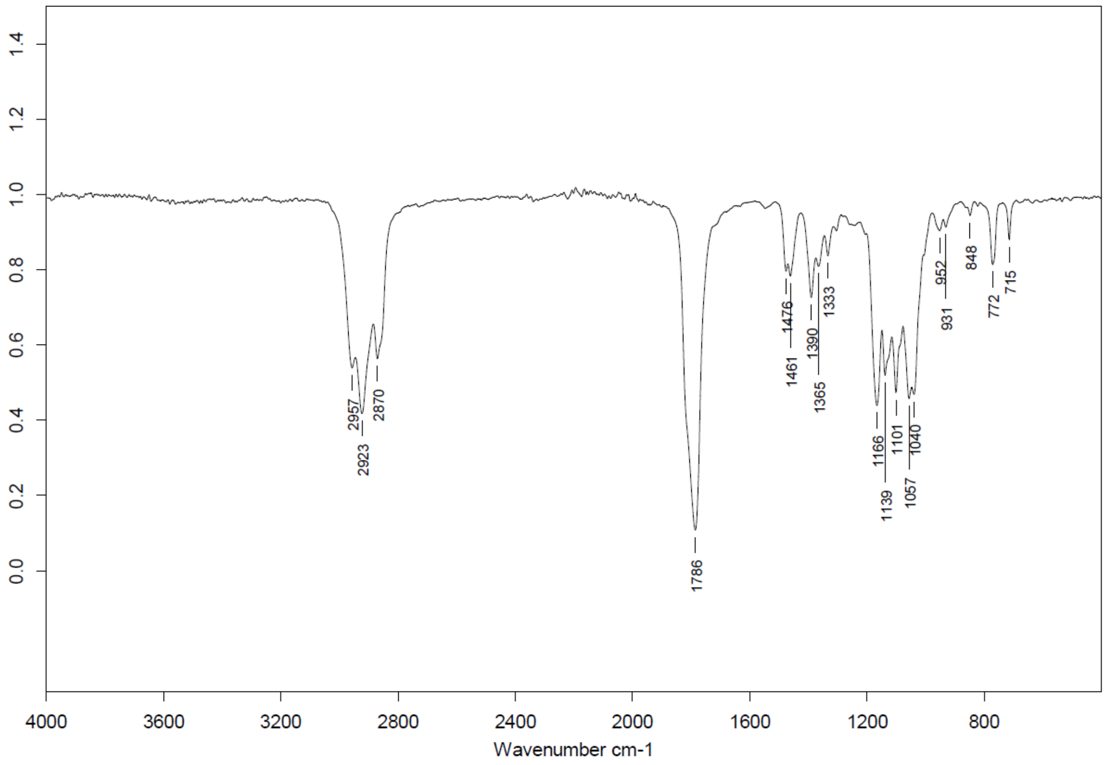

Figure S78. IR spectrum of compound **22**.

Analytical data for compound **23**

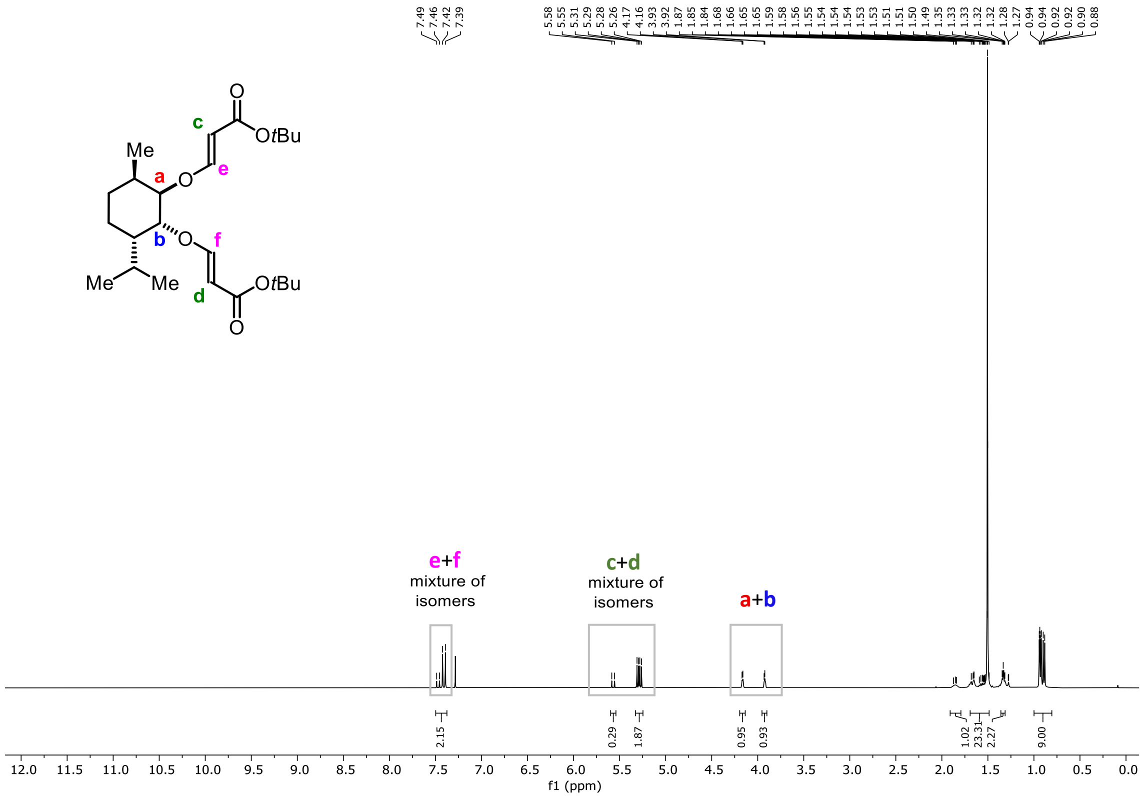

Figure S79. ^{1}H NMR spectrum ($CDCl_3$, 400 MHz) of compound **23**.

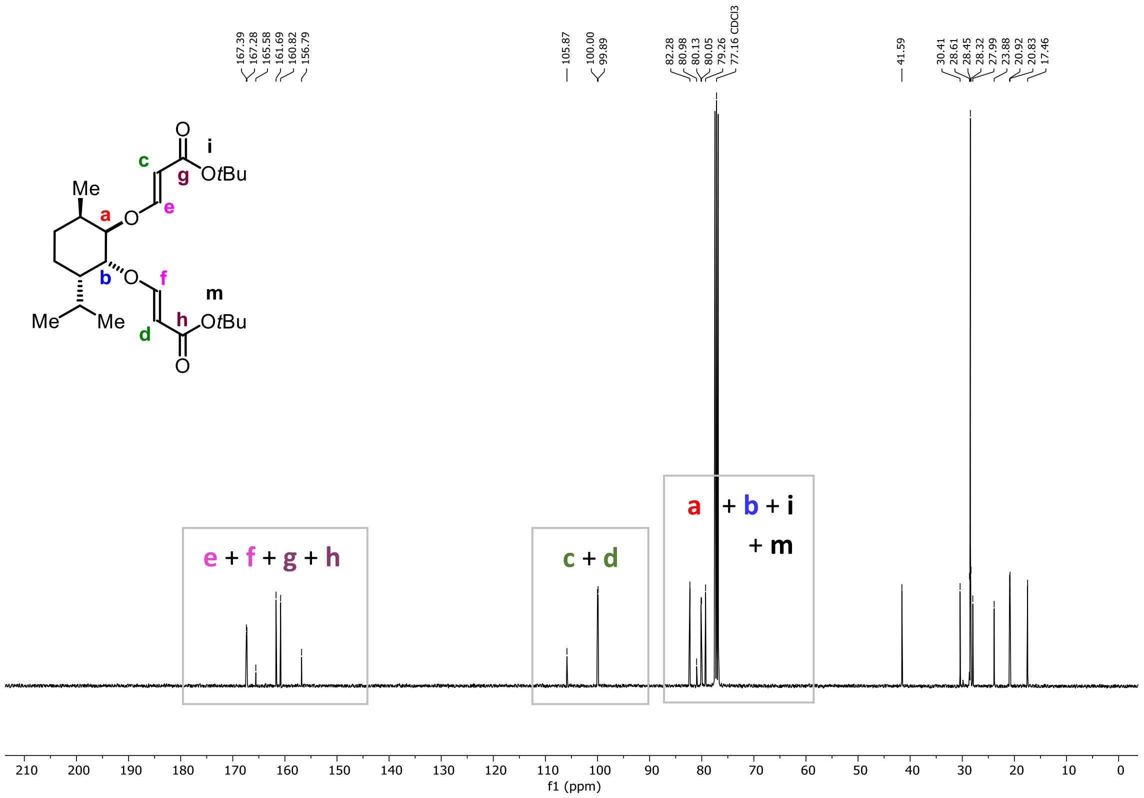

Figure S80. ^{13}C NMR spectrum ($CDCl_3$, 101 MHz) of compound **23**.

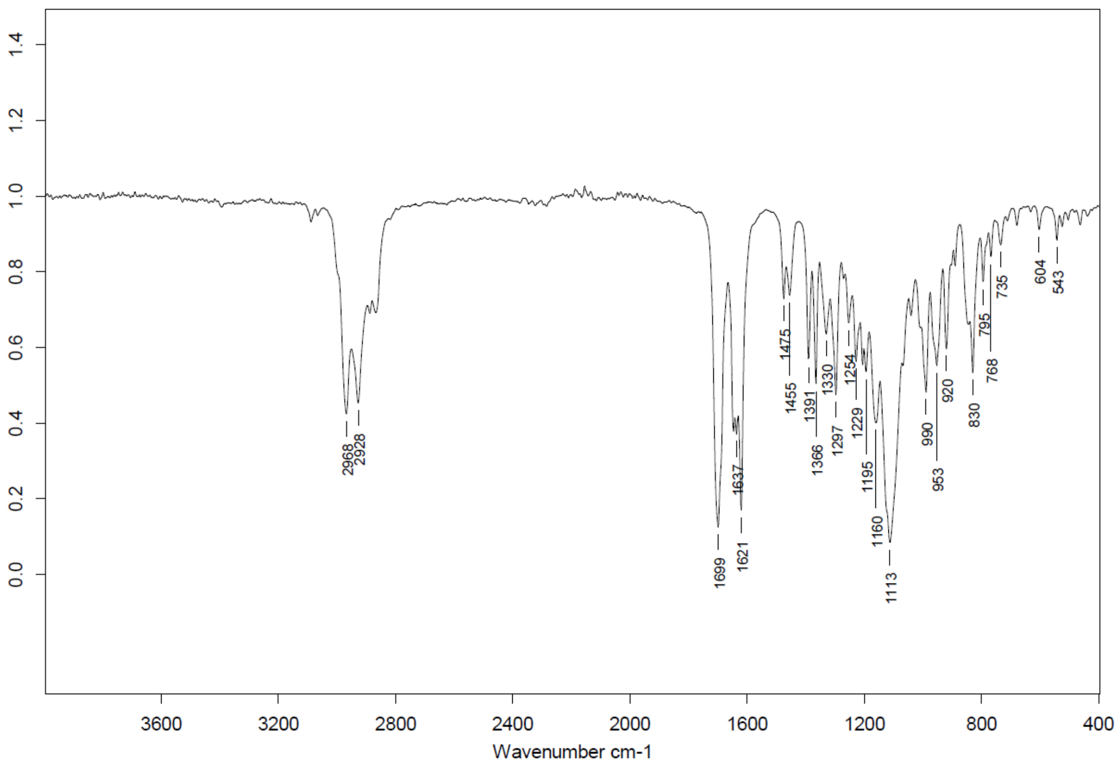

Figure S81. IR spectrum of compound **23**.

9. References

[1] T. Senthamarai, E. Lanaro, J. Tinker, A. Buchard, A. W. Kleij, *Polym. Chem.* **2025**, *16*, 2784-2790.

[2] C. J. Whiteoak, N. Kielland, V. Laserna, E. C. Escudero-Adán, E. Martin, A. W. Kleij, *J. Am. Chem. Soc.* **2013**, *135*, 1228–1231.

[3] T. Kiguchi, Y. Tsurusaki, S. Yamada, M. Aso, M. Tanaka, K. Sakai, H. Suemune, *Chem. Pharm. Bull.* **2000**, *48*, 1536-1540.

[4] A. Wambach, S. Agarwal, A. Greiner, *ACS Sustainable Chem. Eng.* **2020**, *8*, 14690–14693.

[5] L. Peña-Carrodeguas, C. Martín, A. W. Kleij, *Macromolecules* **2017**, *50*, 5337-5345.

[6] M. Szewczyk, M. Magre, V. Zubar, M. Rueping, *ACS Catal*. **2019**, *9*, 11634−11639.

[7] C. J. Whiteoak, N. Kielland, V. Laserna, E. C. Escudero-Adán, E. Martin, A. W. Kleij, *J. Am. Chem. Soc*. **2013**, *135*, 1228-1231.
